# The seahorse genome and the evolution of its specialized morphology

**DOI:** 10.1038/nature20595

**Published:** 2016-12-14

**Authors:** Qiang Lin, Shaohua Fan, Yanhong Zhang, Meng Xu, Huixian Zhang, Yulan Yang, Alison P. Lee, Joost M. Woltering, Vydianathan Ravi, Helen M. Gunter, Wei Luo, Zexia Gao, Zhi Wei Lim, Geng Qin, Ralf F. Schneider, Xin Wang, Peiwen Xiong, Gang Li, Kai Wang, Jiumeng Min, Chi Zhang, Ying Qiu, Jie Bai, Weiming He, Chao Bian, Xinhui Zhang, Dai Shan, Hongyue Qu, Ying Sun, Qiang Gao, Liangmin Huang, Qiong Shi, Axel Meyer, Byrappa Venkatesh

**Affiliations:** 1 CAS Key Laboratory of Tropical Marine Bio-resources and Ecology, South China Sea Institute of Oceanology, Chinese Academy of Sciences, Guangzhou, 510301 China; 2 Department of Biology, Chair in Zoology and Evolutionary Biology, University of Konstanz, Konstanz, 78457 Germany; 3 BGI-Shenzhen, Shenzhen, 518083 China; 4 Institute of Molecular and Cell Biology, A*STAR, Biopolis, 138673 Singapore Singapore; 5 College of Fisheries, Huazhong Agricultural University, Wuhan, 430070 China; 6 University of Chinese Academy of Science, Beijing, 100049 China; 7 School of Agriculture, Ludong University, Yantai, 264025 China; 8 Shenzhen Key Lab of Marine Genomics, Guangdong Provincial Key Lab of Molecular Breeding in Marine Economic Animals, BGI, Shenzhen, 518083 China; 9 Department of Paediatrics, Yong Loo Lin School of Medicine, National University of Singapore, Singapore, 119228 Singapore; 10 Present Address: † Present addresses: Department of Genetics, University of Pennsylvania, Pennsylvania 19104, USA (S.F.); Bioprocessing Technology Institute, Biopolis, Singapore 138668, Singapore (A.P.L.); Institute of Evolutionary Biology, the University of Edinburgh EH9 3FL, UK (H.M.G.); School of Material Science and Engineering, Nanyang Technological University, 50 Nanyang Avenue, Singapore 639798, Singapore (Z.W.L.)., ,

**Keywords:** Genome, Evolutionary developmental biology

## Abstract

Seahorses have a specialized morphology that includes a toothless tubular mouth, a body covered with bony plates, a male brood pouch, and the absence of caudal and pelvic fins. Here we report the sequencing and *de novo* assembly of the genome of the tiger tail seahorse, *Hippocampus comes*. Comparative genomic analysis identifies higher protein and nucleotide evolutionary rates in *H. comes* compared with other teleost fish genomes. We identified an astacin metalloprotease gene family that has undergone expansion and is highly expressed in the male brood pouch. We also find that the *H. comes* genome lacks enamel matrix protein-coding proline/glutamine-rich secretory calcium-binding phosphoprotein genes, which might have led to the loss of mineralized teeth. *tbx4*, a regulator of hindlimb development, is also not found in *H. comes* genome. Knockout of *tbx4* in zebrafish showed a ‘pelvic fin-loss’ phenotype similar to that of seahorses.

**Supplementary information:**

The online version of this article (doi:10.1038/nature20595) contains supplementary material, which is available to authorized users.

## Abstract (web summary)

Here, the genome sequence of the tiger tail seahorse is reported and comparative genomic analyses with other ray-finned fishes are used to explore the genetic basis of the unique morphology and reproductive system of the seahorse.

**Supplementary information:**

The online version of this article (doi:10.1038/nature20595) contains supplementary material, which is available to authorized users.

## Evolution at a gallop

Seahorses are prime examples of the exuberance of evolution and are unique among bony fish on several counts, including their equine body shape and male brood pouch. An international collaboration reporting in this issue of *Nature* has determined the genome sequence of a seahorse (*Hippocampus comes*, the tiger tail seahorse). They find it to be the most rapidly evolving fish genome studied so far. *H. comes* is among the most commonly traded seahorse species—dried for traditional medicines and live for the aquarium trade—and is on the IUCN Red List as a 'vulnerable' species. Analysis of the genomic sequence provides insights into the evolution of its unique morphology. Of note is the absence of a master control gene, *tbx4*, which functions in the development of hindlimbs and pelvic fins. Pelvic fins are missing in seahorses, and *tbx4*-knockout mutant zebrafish also lack pelvic fins.

**Supplementary information:**

The online version of this article (doi:10.1038/nature20595) contains supplementary material, which is available to authorized users.

## Main

**Figure 1:**
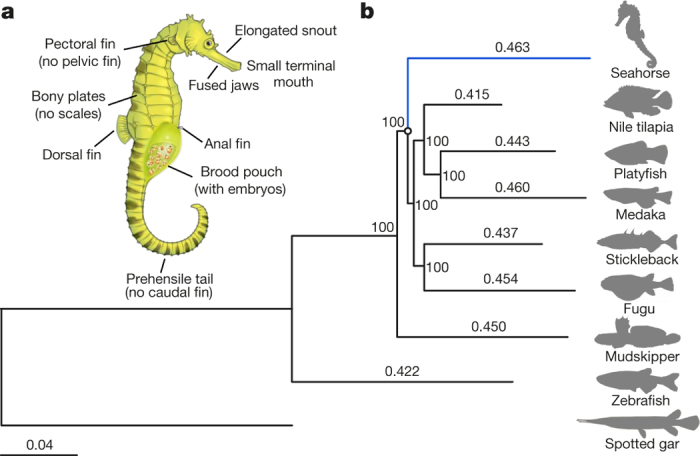
Adaptations and evolutionary rate of *H. comes*. **a**, Schematic diagram of a pregnant male seahorse. **b**, The phylogenetic tree generated using protein sequences. The values on the branches are the distances (number of substitutions per site) between each of the teleost fishes and the spotted gar (outgroup). Spotted gar, *Lepisosteus oculatus*; zebrafish, *Danio rerio*. PowerPoint slide

**Extended Data Figure 1:**
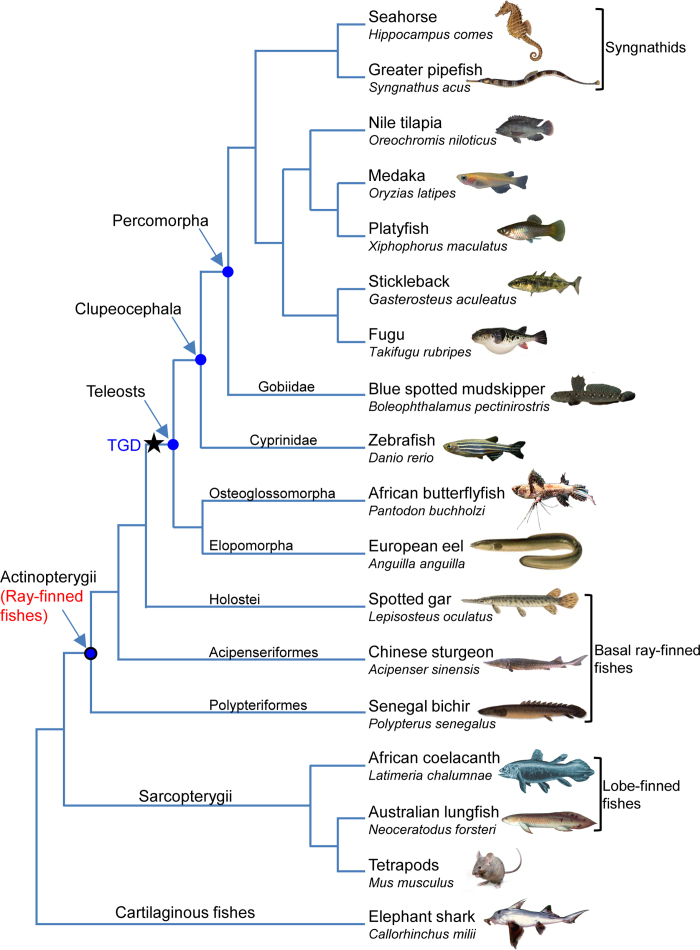
Phylogenetic relationships of ray-finned fishes discussed in this study. Phylogenetic relationships of ray-finned fishes depicted here are based on the current study and ref. 59. Ray-finned fishes (Actinopterygii) are divided into basal ray-finned fishes (Polypteriformes, Acipenseriformes and Holostei) and teleosts. The latter comprise ~99% of the extant ray-finned fishes. The star represents the teleost-specific genome duplication (TGD) event that occurred in the common ancestor of all teleost fishes. Syngnathids (seahorse and pipefish) display the unique phenomenon of ‘male pregnancy’.

Members of the teleost family Syngnathidae (seahorses, pipefishes and seadragons) (Extended Data Fig. 1), comprising approximately 300 species, display a complex array of morphological innovations and reproductive behaviours. This includes specialized morphological phenotypes such as an elongated snout with a small terminal mouth, fused jaws, absent pelvic and caudal fins, and an extended body covered with an armour of bony plates instead of scales^1^ (Fig. 1a). Syngnathids are also unique among vertebrates due to their ‘male pregnancy’, whereby males nourish developing embryos in a brood pouch until hatching and parturition occurs^2,3^. In addition, members of the subfamily Hippocampinae (seahorses) exhibit other derived features such as the lack of a caudal fin, a characteristic prehensile tail, and a vertical body axis^4^ (Fig. 1a). To understand the genetic basis of the specialized morphology and reproductive system of seahorses, we sequenced the genome of the tiger tail seahorse, *H. comes*, and carried out comparative genomic analyses with the genome sequences of other ray-finned fishes (Actinopterygii).

## Genome assembly and annotation

The genome of a male *H. comes* individual was sequenced using the Illumina HiSeq 2000 platform. After filtering low-quality and duplicate reads, 132.13 Gb (approximately 190-fold coverage of the estimated 695 Mb genome) of reads from libraries with insert sizes ranging from 170 bp to 20 kb were retained for assembly. The filtered reads were assembled using SOAPdenovo (version 2.04) to yield a 501.6 Mb assembly with an N50 contig size and N50 scaffold size of 34.7 kb and 1.8 Mb, respectively. Total RNA from combined soft tissues of *H. comes* was sequenced using RNA-sequencing (RNA-seq) and assembled *de novo*. The *H. comes* genome assembly is of high quality, as >99% of the *de novo* assembled transcripts (76,757 out of 77,040) could be mapped to the assembly; and 243 out of 248 core eukaryotic genes mapping approach (CEGMA) genes are complete in the assembly.

**Extended Data Figure 2:**
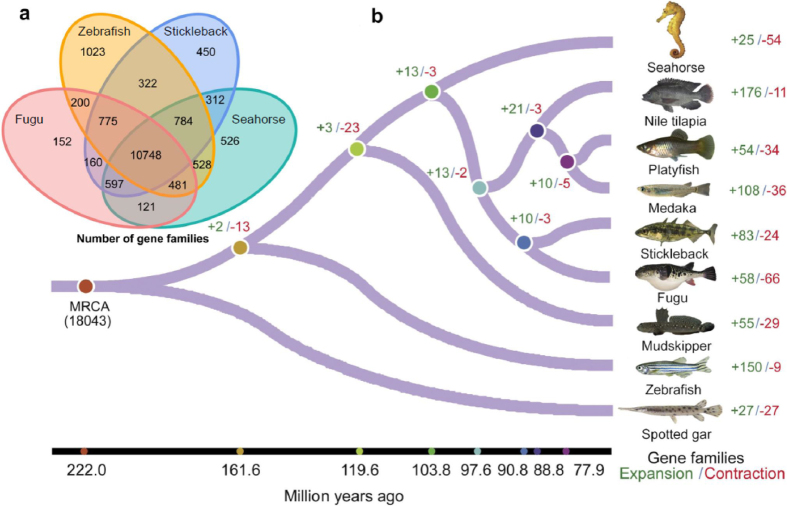
Number of gene families in various teleosts and the spotted gar. **a**, Venn diagram of shared orthologous gene families in seahorse (*H. comes*), fugu, zebrafish and stickleback. **b**, The phylogeny and divergence times of seahorse and other teleost fishes based on analysis of genome-wide one-to-one orthologous protein sequences. The numbers at nodes indicate the number of gene families expanded and contracted at different evolutionary time points.

**Extended Data Figure 3:**
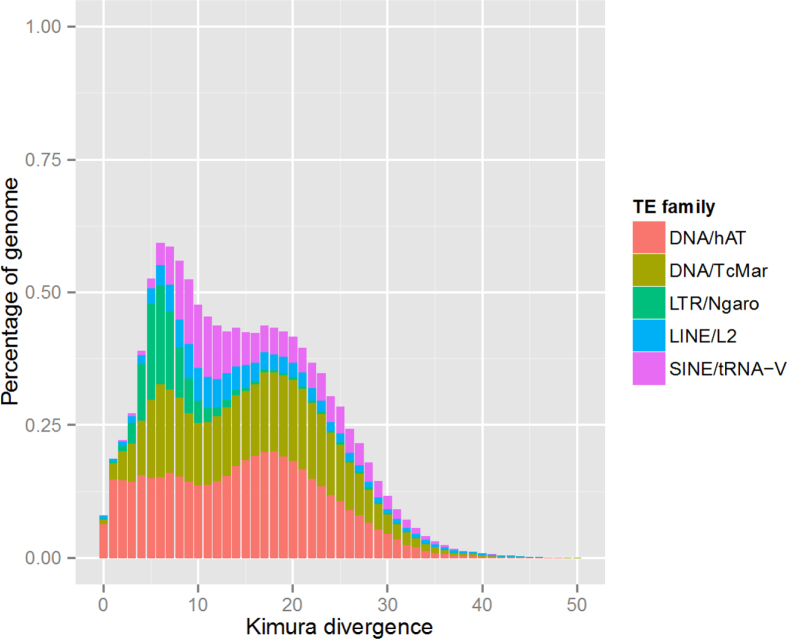
Divergence distribution of transposable elements compared to consensus in the transposable element library. The divergence rate was calculated between the identified transposable elements (TEs) in the *H. comes* genome and the consensus sequence in the transposable element library.

We predicted 23,458 genes in the genome of *H. comes* based on homology and by mapping the RNA-seq data of *H. comes* and a closely related species, the lined seahorse, *Hippocampus erectus*, to the genome assembly (see Methods and Supplementary Information). More than 97% of the predicted genes (22,941 genes) either have homologues in public databases (Swissprot, Trembl and the Kyoto Encyclopedia of Genes and Genomes (KEGG)) or are supported by assembled RNA-seq transcripts. Analysis of gene family evolution using a maximum likelihood framework identified an expansion of 25 gene families (261 genes; 1.11%) and contraction of 54 families (96 genes; 0.41%) in the *H. comes* lineage (Extended Data Fig. 2 and Supplementary Tables 4.1, 4.2). Transposable elements comprise around 24.8% (124.5 Mb) of the *H. comes* genome, with class II DNA transposons being the most abundant class (9%; 45 Mb). Only one wave of transposable element expansion was identified, with no evidence for a recent transposable element burst (Kimura divergence ≤ 5) (Extended Data Fig. 3).

## Phylogenomics and evolutionary rate

The phylogenetic relationships between *H. comes* and other teleosts were determined using a genome-wide set of 4,122 one-to-one orthologous genes (Supplementary Note 4.2). The phylogenetic analysis (Fig. 1b) showed that *H. comes* is a sister group to other percomorph fishes analysed (stickleback, *Gasterosteus aculeatus*; medaka, *Oryzias latipes*; Nile tilapia, *Oreochromis niloticus*; fugu, *Takifugu rubripes*; and platyfish, *Xiphophorus maculatus*) with the exception of blue-spotted mudskipper (*Boleophthalmus pectinirostris*), a member of the family Gobiidae. Our inference, which placed the mudskipper as the outgroup, differs from that of a previous phylogenetic analysis based on fewer protein-coding genes that had placed syngnathids as an outgroup^5^. Estimated divergence times of *H. comes* and other teleosts calculated using MCMCTree suggest that *H. comes* diverged from the other percomorphs approximately 103.8 million years ago, during the Cretaceous period (Extended Data Fig. 2). Interestingly, the branch length of *H. comes* is longer than that of other teleosts, suggesting a higher protein evolutionary rate compared to other teleosts analysed in this study (Fig. 1b). This result was found to be statistically significant by both relative rate test^6^ and two cluster analysis^7^ (Supplementary Tables 4.3 and 4.4). To determine whether the neutral nucleotide substitution rate of *H. comes* is also higher, we generated a neutral tree on the basis of fourfold degenerate sites and calculated the pairwise distance of each teleost to the spotted gar (an outgroup) (Supplementary Fig. 4.4). The pairwise distance of *H. comes* was again higher compared with other teleosts, indicating that the neutral evolutionary rate of *H. comes* is also higher than that of other teleosts. The reasons for this higher molecular evolutionary rate in *H. comes* are unclear.

## Gene loss

Gene loss or loss of function can contribute to evolutionary novelties and can be positively selected for^8,9^. We identified several genes that are not found in the *H. comes* genome but are found in other sequenced teleost genomes.

**Extended Data Figure 4:**
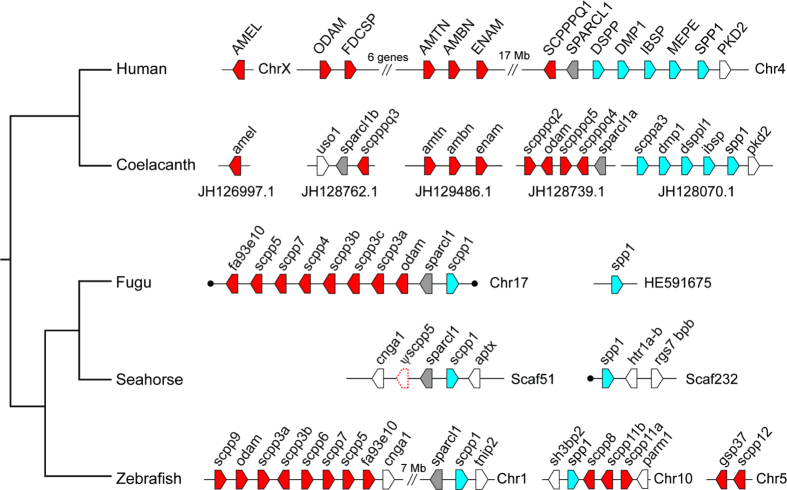
SCPP genes in *H. comes* and other jawed vertebrates. Gene loci for human, coelacanth and zebrafish were adapted from other publications^60,61^. *sparcl1*, which is the ancestral gene that gave rise to SCPP genes is shown in grey; P/Q-rich SCPP genes are shown in red; acidic SCPP genes are shown in blue. In seahorse, *scpp5* is a pseudogene and is denoted by ψ. Owing to space constraints, the P/Q-rich SCPP genes encoding milk casein and salivary proteins in human have been omitted. Black circles mark the ends of scaffolds.

Secretory calcium-binding phosphoprotein (SCPP) genes encode extracellular matrix proteins that are involved in the formation of mineralized tissues such as bone, dentin, enamel and enameloid. Bony vertebrate genomes encode multiple SCPP genes that can be divided into two groups, the acidic and the proline/glutamine (P/Q)-rich SCPP genes. Acidic SCPPs regulate the mineralization of collagen scaffolds in bone and dentin whereas the P/Q-rich SCPPs are primarily involved in enamel or enameloid formation^10^. Analysis of the *H. comes* genome and the transcriptomes of *H. comes* and *H. erectus* showed that both contain two acidic SCPP genes, *scpp1* and *spp1* (Extended Data Fig. 4). However, no intact P/Q-rich gene could be identified. The only P/Q-rich gene present in the *H. comes* genome assembly, *scpp5*, is represented by only three out of ten exons, indicating that it has become a pseudogene. Seahorses and pipefish (family Syngnathidae) are toothless, a phenomenon known as edentulism. Besides syngnathids, edentulism has occurred convergently in several other vertebrate lineages^11^, the most notable ones being birds^12^, turtles, and some mammals such as baleen whales, pangolins and anteaters^13^. The loss of teeth in birds, turtles and mammals has been attributed to inactivating mutations in one or more P/Q-rich enamel-specific SCPP genes such as *Enam*, *Amel*, *Ambn* and *Amtn*, and the dentin-specific gene, *Dspp*^12,14^. In the case of *H. comes*, the complete loss of functional P/Q-rich SCPP genes may explain the loss of mineralized teeth.

**Figure 2:**
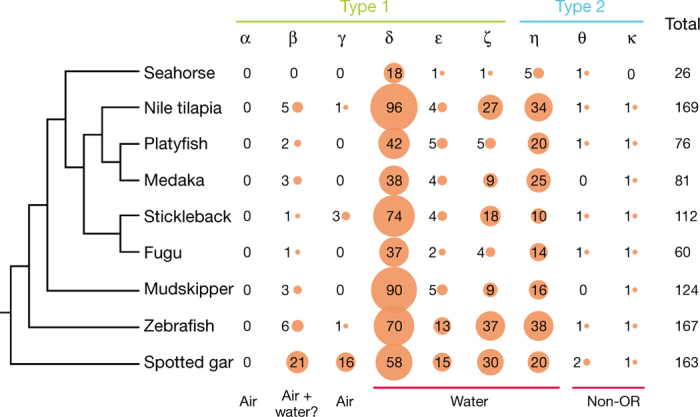
OR genes in *H. comes* and other ray-finned fishes. ‘Air’ and ‘water’ refer to the detection of airborne and water-soluble odorants, respectively. The sizes of the orange circles represent the number of OR genes of a particular category. PowerPoint slide

**Extended Data Figure 5:**
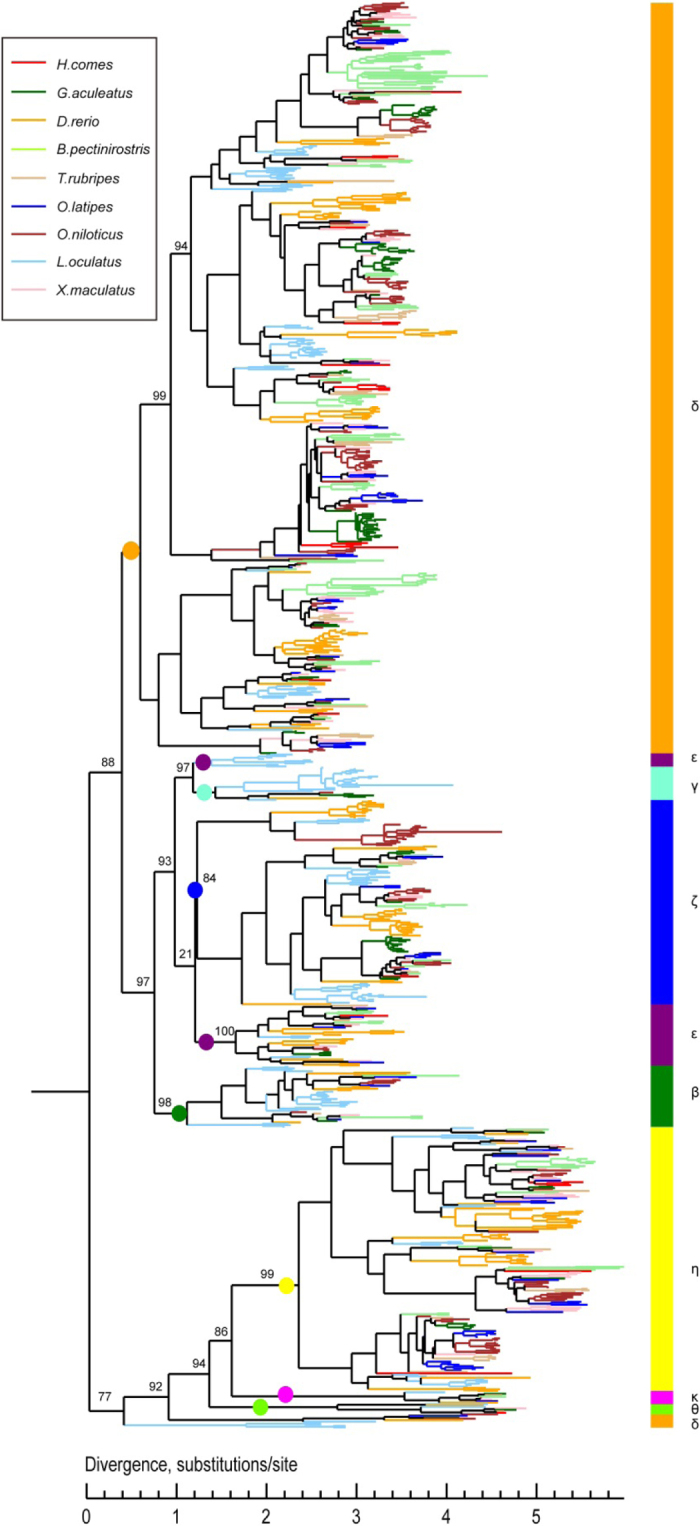
Maximum-likelihood phylogenetic tree of OR genes in *H. comes* and other ray-finned fishes.

Animals use their sense of smell, or olfaction, for finding food, mates and avoiding predators. Olfaction is mediated by olfactory receptors (ORs), which constitute the largest family of G-protein-coupled receptors. We were able to identify in the *H. comes* genome a significantly smaller repertoire of OR genes than in other teleosts (*P* value < 0.05, Wilcoxon rank-sum test). Our sensitive search pipeline (based on TblastN and Genewise) and manual inspection identified only 26 OR genes in the *H. comes* genome—the smallest OR repertoire identified in any ray-finned fish genome analysed so far (60 to 169 OR genes) (Fig. 2 and Extended Data Fig. 5).

A derived phenotype of seahorse and other syngnathids is the complete lack of pelvic fins^15,16^. Pelvic fins are homologous to tetrapod hindlimbs and primarily serve a role in body trim and subtle swimming manoeuvres during teleost locomotion^17,18,19^. In addition, pelvic spines have an important role in protection against predators^15^. Pelvic fin loss has occurred independently in several teleost lineages, including Tetraodontidae (for example, pufferfishes), Anguillidae (eels) and Gasterosteidae (some populations of sticklebacks), and is frequently associated with a reduced pressure from predators and/or the evolution of an elongated body plan^15^. In pufferfish (fugu), pelvic fin loss is associated with a change in the expression pattern of *hoxd9a*^20^. In freshwater populations of stickleback, the loss of pelvic fins has been demonstrated to be due to deletions in the pelvic fin-specific enhancer of *pitx1* (ref. 21).

**Figure 3:**
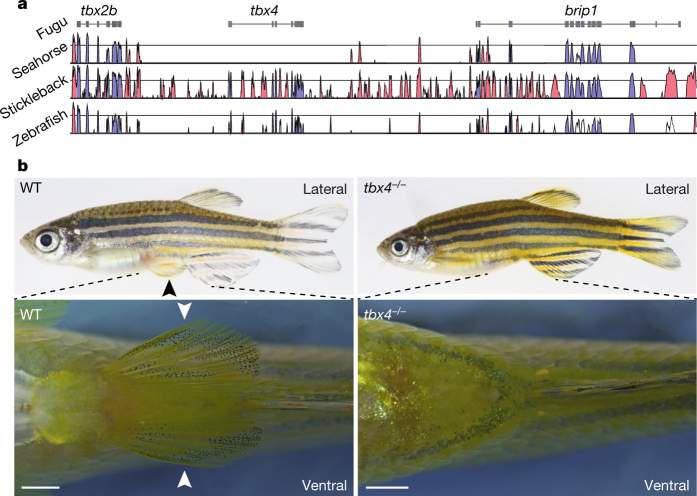
Pelvic fin loss in *H. comes* is associated with loss of *tbx4*. **a**, Vista plot of conserved elements in the *tbx2b*-*tbx4*-*brip1* syntenic region in fugu (reference genome), seahorse (*H. comes*), stickleback and zebrafish showing that *tbx4* is missing from this locus in seahorse. The blue and red peaks represent conserved exonic and non-coding sequences, respectively. **b**, Lateral (top) and ventral view (bottom) of wild-type (WT) and a representative (one out of five) F3 homozygous *tbx4*-null mutant (*tbx4*^*−/−*^) zebrafish. Bottom panel shows a close-up of the pelvic region (dashed lines indicate the approximate zoom region). Scale bar, 1 mm. Pelvic fins are indicated with black or white arrowheads in the wild-type fish. Homozygous *tbx4*-null mutants entirely lack pelvic fins without showing any other gross morphological defects. PowerPoint slide

**Extended Data Figure 6:**
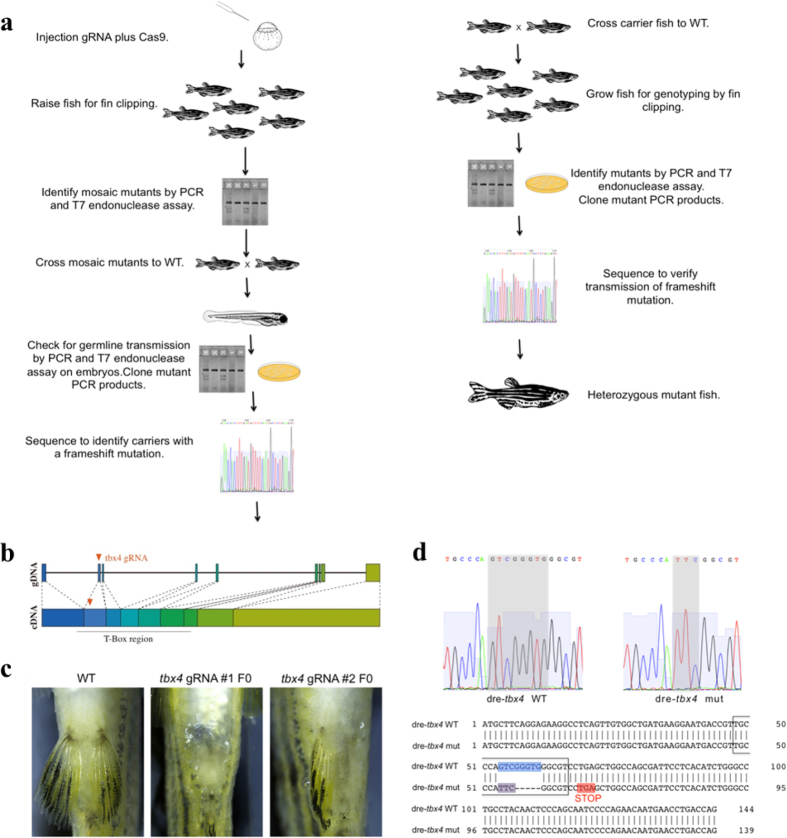
CRISPR–Cas9 mediated knockdown of *tbx4* in zebrafish. **a**, CRISPR–Cas9 mutagenesis strategy. **b**, CRISPR–Cas9 sites targeted in zebrafish *tbx4* gene. **c**, Loss of function *tbx4* phenotypes in F0 mosaic mutants. Pelvic fin loss was observed with low frequency in F0 mosaic mutant fish. Frequency of animals with either single- or double-sided loss of pelvic fins was 3/42 for gRNA#1 and 1/34 for gRNA#2. **d**, Identification of zebrafish *tbx4* mutant line. Top shows sequencing chromatograms of wild-type (left) and mutant (right) alleles. Bottom shows alignment of *tbx4* exon 2 from wild-type and mutant. The region for which the chromatograms are shown is indicated with a box. In the mutant a deletion (indicated in blue in the wild-type sequence)/substitution (indicated in lilac in the mutant sequence) was identified. The deletion/substitution area is indicated with a grey box in the chromatograms.

Analysis of the *H. comes* genome and the transcriptomes of *H. comes* and *H. erectus* (see Supplementary Information, section 2), suggested that *tbx4*, a transcription factor conserved in jawed vertebrates, is not present in the seahorse genome (Fig. 3a) (Supplementary Information, section 9). To verify this, we carried out degenerate polymerase chain reaction (PCR) using genomic DNA from *H. comes* and several other species of syngnathids and some non-syngnathids. While the degenerate primers amplified a fragment of *tbx4* from non-syngnathids, they failed to amplify a *tbx4* fragment from syngnathid fishes (see Supplementary Information, section 9). Tbx4 is a T-box DNA-binding domain-containing transcription factor that acts as a regulator of hindlimb formation in mammals^22,23,24^. Loss of function of this gene in mouse leads to a failure of hindlimb formation^22,23^ as well as strong pleiotropic defects in lung^25^ and placental development^22^. Expression of zebrafish *tbx4* specifically in pelvic fins suggests a similar role in appendage patterning in fishes^24^. Given the major role of *tbx4* in hindlimb formation in mammals, we hypothesized that its absence in *H. comes* might be associated with the loss of pelvic fins. To test this hypothesis, we generated a CRISPR–Cas9 *tbx4*-knockout mutant zebrafish line. Interestingly, unlike homozygous mouse *Tbx4* mutants, which fail to develop a functional allantois^22^, the homozygous zebrafish mutants are viable but completely lack pelvic fins without exhibiting any other gross morphological abnormalities in pectoral or median fins (Fig. 3c and Extended Data Fig. 6; see also Supplementary Information, section 9.3, in particular Supplementary Fig. 9.6 for additional phenotype analysis). This finding is consistent with the results of a recent study that showed that mutations in *tbx4* are associated with the loss of pelvic fins in a naturally occurring zebrafish strain called *pelvic finless*^26^ (see also Supplementary Information, section 9.3). These results show that *tbx4* has a role in pelvic fin formation in teleosts and suggests that the loss of pelvic fins in *H. comes* may be related to the loss of *tbx4*.

## Expansion of the *patristacin* gene family

Male pregnancy is an evolutionary innovation unique to syngnathids. In teleosts, the C6AST subfamily of astacin metalloproteases—such as high choriolytic enzyme (HCE) and low choriolytic enzyme (LCE)—are involved in lysing the chorion surrounding the egg, leading to hatching of embryos^27^. A member of this subfamily, *patristacin* (*pastn*), was found to be highly expressed in the brood pouch of pregnant males of the Gulf pipefish, *Syngnathus scovelli*, leading to the suggestion that this gene may have a role in the evolution of male pregnancy^28^. A *pastn* gene was also found to be highly expressed in the brood pouch of the male big belly seahorse, *H. abdominalis*, during mid- and late pregnancy^29^, suggesting a shared role for this gene in male pregnancy in syngnathids.

**Figure 4:**
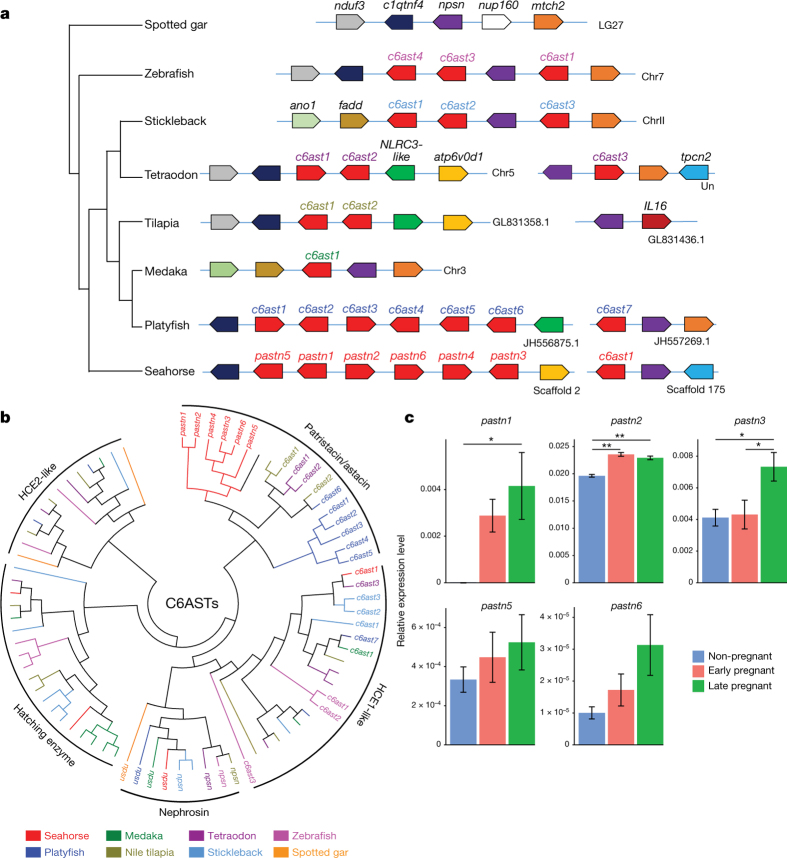
Astacin metalloproteinase gene family in ray-finned fishes. **a**, Astacin gene loci in various ray-finned fish genomes showing expansion of *pastn* genes in seahorse (*H. comes*) and *c6ast* genes in platyfish. Chr, chromosome. **b**, The phylogeny of the astacin gene family in ray-finned fishes. Only *pastn* or *c6ast* genes shown in **a** are labelled. Supplementary Fig. 10.1 shows an expanded version of the tree with all the genes labelled. **c**, Expression patterns of *pastn* genes in relation to 18S ribosomal RNA genes in the brood pouch of male *H. erectus* determined by qRT–PCR. All data are expressed as mean ± standard error of mean (*n* = 5) and evaluated by one-way analysis of variance (ANOVA) followed by Tukey’s honestly significant difference test for adjusting *P* values from multiple comparisons (see Methods and Supplementary Information for details of methods). The average duration of pregnancy (from fertilization to parturition) is 17 days^41^. The *y* axis represents expression level in relation to 18S rRNA genes. *pastn1* is expressed at low levels at the non-pregnant stage, which is not clearly visible in the figure due to the large scale used. Non-pregnant: no embryos in the brood pouch; early pregnant: 2–4 days post-fertilization; late pregnant: 12–14 days post-fertilization. *P < 0.05, **P < 0.01. Note that *pastn4* is not expressed in these stages of brood pouch. PowerPoint slide

The *H. comes* genome contains six *pastn* genes (*pastn1* to *pastn6*; Fig. 4a) organized in a cluster. To examine their expression patterns in the brood pouch, we carried out RNA-seq analysis at different stages of brood pouch development (see Supplementary Information, section 2) in *H. erectus*, as this species is easy to obtain and breed in the laboratory. *H. comes* and *H. erectus* exhibit very similar reproductive cycles and their coding sequences are highly similar (average identity of 93.3%; determined by aligning *H. erectus* RNA-seq transcripts to the *H. comes* genome assembly). We identified orthologues for five of the *H. comes pastn* genes (*pastn1*, *pastn2*, *pastn3*, *pastn5* and *pastn6*) in the RNA-seq transcripts of *H. erectus* (Supplementary Fig. 2). Quantitative reverse transcription PCR (qRT–PCR) analysis of these genes showed that some of them are expressed at significantly higher levels in early- and late-pregnant stages (Fig. 4c). For example, *pastn2* is expressed at significantly higher levels in early- and late-pregnant stages compared to the non-pregnant stage, whereas *pastn1* and *pastn3* are expressed at significantly higher levels during the late-pregnant stage compared to non-pregnant stage (Fig. 4c). This expression pattern suggests a role for these *pastn* genes in brood pouch development and/or hatching of embryos within the brood pouch prior to parturition.

Interestingly, the platyfish (*X. maculatus*), in which fertilization and hatching of eggs occur within the maternal body (ovoviviparity), contains a cluster of six *c6ast* genes (Fig. 4a), with potential hatching enzyme-like activity^30^. Phylogenetic analysis of *c6ast* family genes in *H. comes*, platyfish and other fishes showed that *H. comes pastn* genes and platyfish *c6ast* genes form separate clades (Fig. 4b), indicating that they have expanded independently in the two lineages. Thus, this is an interesting instance of a gene family (C6AST subfamily of astacin metalloproteases) that has undergone expansion independently in different teleost lineages and shows new expression patterns and functions associated with similar evolutionary innovations (that is, ovoviviparity in female platyfish and male pregnancy in seahorse).

## Loss of conserved noncoding elements

Vertebrate genomes contain thousands of noncoding elements that are under purifying selection^31,32,33^. Many of these conserved noncoding elements (CNEs) function as *cis*-regulatory elements such as enhancers, repressors and insulators^34,35^. Evolutionary loss of CNEs has important roles in phenotypic differences and morphological innovations^21,36,37^. To determine the extent of loss of CNEs in seahorse, we predicted genome-wide CNEs in *H. comes* and four other percomorph fishes (stickleback, fugu, medaka and Nile tilapia) using zebrafish as the reference genome (see Supplementary Information). We identified 239,976 CNEs (average size of 168 bp) that are conserved in zebrafish and at least one of the five percomorph fishes (Supplementary Table 6.1). To determine the extent to which CNEs are lost in *H. comes*, we searched for CNEs that are uniquely lost in each of the percomorph fishes. We restricted our analyses to a high-confidence set of CNEs situated in gap-free syntenic intervals (Supplementary Table 6.5). Interestingly, *H. comes* was found to have lost a substantially higher number of CNEs (1,612 CNEs) compared to other percomorphs (fugu, 1,050 CNEs; stickleback, 843 CNEs; medaka, 335 CNEs; Nile tilapia, 281 CNEs) (Supplementary Table 6.6).

Analysis of zebrafish CNEs that are lost in *H. comes* indicated that they are present in the neighbourhood of 728 genes enriched in functions such as regulation of transcription, regulation of the fibroblast growth factor receptor signalling pathway, embryonic pectoral fin morphogenesis, steroid hormone receptor activity and O-acetyltransferase activity (Supplementary Tables 6.8 and 6.9). The top 20 genes adjacent to regions with the highest number of CNEs lost in *H. comes* include *sall1a*, *shox* and *irx5a* (Supplementary Tables 6.10 and 6.11), which are involved in the development of the limbs, nervous system, kidney, heart and skeletal system. Altered expression patterns of these genes can potentially lead to altered morphological phenotypes. For example, loss of regulatory regions of the human *SHOX* gene is the cause of Leri–Weill dyschondrosteosis, a dominantly inherited skeletal dysplasia that is characterized by moderate short stature caused by short mesomelic limb segments^38,39^.

**Extended Data Figure 7:**
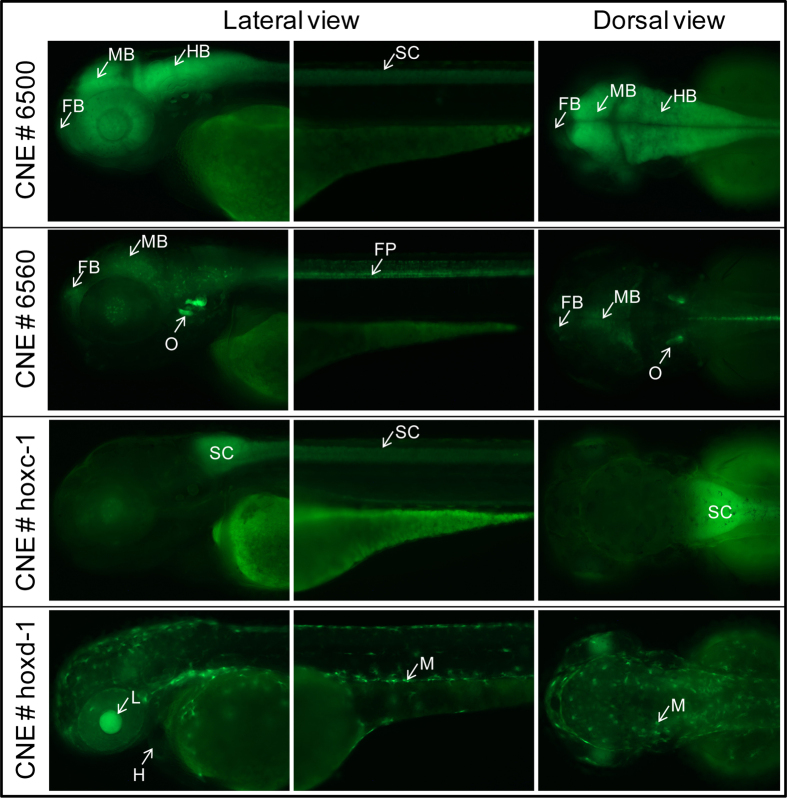
Reporter gene expression pattern driven by zebrafish CNEs that are lost in *H. comes*. Lateral and dorsal views of 72 h post-fertilization F1 transgenic zebrafish embryos. The lost CNEs (#6500, #6560, #hoxc-1 and #hoxd-1) were assayed for their reporter gene expression potential in transgenic zebrafish. FB, forebrain; FP, floor plate; H, heart; HB, hindbrain; L, lens; M, melanocytes; MB, midbrain; O, otic vesicle; SC, spinal cord.

To verify the potential *cis*-regulatory functions of CNEs that were absent in *H. comes* but present in other teleost genomes, we assayed the function of seven selected zebrafish CNEs that were uniquely absent in *H. comes*. Of the seven CNEs assayed in transgenic zebrafish, four CNEs drove reproducible patterns of reporter gene expression in F1 embryos (Extended Data Fig. 7 and Supplementary Table 6.12). Thus, our transgenic assay indicates that some of the CNEs absent in *H. comes* may function as *cis*-regulatory elements in other teleosts. Further studies are required to examine whether the loss of CNEs may have played a role in the evolution of seahorse morphology.

## Summary

Seahorses possess one of the most highly specialized morphologies and reproductive behaviours. We sequenced the genome of the tiger tail seahorse and performed comparative analysis with other teleost fishes. Our genome-wide analysis highlights several aspects that may have contributed to the highly specialized body plan and male pregnancy of seahorses. These include a higher protein and nucleotide evolutionary rate, loss of genes and expansion of gene families, with duplicated genes exhibiting new expression patterns, and loss of a selection of potential *cis*-regulatory elements. It is becoming recognized that evolutionary changes in *cis*-regulatory elements, particularly the loss and gain of enhancers, might play a major part in the evolution of morphological innovations and phenotypic changes across species^21,36,37,40^.

Male pregnancy is a unique developmental feature of seahorses and pipefishes (family Syngnathidae, comprising 57 genera and approximately 300 species). In the seahorse genome, the astacin subfamily of *c6ast* metalloprotease genes has undergone tandem duplications giving rise to six genes. This subfamily of metalloprotease includes the hatching enzyme (also known as choriolysin), HCE-like and HCE2-like enzymes that are responsible for hatching of embryos in fishes^27^. Of the six duplicated genes in seahorse, five are highly expressed in the male brood pouch, suggesting that they may be involved in male pregnancy, possibly through rewiring of their regulatory network. The loss of pelvic fins in seahorse is associated with the evolution of an armour-like covering of its body and gain of an elongated, flexible, substrate-gripping tail. By combining comparative genomics and gene-knockout experiments in zebrafish, we suggest that loss of *tbx4* may have a role in this phenotype in seahorse. The loss of mineralized teeth in seahorse is associated with the fusion of the jaws into a tube-like snout and a small mouth, which is extremely efficient in sucking small food items that are abundant in the benthic environment. In teleosts, P/Q-rich SCPP genes are involved in the mineralization of enameloid, which is the equivalent of enamel in tetrapods^10^. The seahorse genome does not contain any intact P/Q-rich SCPP genes that code for enamel matrix proteins, suggesting that the loss of these genes could have played a part in the loss of its mineralized teeth. Our analyses of the *H. comes* genome sequence and comparative genomics with other teleosts highlighted several genetic changes that may be involved in the evolution of the unique morphology of seahorses.

## Methods

### Genome sequencing and assembly

Genomic DNA of a single male *H. comes* was used to construct eleven libraries including short-insert (170 bp, 500 bp, 800 bp) and mate-paired (2 kb, 5 kb, 10 kb, 20 kb) libraries and sequenced on the Illumina HiSeq 2000 sequencing platform. In total, we obtained around 218 Gb of raw sequence data (Supplementary Table 1.1). The genome was assembled using SOAPdenovo2.04 (ref. 42) with default parameters. No statistical methods were used to predetermine sample size. The experiments were not randomized. The investigators were not blinded to allocation during experiments and outcome assessment.

### RNA sequencing and analysis

In total, 19 RNA-seq libraries were constructed, including two libraries from combined soft tissues (brain, gills, intestine, liver and muscle) from a male and a female *H. comes* (Supplementary Table 2.1); and 17 libraries of five developmental stages of embryos and different stages of brood pouch development such as the juvenile stage, rudimentary stage, pre-pregnancy stage, pregnancy stage, and post pregnancy stage, using RNA from the lined seahorse (*Hippocampus erectus*) (Supplementary Information, section 2). All libraries were prepared using Illumina TruSeq RNA sample preparation kit according to the manufacturer’s instructions (Illumina, San Diego, CA, USA) and sequenced using Illumina HiSeq 2000 platform. The RNA-seq reads were either *de novo* assembled using Trinity^43^ or mapped to the *H. comes* genome using TopHat^44^ with default parameters, and subsequently analysed using in-house Perl scripts. The differential expression of genes at different stages of brood pouch development was determined using the method developed previously^45^. The RNA-seq results were validated using qRT–PCR, with five biological replicates for each stage. All data were expressed as mean ± standard error of mean and were evaluated by one-way ANOVA followed by Tukey’s honestly significant difference test for adjusting *P* values from multiple comparisons. Results were considered to be statistically significant for *P* values < 0.05.

### Genome annotation

Annotation of the *H. comes* genome was carried out using the Ensembl gene annotation pipeline which integrated *ab initio* gene predictions and evidence-based gene models. Briefly, protein sequences of *D. rerio*, *G. aculeatus*, *O. latipes*, *T. rubripes* and *T. nigroviridis* were downloaded from Ensembl (release 75) and mapped to the genome using TblastN^46^ with the parameter “-evalue 1E-5”. Second, high scoring segment pairs (HSPs) from blast were concatenated using Solar (in-house software, version 0.9.6). Third, the concatenated segments were aligned using GeneWise^47^ to refine the gene models. Finally, we filtered the alignments that showed alignment rates less than 50% of the full-length copies and filtered redundant alignments based on the GeneWise score. In addition, *H. comes* transcripts (female_transcript and male_transcript) and *H. erectus* transcripts (Juv_brain, Juv_body, Rud_testis and PreP_pouch) were used to assist in the gene model prediction. We annotated the predicted gene models using Swiss-Prot, TrEMBL, NCBI NR database, and KEGG databases (Supplementary Table 3.4).

### Expansion and contraction of gene families

We used CAFE (version 2.1), a program for analysing gene family expansion and contraction under maximum likelihood framework. The gene family results from TreeFam pipeline and the estimated divergence time between species were used as inputs. We used the parameters “-p 0.01, -r 10000, -s” to search the birth and death parameter (*λ*)of genes, calculated the probability of each gene family with observed sizes using 10,000 Monte Carlo random samplings, and reported birth and death parameters in gene families with probabilities less than 0.01. For the gene family expansion and contraction analysis in *H. comes*, we first filtered out gene families without homology in the SWISS-PROT database to reduce the potential false positive expansions or contractions caused by gene prediction. The families that contained sequences that have multiple functional annotations were also removed (Supplementary Tables 4.1 and 4.2).

### Phylogenetic analysis

We obtained 4,122 one-to-one orthologous genes from the gene family analysis (Supplementary Information, section 4.1). The protein sequences of one-to-one orthologous genes were aligned using MUSCLE^48^ with the default parameters. We then filtered the saturated sites and poorly aligned regions using trimAl (ref. 49) with the parameters “-gt 0.8 –st 0.001 –cons 60”. After trimming the saturated sites and poorly aligned regions in the concatenated alignment, 2,128,000 amino acids were used for the phylogenomic analysis. The trimmed protein alignments were used as a guide to align corresponding coding sequences (CDSs). The aligned protein and the fourfold degenerate sites in the CDSs were each concatenated into a super gene using an in-house Perl script.

The phylogenomic tree was reconstructed using RAxML version 8.1.19 (ref. 50) based on concatenated protein sequences. Specifically, we used the PROTGAMMAAUTO parameter to select the optimal amino acid substitution model, specified spotted gar as the outgroup, and evaluated the robustness of the result using 100 bootstraps. To compare the neutral mutation rate of different species, we also generated a phylogeny based on fourfold degenerate sites. The phylogenomic topology was used as input and the “-f e” option in RAxML was used to optimize the branch lengths of the input tree using the alignment of fourfold degenerate sites under the general time reversible (GTR) model as suggested by ModelGenerator version 0.85 (ref. 51). We calculated the pairwise distances to the outgroup (spotted gar) based on the optimized branch length of the neutral tree using the cophenetic.phylo module in the R-package APE^52^. The Bayesian relaxed-molecular clock (BRMC) method, implemented in the MCMCTree program^53^, was used to estimate the divergence time between different species. The concatenated CDS of one-to-one orthologous genes and the phylogenomics topology were used as inputs. Two calibration time points based on fossil records, *O. latipes*–*T. nigroviridis* (~96.9–150.9 million years ago (Mya)), and *D. rerio*–*G. aculeatus* (~149.85–165.2 Mya) (http://www.fossilrecord.net/dateaclade/index.html), were used as constraints in the MCMCTree estimation. Specifically, we used the correlated molecular clock and REV substitution model in our calculation. The MCMC process was run for 5,000,000 steps and sampled every 5,000 steps. MCMCTree suggested that *H. comes* diverged from the common ancestor of stickleback, Nile tilapia, platyfish, fugu, and medaka approximately 103.8 Mya, which corresponds to the Cretaceous period.

### Analysis of OR genes

We downloaded protein sequences of 1,417 OR gene family members from NCBI and mapped them to *H. comes* genome using Tblastn with “E-value ≤1e-10” and “alignment rate ≥ 0.5”. Solar (in-house software, version 0.9.6) was used to join high-scoring segment pairs (HSPs) between each pair of protein mapping results. We retained alignments with an alignment rate of more than 70% and a mapping identity of more than 40%. Subsequently, the protein sequences were mapped to the genome using GeneWise and extended 280 bp upstream and downstream to define integrated gene models. For phylogenetic analysis, protein sequences were aligned using MUSCLE and a JTT+gamma model was used in a maximum-likelihood analysis using PhyML to construct a phylogenetic tree.

### Evidence for loss of *tbx4* in *H. comes*

The synteny analysis of *tbx2b*-*tbx4*-*brip1* region of *H. comes*, stickleback, fugu and zebrafish using Vista shows that *tbx4* was lost in *H. comes* (Fig. 3). To exclude the scenario that the absence of *tbx4* in the *H. comes* genome sequence is due to an assembly error, we first validated the micro-synteny region of *tbx2b*-*tbx4*-*brip1* region in *H. comes* using a PCR- based genomic walk strategy. Briefly, 28 primer pairs (Supplementary Table 9.1) were designed for overlapping amplicons to ‘walk’ from the end of *tbx2b* to the start of *brip1*. Amplicon size and partial end sequencing of these products did not indicate any anomalies in the assembly of the *H. comes tbx4* ‘ghost locus’.

In addition, we carried out the following analyses: (1) searched the *H. comes* genome (TblastN) using Tbx4 protein from zebrafish and Nile tilapia and were unable to find a *tbx4* gene; (2) searched the *H. comes* genome using only the domain sequence of Tbx4 protein but were unable to find a *tbx4* gene; (3) searched *H. comes* and *H. erectus* transcriptome data for *tbx4* (TblastN) using Tbx4 protein from zebrafish and Nile tilapia but were unable to find any matching transcript; (4) searched *H. comes* and *H. erectus* transcriptome data with the domain sequence as well and did not find any remnant of a *tbx4* gene; and (5) predicted CNEs in the ‘ghost’ *tbx4* locus of *H. comes* using the fugu *tbx4* locus as the reference (base) (Supplementary Fig. 9.3). We used the CNEs present in the other fish genome loci (that were absent in *H. comes*) to search the *H. comes* genome to rule out the possibility that they may be present elsewhere in the genome. We were unable to find any of these CNEs in the *H. comes* genome. Finally, we conducted degenerate PCR experiments to ascertain if the *tbx4* gene is missing in *H. comes*. Using a combination of four forward and two reverse primers (Supplementary Table 9.1), we checked for the presence of *tbx4* in seven species of *Hippocampus* (including *H. comes* and *H. erectus*), five species of pipefish (four from the genus *Syngnathus* and one species of *Corythoichthys*) (all from the family Syngnathidae that lack pelvic fins); ghost pipefish (*Solenostomus*) and the trumpetfish (Aulostomidae) which are closely related to the Syngnathidae but possess pelvic fins; and five other teleost species that possess pelvic fins (Supplementary Figs 9.1 and 9.2).

### Generation of mutant *tbx4* zebrafish

We used a CRISPR–Cas9 strategy to generate a *tbx4* mutant zebrafish line. Two guide RNAs (gRNAs) were designed targeting zebrafish *tbx4* in the 5′ end of the sequence that is upstream of or within the DNA-binding TBOX domain (Supplementary Fig. 9.4). gRNAs were cloned using synthesized oligonucleotides into the pT7gRNA vector as described previously^54^ (oligonucleotide sequences given in Supplementary Table 9.2). gRNAs were synthesized from this vector after linearization with BamH1-HF (NEB R3136T), transcribed using the MEGAscript T7 Transcription Kit (Thermo Fischer Scientific AM1334) and purified using the mirVana miRNA isolation kit (Thermo Fischer Scientific AM1560). Cas9 mRNA was synthesized from the Cs2+Cas9 vector using the mMessage mMachine Sp6 Transcription Kit (Thermo Fischer Scientific AM1340) and purified using the RNA cleanup protocol from the RNAeasy mini kit (Qiagen 74104).

Zebrafish from a wild caught strain were injected at the one-cell stage with ~50 ng gRNA and ~90 ng Cas9 RNA. These F0 fish were raised to maturity and genotyped using fin clipping, DNA isolation and PCR spanning the target site (genotyping primers given in Supplementary Table 9.2). PCR products were analysed for mutations as described previously^54^ using T7 endonuclease (NEB M0302L). Mosaic mutant F0 fish were outcrossed to AB wild-type fish and embryos were batch genotyped for transmission of the mutation using PCR and T7 endonuclease. Mutant PCR products were cloned into the pGEM-T vector (Promega, Madison, WI) and sequenced to identify carrier fish transmitting a frameshift mutation. These carrier fish were crossed again to AB wild type and the resulting F1 fish were raised to maturity. The F1 were genotyped using fin clipping, DNA isolation, PCR, T7 endonuclease to identify heterozygous mutant fish followed by cloning and sequencing of the mutant PCR products to validate presence of the frameshift allele. The CRISPR–Cas9 mutation strategy is schematically shown in Extended Data Fig. 5.

In the F0 mutant *tbx4* fish we observed pelvic fin loss at low frequency. gRNA#1 gave 3/42 fish with either double- or single-sided pelvic fin loss whereas 1/34 had single-sided pelvic fin loss for gRNA#2 (Extended Data Fig. 5). We observed mutant allele transmission for both gRNA#1 and gRNA#2 but failed to identify a deletion leading to a frameshift mutation for gRNA#2 so no stable line was generated for this CRISPR. For gRNA#1 we identified several frameshift mutants, one of which was further analysed. This mutant has a deletion/replacement mutation in which eight nucleotides are replaced by three nucleotides, leading to an effective 5 bp deletion and the introduction of a frameshift mutation (Extended Data Fig. 5). This mutation introduces a downstream STOP codon leading to a severely truncated protein lacking the DNA binding domain (Supplementary Figs 9.4 and 9.5). The mutant line is maintained on an AB wild-type background.

### Loss of CNEs

Using zebrafish as the reference genome, whole-genome alignments of six teleost fishes were generated. The soft-masked genome sequence for zebrafish (Zv9, April 2010) was downloaded from the Ensembl release-75 FTP site. The following soft-masked genome sequences were downloaded from the UCSC Genome Browser: stickleback (gasAcu1, February 2006), fugu (fr3, October 2011), medaka (oryLat2, October 2005), Nile tilapia (oreNil2, February 2012). The *H. comes* genome sequence (hipCom0) was repeat-masked using WindowMasker (from NCBI BLAST+ package v.2.2.28) with additional parameter “-dust true”. About 32% (158.1/501.6 Mb) of the *H. comes* genome was masked using this method.

Only chromosome sequences of zebrafish were aligned while unplaced scaffolds were excluded. The reference (zebrafish) genome was split into 21 Mb sequences with 10-kb overlap, while the percomorph fish genomes (*H. comes*, stickleback, fugu, medaka and Nile tilapia) were split into 10 Mb sequences with no overlap. Pairwise alignments were carried out using Lastz v.1.03.54 (ref. 55) with the following parameters: –strand = both–seed = 12of19–notransition–chain–gapped–gap = 400,30–hspthresh = 3000–gappedthresh = 3000–inner = 2000–masking = 50–ydrop = 9400–scores = HoxD55.q–format = axt. Coordinates of split sequences were restored to genome coordinates using an in-house Perl script. The alignments were reduced to single coverage with respect to the reference genome using UCSC Genome Browser tools ‘axtChain’ and ‘chainNet’. Multiple alignments were generated using Multiz.v11.2/roast.v3 (ref. 56) with the tree topology “(Zv9 (hipCom0 ((fr3 gasAcu1) (oryLat2 oreNil2))))”.

Fourfold degenerate (4D) sites of zebrafish genes (Ensembl release-75) were extracted from the multiple alignments. These 4D sites were used to build a neutral model using PhyloFit in the rphast v.1.5 package^57^ (general reversible “REV” substitution model). PhastCons was then run in rho-estimation mode on each of the zebrafish chromosomal alignments to obtain a conserved model for each chromosome. These conserved models were averaged into one model using PhyloBoot. Subsequently, conserved elements were predicted in the multiple alignments using PhastCons with the following inputs and parameters: the neutral and conserved models, target coverage of input alignments = 0.3 and average length of conserved sequence = 45 bp. To assess the sensitivity of this approach in identifying functional elements, the PhastCons elements were compared against zebrafish protein-coding genes. Eighty per cent of protein-coding exons (197,508/245,556 exons) were overlapped by a conserved element (minimum coverage 10%), indicating that the identification method was fairly sensitive.

A CNE was considered present in a percomorph genome if it showed coverage of at least 30% with a zebrafish CNE in Multiz alignment. To identify CNEs that could have been missed in the Multiz alignments due to rearrangements in the genomes, or due to partitioning of the CNEs among teleost fish duplicate genes, we searched the zebrafish CNEs against the genome of the percomorph using BLASTN (*E* < 1 × 10^−10^; ≥80% identity; ≥30% coverage). Those CNEs that had no significant match in a percomorph genome were considered as missing in that genome. To account for CNEs that might have been missed due to sequencing gaps, we identified gap-free syntenic intervals in zebrafish and the percomorph genomes, and generated a set of CNEs that were missing from these intervals. These CNEs represent a high-confidence set of CNEs missing in the percomorph fishes and thus were used for further analysis. Functional enrichment of genes associated with CNEs was carried out using the GREAT software^58^ with each CNE assigned to the genes with the nearest transcription start site and within 1 Mb in the zebrafish genome, and significantly enriched functional categories identified based on a hypergeometric test of genomic regions (false discovery rate (FDR) *q* value < 0.05). We identified the statistically significant gene ontology biological process terms, molecular function terms and zebrafish phenotype descriptions of the genes that are associated with CNEs.

We also predicted CNEs in the Hox clusters of *H. comes* and other representative teleost fishes using the global alignment program MLAGAN. Orthologous Hox clusters were aligned using MLAGAN with zebrafish as the reference sequence and CNEs were predicted using VISTA.

### Functional assay of CNEs

Seven representative zebrafish CNEs that have been lost in *H. comes* (the largest among the lost CNEs) were assayed for enhancer activity in transgenic zebrafish using GFP as the reporter gene. The CNEs were amplified by PCR using zebrafish genomic DNA as template. The products were cloned into a miniTol2 transposon donor plasmid linked to the mouse *cFos* (McFos) basal promoter and the coding sequence of GFP. Transposase mRNA was generated by transcribing cDNA *in vitro* using the mMESSAGE mMACHINE T7 kit (Ambion; Life Technologies). The CNE-containing McFos-miniTol2 construct and transposase mRNA were co-injected into the yolk of zebrafish embryos at the one to two-cell stage. Each CNE construct was injected into 250–350 embryos and the injections were repeated on two days. The embryos were reared at 28 °C, and GFP was observed at 24, 48 and 72 h post-fertilization (hpf). The survival rate of the embryos post-injection was 70–80%. Consistent GFP expression in at least 20% of F0 embryos was considered as specific expression driven by a CNE. Such embryos were reared to maturity and mated with wild type zebrafish to produce F1 lines. The expression of GFP in F1 embryos was observed under a compound microscope fitted for epifluorescence (Axio imager M2; Carl Zeiss, Germany) and photographed using an attached digital microscope camera (Axiocam; Carl Zeiss, Germany). Pigmentation was inhibited by maintaining zebrafish embryos in 0.003% N-phenylthiourea (Sigma-Aldrich, Sweden) from 8 hpf onwards. Consistent GFP expression observed in at least three lines of F1 fishes was considered as the specific expression driven by a CNE.

All animals were cared for in strict accordance with National Institutes of Health (USA) guidelines. The zebrafish gene knockout protocol was approved by the Institutional Animal Care and Use Committee of Sun Yat-Sen University. The zebrafish transgenic assay protocol was approved by the Institutional Animal Care and Use Committee of Biological Resource Centre, A*STAR, Singapore.

### Data availability statement

The tiger tail seahorse (*H. comes*) whole-genome sequence has been deposited in the DDBJ/EMBL/GenBank database under accession number LVHJ00000000. RNA-seq reads for *H. erectus* and *H. comes* have been deposited in the NCBI Sequence Read Archive under accession numbers SRA392578 and SRA392580, respectively.

## Supplementary information

Supplementary Information This file contains Supplementary Text and Data, Supplementary Tables, Supplementary Figures and Supplementary References. (PDF 6916 kb)

## Data Availability

Sequence Read Archive
SRA392578
SRA392580

## Related audio

Researcher Axel Meyer discusses the weird and beautiful seahorse – now complete with genome.

## PowerPoint slides

PowerPoint slide for Fig. 1
PowerPoint slide for Fig. 2
PowerPoint slide for Fig. 3
PowerPoint slide for Fig. 4

## References

[1] Leysen H (2011). Musculoskeletal structure of the feeding system and implications of snout elongation in *Hippocampus reidi* and *Dunckerocampus dactyliophorus*. J. Fish Biol..

[2] Stölting KN, Wilson AB (2007). Male pregnancy in seahorses and pipefish: beyond the mammalian model. BioEssays.

[3] Wilson AB, Vincent A, Ahnesjö I, Meyer A (2001). Male pregnancy in seahorses and pipefishes (family Syngnathidae): rapid diversification of paternal brood pouch morphology inferred from a molecular phylogeny. J. Hered..

[4] Teske PR, Cherry MI, Matthee CA (2004). The evolutionary history of seahorses (Syngnathidae: *Hippocampus*): molecular data suggest a West Pacific origin and two invasions of the Atlantic Ocean. Mol. Phylogenet. Evol..

[5] Near TJ (2013). Phylogeny and tempo of diversification in the superradiation of spiny-rayed fishes. Proc. Natl Acad. Sci. USA.

[6] Tajima F (1989). Statistical method for testing the neutral mutation hypothesis by DNA polymorphism. Genetics.

[7] Nei, M. & Kumar, S. *Molecular Evolution and Phylogenetics* (Oxford Univ. Press, 2000)

[8] Bailly X (2003). The loss of the hemoglobin H2S-binding function in annelids from sulfide-free habitats reveals molecular adaptation driven by Darwinian positive selection. Proc. Natl Acad. Sci. USA.

[9] MacArthur DG (2007). Loss of *ACTN3* gene function alters mouse muscle metabolism and shows evidence of positive selection in humans. Nature Genet..

[10] Kawasaki K (2011). The SCPP gene family and the complexity of hard tissues in vertebrates. Cells Tissues Organs.

[11] Louchart A, Viriot L (2011). From snout to beak: the loss of teeth in birds. Trends Ecol. Evol..

[12] Meredith RW, Zhang G, Gilbert MT, Jarvis ED, Springer MS (2014). Evidence for a single loss of mineralized teeth in the common avian ancestor. Science.

[13] Deméré TA, McGowen MR, Berta A, Gatesy J (2008). Morphological and molecular evidence for a stepwise evolutionary transition from teeth to baleen in mysticete whales. Syst. Biol..

[14] Zhang G (2014). Comparative genomics reveals insights into avian genome evolution and adaptation. Science.

[15] Yamanoue Y, Setiamarga DH, Matsuura K (2010). Pelvic fins in teleosts: structure, function and evolution. J. Fish Biol..

[16] Kuiter, R. H. *Seahorses and their Relatives* (Aquatic Photographics, 2009)

[17] Harris JE (1938). The role of the fins in the equilibrium of the swimming fish. II. The role of the pelvic fins. J. Exp. Biol..

[18] Gosline WA (1980). The evolution of some structural systems with reference to the interrelationships of modern lower teleostean fish groups. Jpn. J. Ichthyol..

[19] Standen EM (2008). Pelvic fin locomotor function in fishes: three-dimensional kinematics in rainbow trout (*Oncorhynchus mykiss*). J. Exp. Biol..

[20] Tanaka M (2005). Developmental genetic basis for the evolution of pelvic fin loss in the pufferfish *Takifugu rubripes*. Dev. Biol..

[21] Chan YF (2010). Adaptive evolution of pelvic reduction in sticklebacks by recurrent deletion of a *Pitx1* enhancer. Science.

[22] Naiche LA, Papaioannou VE (2003). Loss of Tbx4 blocks hindlimb development and affects vascularization and fusion of the allantois. Development.

[23] Rodriguez-Esteban C (1999). The T-box genes *Tbx4* and *Tbx5* regulate limb outgrowth and identity. Nature.

[24] Tamura K, Yonei-Tamura S, Izpisúa Belmonte JC (1999). Differential expression of *Tbx4* and *Tbx5* in zebrafish fin buds. Mech. Dev..

[25] Arora R, Metzger RJ, Papaioannou VE (2012). Multiple roles and interactions of Tbx4 and Tbx5 in development of the respiratory system. PLoS Genet..

[26] Don EK (2016). Genetic basis of hindlimb loss in a naturally occurring vertebrate model. Biol. Open.

[27] Kawaguchi M (2006). Evolution of teleostean hatching enzyme genes and their paralogous genes. Dev. Genes Evol..

[28] Harlin-Cognato A, Hoffman EA, Jones AG (2006). Gene cooption without duplication during the evolution of a male-pregnancy gene in pipefish. Proc. Natl Acad. Sci. USA.

[29] Whittington CM, Griffith OW, Qi W, Thompson MB, Wilson AB (2015). Seahorse brood pouch transcriptome reveals common genes associated with vertebrate pregnancy. Mol. Biol. Evol..

[30] Kawaguchi M, Tomita K, Sano K, Kaneko T (2015). Molecular events in adaptive evolution of the hatching strategy of ovoviviparous fishes. J. Exp. Zool. B Mol. Dev. Evol..

[31] Bejerano G (2004). Ultraconserved elements in the human genome. Science.

[32] Lindblad-Toh K (2011). A high-resolution map of human evolutionary constraint using 29 mammals. Nature.

[33] Venkatesh B (2006). Ancient noncoding elements conserved in the human genome. Science.

[34] Navratilova P (2009). Systematic human/zebrafish comparative identification of *cis*-regulatory activity around vertebrate developmental transcription factor genes. Dev. Biol..

[35] Visel A (2008). Ultraconservation identifies a small subset of extremely constrained developmental enhancers. Nature Genet..

[36] Attanasio C (2013). Fine tuning of craniofacial morphology by distant-acting enhancers. Science.

[37] McLean CY (2011). Human-specific loss of regulatory DNA and the evolution of human-specific traits. Nature.

[38] Sabherwal N (2007). Long-range conserved non-coding SHOX sequences regulate expression in developing chicken limb and are associated with short stature phenotypes in human patients. Hum. Mol. Genet..

[39] Shears DJ (1998). Mutation and deletion of the pseudoautosomal gene *SHOX* cause Leri–Weill dyschondrosteosis. Nature Genet..

[40] Indjeian VB (2016). Evolving new skeletal traits by *cis*-regulatory changes in bone morphogenetic proteins. Cell.

[41] Lin Q, Lin J, Zhang D (2008). Breeding and juvenile culture of the lined seahorse, *Hippocampus erectus* Perry, 1810. Aquaculture.

[42] Luo R (2012). SOAPdenovo2: an empirically improved memory-efficient short-read *de novo* assembler. Gigascience.

[43] Grabherr MG (2011). Full-length transcriptome assembly from RNA-seq data without a reference genome. Nature Biotechnol..

[44] Trapnell C, Pachter L, Salzberg SL (2009). TopHat: discovering splice junctions with RNA-seq. Bioinformatics.

[45] Yu X, Lin J, Zack DJ, Qian J (2006). Computational analysis of tissue-specific combinatorial gene regulation: predicting interaction between transcription factors in human tissues. Nucleic Acids Res..

[46] Kent WJ (2002). BLAT—the BLAST-like alignment tool. Genome Res..

[47] Birney E, Clamp M, Durbin R (2004). GeneWise and Genomewise. Genome Res..

[48] Edgar RC (2004). MUSCLE: multiple sequence alignment with high accuracy and high throughput. Nucleic Acids Res..

[49] Capella-Gutiérrez S, Silla-Martínez JM, Gabaldón T (2009). trimAl: a tool for automated alignment trimming in large-scale phylogenetic analyses. Bioinformatics.

[50] Stamatakis A (2006). RAxML-VI-HPC: maximum likelihood-based phylogenetic analyses with thousands of taxa and mixed models. Bioinformatics.

[51] Stamatakis A, Hoover P, Rougemont J (2008). A rapid bootstrap algorithm for the RAxML Web servers. Syst. Biol..

[52] Paradis E, Claude J, Strimmer K (2004). APE: Analyses of phylogenetics and evolution in R language. Bioinformatics.

[53] Yang Z (2007). PAML 4: phylogenetic analysis by maximum likelihood. Mol. Biol. Evol..

[54] Jao LE, Wente SR, Chen W (2013). Efficient multiplex biallelic zebrafish genome editing using a CRISPR nuclease system. Proc. Natl Acad. Sci. USA.

[55] Harris, R. S. *Improved Pairwise Alignment of Genomic DNA*. PhD thesis, Pennsylvania State Univ. (2007)

[56] Blanchette M (2004). Aligning multiple genomic sequences with the threaded blockset aligner. Genome Res..

[57] Hubisz MJ, Pollard KS, Siepel A (2011). PHAST and RPHAST: phylogenetic analysis with space/time models. Brief. Bioinform..

[58] McLean CY (2010). GREAT improves functional interpretation of *cis*-regulatory regions. Nature Biotechnol..

[59] Bian C (2016). The Asian arowana (*Scleropages formosus*) genome provides new insights into the evolution of an early lineage of teleosts. Sci. Rep..

[60] Kawasaki K, Amemiya CT (2014). SCPP genes in the coelacanth: tissue mineralization genes shared by sarcopterygians. J. Exp. Zool. B Mol. Dev. Evol..

[61] Venkatesh B (2014). Elephant shark genome provides unique insights into gnathostome evolution. Nature.

