# Supplementary material for: The seahorse genome and the evolution of its specialized morphology
Source: Nature. 2016 Dec 14;540(7633):395–9. doi: 10.1038/nature20595 (PMC8127814; doi:10.1038/nature20595)
Supplement: Supplementary Information — This file contains Supplementary Text and Data, Supplementary Tables, Supplementary Figures and Supplementary References. (PDF 6916 kb) [file 41586_2016_BFnature20595_MOESM68_ESM.pdf]

doi:10.1038/nature20595

Table of contents

1. Genome sequencing and assembly 2
1.1. DNA sample preparation and sequencing 2
1.2. Quality control of raw sequencing reads 2
1.3. Estimate the Genome Size using K-mer spectrum 3
1.4. Genome assembly 4
1.5. Quality control of *H. comes* genome assembly 4
1.6. GC-content of *H. comes* genome 6
2. RNA sequencing 7
3. Genome analysis 11
3.1. Transposable element analysis 11
3.2. Gene prediction and annotation 13
3.3. Differential expression analysis 16
4. Gene family evolution 23
4.1. Gene family analysis 23
4.2 Phylogenetic tree construction and divergence time estimate 24
4.3 Expansion and contraction of gene families 24
4.4 Rate of molecular evolution 25
5.Detection of positively selected genes 32
6.Loss of conserved noncoding elements (CNEs) 40
7.Hox gene evolution 59
8.OR (olfactory receptors) genes in seahorse 63
9.Loss of tbx4 in seahorse and generation of a *tbx4* mutant zebrafish line 64
10.Description and analyses of specific gene families 76
10.1 Secretory calcium-binding phosphoprotein (SCPP) gene family 76
10.2 The evolution of astacin metalloproteinase gene family 78
11.References 85

1. Genome sequencing and assembly

1.1. DNA sample preparation and sequencing

Supplementary Table 1.1 Statistics of Sequencing Data

Insert size (bp)	Raw data				Filter data			
	Reads Length	Total Data(Mb)	Sequence Depth (×)	Physical Depth (×)	Reads Length	Total Data(Mb)	Sequence Depth(×)	Physical Depth(×)
**170**	100	19760.94	28.72	24.41	100	15899.28	23.11	19.64
**250**	150	63338.12	92.06	76.72	150	52679.57	76.57	63.81
**500**	100	19077.93	27.73	69.32	100	14044.48	20.41	51.03
**800**	100	17297.92	25.14	100.57	100_72*	11205.84	16.29	75.76
**2K**	49	9595.81	13.95	284.64	49	7298.75	10.61	216.50
**5K**	49	23958.49	34.82	1776.70	49	11399.10	16.57	845.33
**10K**	49	24251.23	35.25	3596.82	49	12311.32	17.89	1825.96
**20K**	49	41425.18	60.21	12287.96	49	7292.73	10.60	2163.24
**Total**	---	218705.64	317.89	18217.16	---	132131.06	192.05	5261.27

* The last 28 bp were trimmed due to sequencing error.

1.2. Quality control of raw sequencing reads

To prepare high quality data for *de novo* genome assembly of seahorse genome, the raw sequencing data were filtered using a combined strategy.

1) Filtered reads in which N constitutes more than 2% (for the short-insert libraries), 5% or 10% (for the mate-paired libraries) of read length or polyA structure reads.
2) Filtered low quality reads. Reads of short-insert libraries that have 40% bases with quality scores ≤7; reads of mate-paired libraries that have more than 30% or 40% bases with quality scores ≤7.
3) Filtered reads with adapter contamination. Reads with more than 10 bp aligned to the adapter sequences (allowing less than or equal to 3 bp mismatches) were filtered.
4) Filtered short insert-size libraries (250 bp, 500 bp, 800 bp insertion size) in which forward and reverse reads overlapped >= 10 bp allowing 10% mismatches and Read1 and Read2 are both ends of one paired end reads.
5) Filtered PCR duplicates.
6) The raw reads were also corrected based on K-mer spectrum.

Finally, 132.131 Gb (around 192.05× coverage of seahorse genome) reads were achieved.

1.3. Estimate the Genome Size using K-mer spectrum

The genome size of seahorse was estimated based on K-mer spectrum[1]. Given the K-mer frequency obeysa Poisson distribution, when the coverage is sufficient, genome size can be estimated by:

$$\text{Genome Size} = K_{num}/K_{depth}$$

Where K_{num} is the number of K-mers, and K_{depth} is the expected depth of K-mers. In this study, the K_{num}, is 23,386,207,821 based on 17 mers and K_{depth} is 34 (Supplementary Figure 1.1). Therefore, we estimated the seahorse genome size is around 688 Mb.

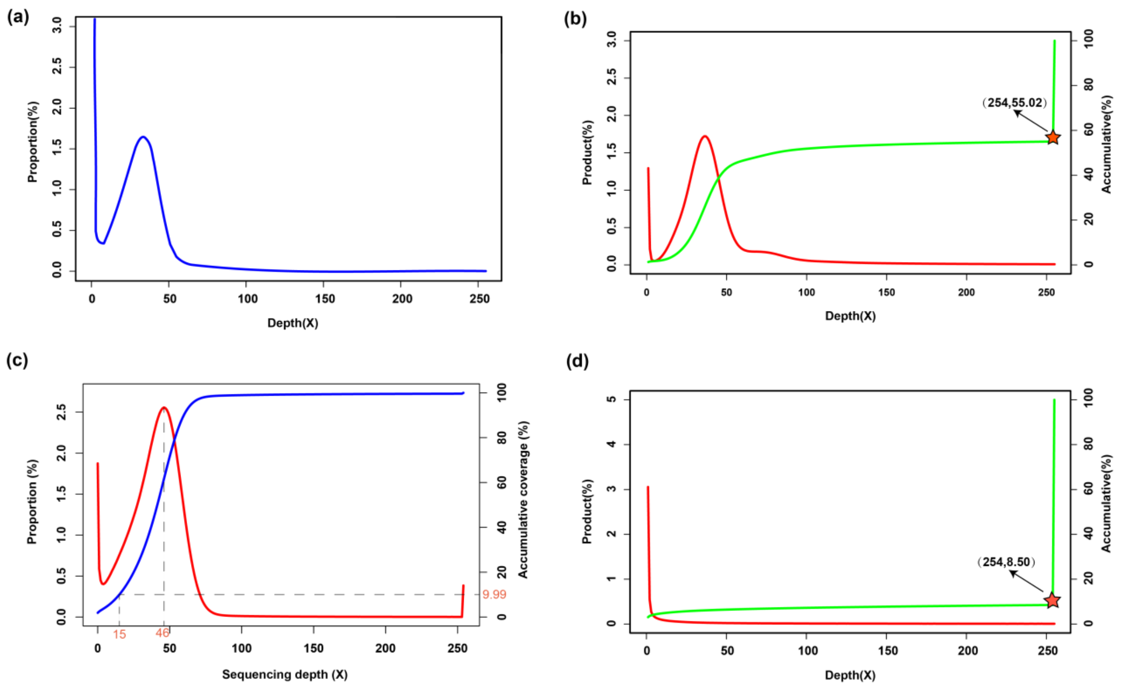

Supplementary Figure 1.1 Genome size and sequence depth estimation based on K-mer spectrum. The K-mer spectrum was constructed based on a 17 mer.
(a) K-mer spectrum of raw reads. The x-axis is the depth of K-mer; the y-axis is the proportion of K-mers. The peak of the distribution is 34. (b) The accumulative of depth coverage of K-mers. The x-axis is the depth of coverage, the red curve indicates the percentage of depth coverage (the left y-axis) and the green curve indicates the accumulated depth of coverage of K-mers. The figure shows that more than 44.98 % of the K-mer is in extreme high frequency with depth>= 255, suggesting a high proportion of repeat element in the seahorse genome. (c) The depth coverage of the seahorse genome assembly. The cleaned reads from 170 bp, 500 bp, 800 bp libraries were mapped to the genome assembly using bwa. The X-axis is the depth of coverage; red

curve indicates the percentage of depth of coveage (left Y-axis) and blue curve is the accumulated percentage of depth of coverage (right Y-axis). From the figure, we can see the average depth coverage is 46 and 90% of the base's sequencing depth larger than 15. This indicates the assembly result has a high sequencing depth. (d) The K-mer spectrum of unmapped reads. We conducted the K-mer spectrum analysis using around 49.6 Million reads (~5× of the estimated genome size) that could not be mapped to the seahorse genome uniquely. The X-axis is depth coverage of K-mers, red curve indicates the percentage of depth of coverage (left Y-axis) and blue curve is the accumulated percentage of depth of coverage (right Y-axis). We found that more than 91% of the K-mers from the unmapped reads with high depth of coverage (>=255).

1.4. Genome assembly

The seahorse genome was assembled by SOAPdenovo2.04[2] using the default parameters[1]. Specifically, we first constructed the contigs using the filtered reads of paired-end libraries (170bp, 500bp, 800bp); the mate-paired reads were used to bridge the contigs; finally the assembly gaps were filled using the reads of paired-end libraries by GapCloser1.10. The final seahorse assembly is around 501 Mb (Supplementary Table 1.2).

Supplementary Table 1.2 Summary of seahorse genome assembly.

	Scaffold		Contig	
	Size (bp)	Number	Size (bp)	Number
N50	1,870,882	65	34,668	3,777
N60	1,431,692	96	26,559	5,317
N70	956,739	138	19,214	7,389
N80	670,863	201	12,586	10,378
N90	307,480	309	5,426	15,809
Longest	9,810,584	---	249,437	---
Total Size	501,592,652	---	467,148,839	---
Total Number (>=100bp)	---	94,070	---	119,536
Total Number (>=2kb)	---	1,142	---	21,117

1.5. Quality control of *H. comes* genome assembly

The quality of the assembled seahorse genome was assessed by CEGMA[3], depth of coverage, and assembled transcriptome data. CEGMA evaluates completeness of genome assembly using a set of genes that are widely conserved in eukaryotic genomes. CEGMA analysis showed that 243 out of 248 genes are complete in the seahorse

genome assembly.

We estimated the depth of coverage of seahorse genome using the reads of paired-end libraries. In total, around 383 million reads were mapped to the seahorse genome assembly using bwa[4] version 0.5.9-r16 with the default parameters; and the depth of coverage was estimated using Soapcoverage (version 2.27) (http://soap.genomics.org.cn/). Soapcoverage suggested that around 334M (87.1%) reads, which covered 98.1% of the assembly, were mapped uniquely on the genome (Supplementary Table 1.3 and Supplementary Figure 1.1c). Based on the K-mer spectrum, we found that the reads with low mapping score were mainly originated from the repetitive regions in the genome (Supplementary Figure 1.1d).

The completeness of coding region in the seahorse genome was evaluated using the assembled RNA-seq transcripts. Specifically, the RNA-seq reads of *H. comes* were *de novo* assembled using Trinity[5] and mapped to the *H. comes* genome assembly using Blat[6]. The results showed that out of 77,040 transcripts, 76,757 transcripts could be mapped to the assembly (Supplementary Table 1.4).

Supplementary Table 1.3 Read mapping results for *Hippocampus comes* genome

Species	Genome size(bp)	Effective size (bp)	Covered base (bp)	Total reads (M)	Mapped reads (M)	Reads map (%)	Coverage (%)
Hippocampus comes	501,592,652	467,151,988	458,422,072	384.30	334.74	87.1	98.1

Genome size: the total scaffold length; **Effective size:** the total contig length; **Covered bases:** the contig length were covered by reads; **Total reads:** the number of reads; **Mapped reads:** the number of mapped reads; **Reads map:** percentage of mapped reads; **Coverage:** percentage of Covered bases/Effective size

doi:10.1038/nature20595

Supplementary Table 1.4 Assessment of the completeness of coding region using transcriptome data in the seahorse genome.

Dataset	Number	Total Length (bp)	Base covered by Assembly (%)	Sequence covered by Assembly (%)	With >90% sequence in one Scaffold		With >50% Sequence in one Scaffold	
					Number	Percent (%)	Number	Percent (%)
**All**	77,040	59,709,227	98.48	99.80	72,510	94.12	76,757	99.63
**>200bp**	77,040	59,709,227	98.48	99.80	72,510	94.12	76,757	99.63
**>500bp**	35,208	46,589,365	98.40	99.95	32,408	92.05	35,105	99.71
**>1000bp**	17,796	34,371,389	98.28	99.97	16,026	90.05	17,730	99.63

1.6. GC-content of *H. comes* genome

The GC content of the seahorse genome was estimated using a sliding window approach. Briefly, a 500 bp (250 bp stepwise) sliding window was employed to scan along the genome and calculate the GC content. We found that the average GC content of seahorse genome is about 43%, which is within range of the contents in other teleost genomes (Supplementary Figure 1.2).

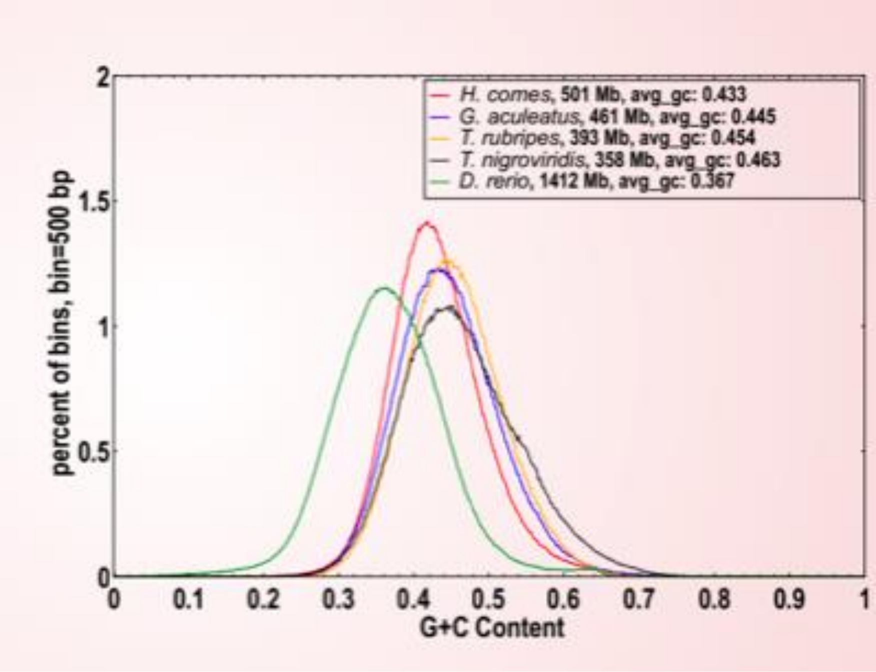

Supplementary Figure 1.2 GC content in teleost genomes. The x-axis is GC content and the y-axis is the proportion of the windows number divided by the total windows. The GC content was calculated using a 500 bp sliding window (250 bp stepwise).The figure shows thatthe GC content in the seahorse genome is around 43%.

2. RNA sequencing

In total, 19 RNA-seq libraries were constructed in this study, including two libraries of combined soft-tissues (brain, gills, intestine, liver, and muscle) from a male and a female *H. comes* (Supplementary Table 2.1) and 17 libraries of five developmental stages of embryos and different stages of brood pouch development such as juvenile stage, rudimentary stage, pre-pregnancy stage, pregnancy stage, and post pregnancy stage, using RNAs from lined seahorse (*Hippocampus erectus*).
We defined the development periods of seahorse in this study as:

- The juvenile stage (Juv): male seahorse with a swell of brood pouch (3 months post birth).
- The rudimentary stage (Rud): male seahorse with the rudimentary brood pouch (5 months post birth).
- The pre-pregnancy stage (PreP): male seahorse with mature brood pouch (7 months post birth).
- The pregnancy stage (P): pregnant seahorse that carries10 day embryos in the brood pouch (8 months post birth).
- The post-pregnancy stage (PostP): male seahorse that has released their offspring for 10 days, but is not pregnant again.

The libraries (insertion size 200 bp) were sequenced 90 bp at each end using Illumina Hiseq 2000 platform. On average we achieved at 46 million reads per library (Supplementary Table 2.2). The RNA-seq reads were mapped to the seahorse genome using Tophat[7] with default parameters, and subsequently analyzed using in-house Perl scripts.

Supplementary Table 2.1 Transcriptome (*H. comes*) sequencing data statistics.

Sample	Total reads	Total Map	Total Map Rate	Unip Map	Uniq MapRate
**Female**	40898690	37596130	91.93	23343832	57.08
**Male**	36911215	33462284	90.66	20261470	54.90

Supplementary Table 2.2 Transcriptome (*H. erectus*) sequencing data statistics.

Sample	TotalReads	TotalMap	TotalMapRate	UnipMap	UniqMapRate

**Juv_brain**	46899916	22525693	48.03	15309074	32.64
**Juv_haslet**	47196974	26944066	57.09	14875733	31.52
**Juv_body***	47040770	29655932	63.04	17483683	37.17
**Rud_brain**	46694266	22087677	47.30	15025424	32.18
**Rud_testis**	47692406	28168264	59.06	16593891	34.79
**Rud_pouch**	47058460	28783113	61.16	18279269	38.84
**PreP_brain**	47238606	23941010	50.68	15878562	33.61
**PreP_testis**	49013880	25548191	52.12	16000354	32.64
**PreP_pouch**	47510960	26423550	55.62	15955969	33.58
**P_brain**	48913948	27923325	57.09	17599469	35.98
**P_testis**	46977462	28621017	60.92	16658010	35.46
**P_pouch**	46843472	28709020	61.29	16404319	35.02
**PostP_brain**	46927064	23398448	49.86	15336865	32.68
**PostP_testis**	47019804	25720942	54.70	15821227	33.65
**PostP_pouch**	47066354	27468918	58.36	16802317	35.70

*The brood pouch of male seahorse was at the juvenile period (only a swell); so the tissue for transcriptome sequencing was the mixture of trunk and brood pouch.

Since brood pouch is not fully developed at the juvenile stage, we used a mix of trunk and brood pouch instead.

Supplementary Table 2.3 Transcriptome sequencing data statistics of *H. erectus* embryos

Sample	TotalReads	TotalMap	TotalMapRate	UnipMap	UniqMapRate
1-dpf Embryos	44906940	21040127	46.85	11918296	26.54
3-dpf Embryos	49040172	32147158	65.55	18117665	36.94
10-dpf Embryos	43469846	27518854	63.31	15973425	36.75
1-day-old juvenile*	46845740	29620332	63.23	15961025	34.07

* young seahorse one day after hatching from the brood pouch. dpf, day(s) post fertilization.

A heatmap depicting the expression profile of astacin family genes in the brain, testis and brood pouch of *Hippocampus erectus* at four developmental stages is shown in Supplementary Figure. 2.1a. Out of the six *pastn* genes identified in the genome assembly of *H. comes*, we could identify orthologues for five genes (*pastn1*, *pastn2*, *pastn3*, *pastn5* and *pastn6*) in the RNA-seq data of *Hippocampus erectus* (Supplementary Figure 2.1b).

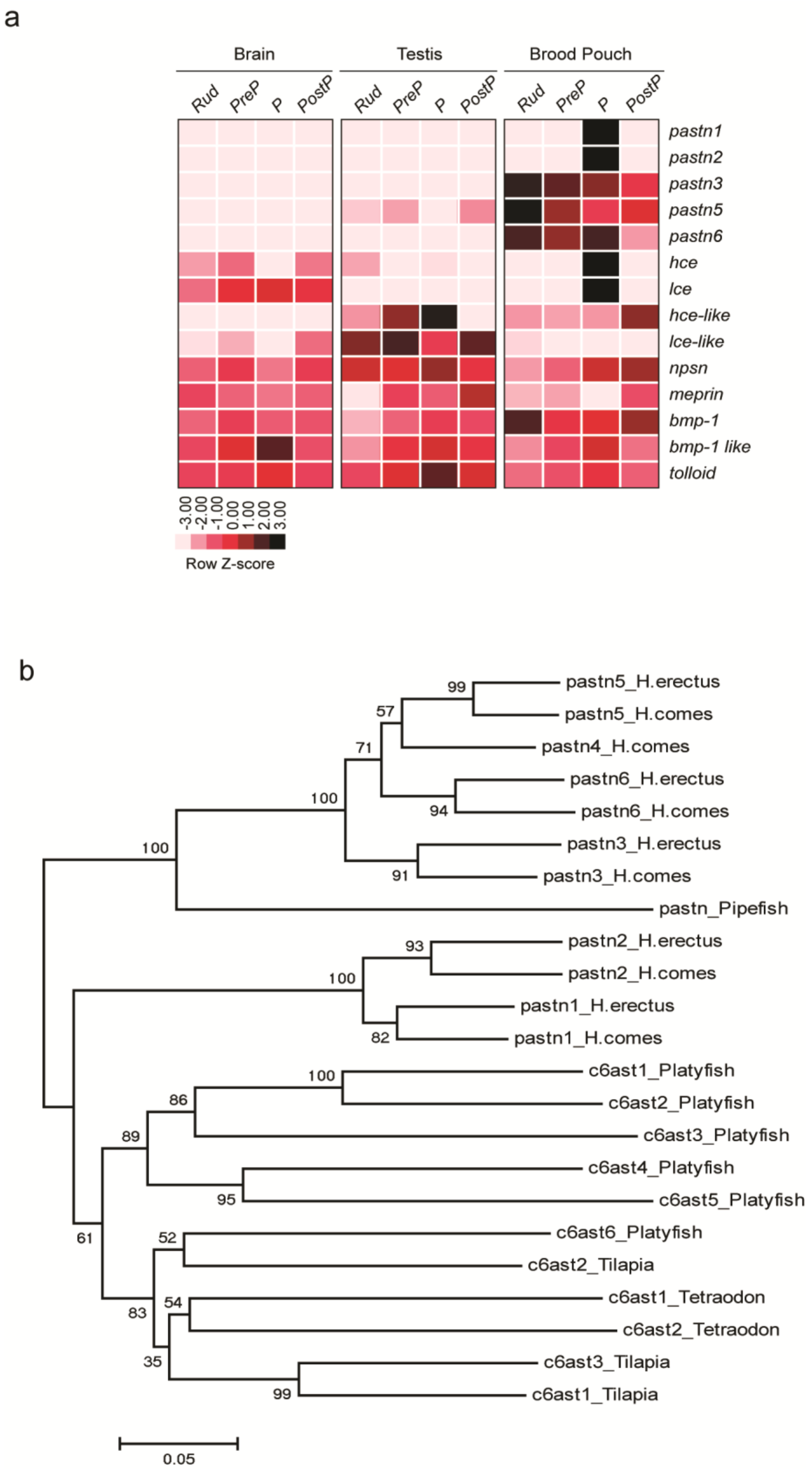

Supplementary Figure 2 Expression profile of astacin genes in the brain, testis and brood pouch at different developmental stages of the Lined seahorse (*Hippocampus erectus*) and their relationships to *H. comes pastn* genes. **(a)** The heat map based on RNA-seq generated for *Hippocampus erectus*. Rud, rudimentary brood pouch (5 months-old immature fish); PreP, pre-pregnant brood pouch (7 months-old); P, pregnant

brood pouch (8 months-old) and PostP, post parturition (10 days after parturition, but not pregnant again). **(b)** Phylogenetic tree (Neighbor-joining) of protein sequences of *pastn* and related genes in *Hippocampus comes* (*H. comes*), *Hippocampus erectus* (*H. erectus*) and other fishes (also see Figure 4 in the main text).

3. Genome analysis

3.1. Transposable element analysis

We constructed a transposable element (TE) library of *H. comes* genome using a combination of homology-based and *de novo* approaches.

1). Tandem repeats were identified using Tandem Repeats Finder (version 4.04, http://tandem.bu.edu/trf/trf.html)[8]. (Supplementary Table 3.1).

2). RepeatMasker(version 3.3.0, http://www.repeatmasker.org/) and RepeatProteinMask were employed to identify TE based on homologous search against a library of Repbase[9] (Release 16.03) using the parameters“-nolow -no_is -norna -parallel 1” and “-noLowSimple –pvalue 1e-4”

3). Ab initio TE library was constructed by RepeatModeler version 1.08 (http://www.repeatmasker.org/RepeatModeler.html) using the default parameters. RepeatModeler identifies repeat elements by integrating two repeat finding programs RECON[10] and RepeatScout[11]. Using the repeat library constructed by RepeatModeler, we estimated the repeat content of seahorse genome using RepeatMasker version 4.0.5 with the sensitive mode (-s). The transposable element (TE) expansion history was constructed by first recalculating the divergence of the identified TE copies in the genome with the corresponding consensus sequence in the TE library using Kimura distance[12] and then estimated the percentage of TE in the genome at difference divergence levels.

Around 24.8% (124.50 Mb) of the tiger tail seahorse (*H. comes*) genome comprises of TEs (Supplementary Table 3.1). Although its genome is relatively small in comparison to other teleost fishes such as zebrafish, tilapia and mudskipper (Supplementary Table 3.2), the TE repertoire in the seahorse genome, estimated by the number of TE super-families, shows a wide range of diversity, which is a common feature in ray-finned fish genomes[13]. The class II DNA transposon, covered around 9% (45 Mb) of the genome, is the most abundant class (Supplementary Table 3.1).

doi:10.1038/nature20595

Supplementary Table 3.1 TE Content in the *H. comes* genome.

Class	Transposable element families	Percentage	Total length (Mb)
Class I Retrotransposon	Ngaro	1.02	5.10
	Pao	0.02	0.08
	Gypsy	0.16	0.79
	ERV1	0.04	0.18
	ERVK	0.03	0.15
	L1	0.01	0.04
	RTE	0.15	0.74
	Rex-Babar	0.45	2.25
	L2	0.98	4.91
	Jockey-I	0.03	0.13
	Dong-R4	0.06	0.31
	R2	0.01	0.05
	Penelope	0.12	0.62
	SINE	0.21	1.05
	Deu	0.01	0.04
	tRNA-V	1.70	8.53
	MIR	0.06	0.28
Class II DNA transposons	CMC	0.12	0.60
	Ginger	0.07	0.36
	Harbinger	0.07	0.38
	hAT	4.46	22.38
	Maverick	0.01	0.03
	DNA	0.48	2.43
	MULE	0.14	0.68
	PiggyBac	0.03	0.16
	Sola	0.15	0.77
	TcMar	3.60	18.06
	RCHelitron	0.00	0.02
	Unknown	10.64	53.38
Total		24.82	124.50

Supplementary Table 3.2 Transposable element content in selected teleost fish genomes.

Species	Genome size (Gb)	TE content (%)	Reference
Danio rerio	1.40	50	14
Takifugu rubripes	0.33	2.7	15
Oryzias latipes	0.70	16	16
Gadus morhua	0.75	18	17
Oreochromis niloticus	1.10	18.75	18
Xiphophorus maculatus	0.67	4.8	19
Gasterosteus aculeatus	0.46	25	20
Scartelaos histophorus	0.80	41.25	21

3.2. Gene prediction and annotation

An evidence-based gene prediction approach was used to create gene models for seahorse.

RNA-seq data provide a good supplement for gene model prediction, as a large number of gene models based on homologous prediction did not have intact open reading frames (ORFs). We used *H. comes* transcripts (female_transcript and male_transcript) and *Hippocampus erectus* transcripts (Juv_brain, Juv_body, Rud_testis and PreP_pouch) to assist the gene model prediction. The RNA-seq reads were assembled into transcripts using the following steps.

1. The raw reads were placed on the genome using TopHat[7] with the default parameters and assembled into transcripts using Cufflinks[22].
2. The assembled transcripts were used to refine the gene models of homology-based approach that the overlapping gene models of RNA-seq and homology-based approach were merged.
3. We also predicted gene models using the transcripts that didn't overlap with the homology-based models. A fifth-order Markov model was trained using 1,000 intact gene models of the homology-based approach and was used to predicted ORF of RNA transcripts.

Finally, 23,458 gene models were produced (Supplementary Table 3.3).

Supplementary Table 3.3 Summary of predicted gene models in the seahorse genome. The final gene set of seahorse genome was created by merging the gene models based

on homologous predictions and on the RNA-seq data of seahorse.

Geneset		GN	AGL	ACL	AEN	AEL	AIL
Homolog	*D. rerio*	24,829	7265.28	1242.95	7.14	174.08	980.78
	G. aculeatus	23,227	7788.12	1294.06	7.75	166.94	961.88
	O. latipes	41,541	4191.12	779.02	4.68	166.29	926.02
	T. rubripes	20,736	8696.64	1436.91	8.51	168.86	966.76
	T. nigroviridis	19,963	8478.18	1405.11	8.49	165.5	944.35
Transcripts	Transcripts_v1	21,996	10771.84	1350.76	8.39	160.95	1062.12
	Transcripts_v2	16,540	12106.54	1558.14	9.19	169.5	982.25
Final gene set		23,458	9874.1	1419.26	8.33	170.31	1040.17

GN: Gene Number; AGL: Average Gene Length; ACL: Average CDS Length; AEN: Average Exon Number; AEL: Average Exon Length; AIL: Average Intron Length; Transcripts_v1:the longest ORF from 6 way of phase; Transcripts_v2: the predicted ORF based on a fifth-order Markov model.

We annotated the predicted gene models using Swiss-Prot[23], TrEMBL[23], NCBI NR database, and KEGG[24] databases (Supplementary Table 3.4) using the following steps:

- The gene symbol and pathway were assigned based on the best blast hit against Swiss-Prot and KEGG databases.
- The motifs and domains in protein sequences were annotated using InterProScan[25] by searching publicly available databases, including Pfam, PRINTS, PANTHER, PROSITE, ProDom, and SMART.
- Gene Ontology[26] terms were assigned using by Blast2GO[27,28].

Supplementary Table 3.4 The number of gene model that can be annotated using different databases.

	Number	Percent (%)
InterPro	16,648	70.97
Nr	20,559	87.64

KEGG	14,535	61.96
Swissprot	18,039	76.90
TrEMBL	21,718	92.58
Annotated	22,245	94.83
Un-annotated	1,213	5.17
Total	23,458	100

3.3. Differential expression analysis

3.3.1 Differential expression genes in seahorse (*Hippocampus erectus*) brood pouch

To have a better understanding of the evolution and development of brood pouch in seahorse, we systematically examined the dynamic of gene expression at four developmental stages, including rudimentary (Rud), pre-pregnancy (PreP), pregnancy (P), and post-pregnancy (PostP) stages. Besides, the RNA sample from the mixture of trunk and brood pouch at the juvenile period (Juv_body) was also included into the analysis as a stage before the development of brood pouch. The stage differential expression genes were detected a method reported by Yu et al[29]. Briefly, the expected number of reads for each gene at stage i is equal to:

$$f_i = E(g)p_i$$

where $E(g)$ is the total number of the RNA-seq reads at different stages, p_i is the proportion of the RNA-seq reads at stage i to the total number of reads.

We calculated the expect enrichment (EE) for each gene (g) at stage i as $EE_i(g)=e_i(g)/f_i(g)$, which is ratio of the observed and expected number of reads for gene g. A larger EE score indicates gene g is differentially expressed at stage i. The statistical significance of EE score was calculated using

$$p_i(g) = \sum_{x=e_i(g)}^{E(g)} \binom{E(g)}{x} p_i^x (1 - p_i)^{E(g)-x}$$

where x is the number of reads at stage x, E(g) is the total number of reads of all stages. In this study, we defined differential expression genes at stage i are with $EEi(g) > 3$ and p-value < 0.01. We also filtered out genes that were lowly expressed (RPKM<2) at all five stages.

In total, we identified 927, 105, 46, 119 and 31 tissue-specific differential expression genes in Juv_pouch, Rud_pouch, PreP_pouch, P_pouch and PostP_pouch respectively (Supplementary Figure 3.1)

GO ID	GO term	GO Class	Q-value
GO:0030016	myofibril	CC	6.59E-39
GO:0043292	contractile fiber	CC	1.55E-37
GO:0044449	contractile fiber part	CC	5.75E-37
GO:0030017	sarcomere	CC	2.50E-36
GO:0006941	striated muscle contraction	BP	2.37E-26
GO:0005865	striated muscle thin filament	CC	5.18E-25
GO:0003012	muscle system process	BP	1.09E-22
GO:0006936	muscle contraction	BP	1.62E-21
GO:0015629	actin cytoskeleton	CC	2.44E-19
GO:0005861	troponin complex	CC	4.48E-19
GO:0030239	myofibril assembly	BP	7.50E-18
GO:0031032	actomyosin structure organization	BP	3.94E-17
GO:0003015	heart process	BP	7.04E-16
GO:0031674	I band	CC	5.31E-15
GO:0060047	heart contraction	BP	1.51E-14

GO ID	GO Term	GO Class	Q-value
GO:0004866	endopeptidase inhibitor activity	MF	0.02265
GO:0061135	endopeptidase regulator activity	MF	0.02792
GO:0030414	peptidase inhibitor activity	MF	0.03075
GO:0052689	carboxylic ester hydrolase activity	MF	0.03472

GO ID	GO term	GO Class	Q-value
GO:0005527	macrolide binding	MF	0.00283
GO:0005528	FK506 binding	MF	0.00283
GO:0003755	peptidyl-prolyl cis-trans isomerase activity	MF	0.01541
GO:0016859	cis-trans isomerase activity	MF	0.0184
GO:0006564	L-serine biosynthetic process	BP	0.02339

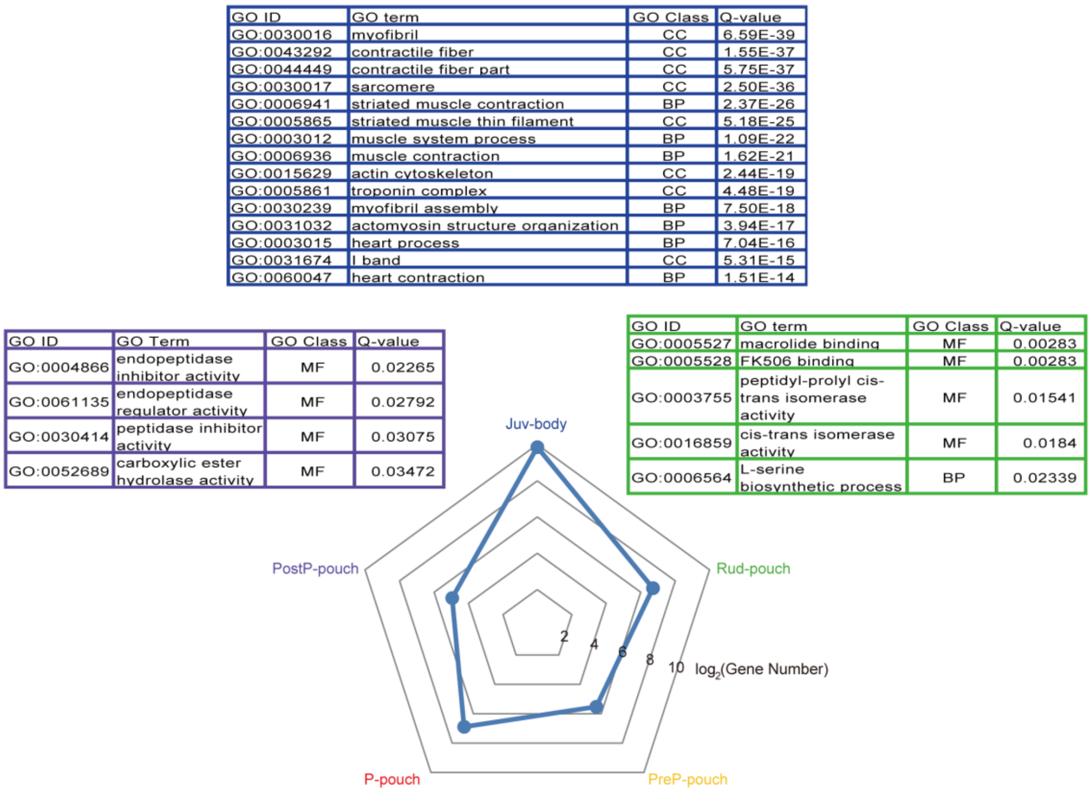

GO ID	GO Term	GO Class	Q-value
GO:0021510	spinal cord development	BP	0.00015
GO:0007420	brain development	BP	0.0002
GO:0001071	nucleic acid binding transcription factor activity	MF	0.00097
GO:0022008	neurogenesis	BP	0.00104
GO:0007417	central nervous system development	BP	0.00113
GO:0030900	forebrain development	BP	0.00117
GO:0048699	generation of neurons	BP	0.00128
GO:0021953	central nervous system neuron differentiation	BP	0.00139
GO:0030182	neuron differentiation	BP	0.00168
GO:0030154	cell differentiation	BP	0.00219
GO:0021515	cell differentiation in spinal cord	BP	0.00246
GO:0021937	cerebellar Purkinje cell-granule cell precursor cell signaling involved in regulation of granule cell precursor cell proliferation	BP	0.00266
GO:0021940	positive regulation of cerebellar granule cell precursor proliferation	BP	0.00266
GO:0007267	cell-cell signaling	BP	0.00355
GO:0003700	sequence-specific DNA binding transcription factor activity	MF	0.0041

GO ID	GO Term	GO Class	Q-value
GO:0005509	calcium ion binding	MF	0.03467

Supplementary Figure 3.1 The tissue-specific expression genes in the brood pouch of five stages. The radar chart shows the specific expression gene number in the brood pouch of five stages. The tables show gene ontology enrichment of genes in the brood pouch of five stages (Q-value < 0.05).

3.3.2 Differential expression of genes during pregnancy stage in seahorse (*Hippocampus erectus*)

The differential expression genes between pregnancy group (brain, gonad and brood pouch of PrP) and non-pregnancy group (brain, gonad and brood pouch of DeP, MaP, and PPrP) were detected using Noiseq[30] with parameters "repl=bio, k = 0.5, norm = n, long = 1000, q = 0.8, pnr = 1, nss = 0, v = 0.02, lc = 1". In total, 47 up-regulated (Supplementary Table 3.5) and 4 down-regulated genes were identified (Supplementary Table 3.6). The up-regulated genes during pregnancy were significantly enriched in the

pathways related to immune, cellular metabolic, and growth (Supplementary Table 3.7 and 3.8). For example, the up-regulated expression of antimicrobial genes (including C-lectin, Interleukin-1 beta, CD40, Annexin A1, Chitinase), may reflect the protection of fertilized eggs or developing embryos by paternal innate immune system. Interestingly, the up-regulated of immune-related genes was also observed in brood pouch of pipefish during pregnancy[31].

The RNA-seq results were validated using quantitative Reverse Transcription PCR (qRT-PCR). In total, the expression profiles of 11 genes were examined at three developmental stages, with five biological replicates of each. qRT-PCR was performed using the Light Cycler®480 Sequence Detection System (Roche, Switzerland) with a 384-well plate. Each reaction involved a 3 min denaturation step at 94℃, then 40 cycles of 94℃ for 15 s, 52℃for 15 s, 72℃ for 30 s, and 85℃ for 1 s to collect fluorescent signals, then a stepwise temperature increase from 52 ℃ to 99 ℃ to establish the melting curve. Standard curves were constructed using a series of 10-fold dilutions of quantified pMD®18-T Simple vector (TaKaRa, China) for each of the target genes. For all target genes, 18S rRNA was used as a reference gene.

All the data were expressed as mean ± standard error of mean (S.E.M) and evaluated by one-way analysis of variance (ANOVA) followed by the Tukey's honestly significant difference test for adjusting P-values from multiple comparisons. Results were considered to be statistically significant for P-values < 0.05. All statistical analyses were carried out using SPSS for Windows, Version 20 (SPSS, Chicago, IL, USA).

Using qRT-PCR, the expression patterns of *Hem*, *Bhs*, *Mylfp*, *Ptr*, *Cc3*, *Cfv II*, *MI*, *HoxA11b*, and *MYL2* were investigated during seahorse pregnancy. During early pregnancy, expression of *MI* in the brood pouch was significantly higher (P < 0.05) than in non-pregnant brood pouch. Additionally, the expression of the expression of *Bhs*, *Cc3*, *Cfv II*, *Hem*, *HoxA11b*, *MI*, *MYL2*, *Mylfp*, and *Ptr* in the brood pouch was significant higher in late pregnancy (P < 0.05), than in the non-pregnant brood pouch (Supplementary Figure 3.2). Furthermore, the expression of *HoxA11b*, *Mylfp* and *Ptr* in the brood pouch was significant higher (P < 0.05) in late pregnancy than in early pregnancy.

Expression of *Ncp* in the brood pouch was during pregnancy than in the non-pregnant group, however the difference was not statistically significant (Supplementary Figure 3.2). However, *Cos* showed a significant reduction in brood pouch expression during late pregnancy group in comparison to the non-pregnant group (Supplementary Figure 3.2).

Supplementary Table 3.5 The up-regulated genes in seahorse (*Hippocampus erectus*) brood pouch during the pregnant stage. These *Hippocampus erectus* transcripts were analyzed with TopHat using *H. comes* gene models as reference. Hence they are identified with *H. comes* gene IDs.

GeneID	log2Ratio(pregnancy/ non_pregnancy)	Qvalue	Function
H.comes.g023240	4.368582	0.838697	AMBP_PLEPL Protein AMBP (Fragment)
H.comes.g003427	3.816643	0.803939	TNNT3_COTJA Troponin T, fast skeletal muscle isoforms
H.comes.g014099	5.907862	0.864027	BHMT1_DANRE Betaine--homocysteine S-methyltransferase 1
H.comes.g021640	7.810274	0.873547	CRGM1_CYPCA Gamma-crystallin M1
H.comes.g021136	4.256193	0.823031	TNNC2_ANGAN Troponin C, skeletal muscle
H.comes.g007877	6.306196	0.844908	REG1A_HUMAN Lithostathine-1-alpha
H.comes.g007819	6.528582	0.883529	FA7_HUMAN Coagulation factor VII
H.comes.g023554	6.115641	0.930673	FA10A_DANRE Fatty acid-binding protein 10-A, liver basic
H.comes.g010585	5.040597	0.808372	ITIH3_MOUSE Inter-alpha-trypsin inhibitor heavy chain H3
H.comes.g022811	7.080438	0.9558	CHIA_BOVIN Acidic mammalian chitinase
H.comes.g022513	5.028519	0.81464	SMU1_MOUSE WD40 repeat-containing protein SMU1
H.comes.g005088	4.920651	0.813709	TNNI2_COTJA Troponin I, fast skeletal muscle
H.comes.g013016	4.896643	0.876137	HEMO_DANRE Hemopexin
H.comes.g023760	5.693401	0.850023	KNG_ANAMI Kininogen (Fragments)
H.comes.g023153	6.383864	0.921515	KCRM_HUMAN Creatine kinase M-type
H.comes.g000631	3.872616	0.816248	TIMP2_CANFA Metalloproteinase inhibitor 2
H.comes.g010468	7.100171	0.840802	MLRV_HUMAN Myosin regulatory light chain 2, ventricular/cardiac muscle isoform
H.comes.g016985	5.182793	0.826913	MLE1_LIZRA Myosin light chain 1, skeletal muscle isoform
H.comes.g002056	4.878129	0.853154	LECT2_HUMAN Leukocyte cell-derived chemotaxin-2
H.comes.g013953	11.85753	0.984533	LCE_ORYLA Low choriolytic enzyme
H.comes.g007746	4.952341	0.850324	MLE3_LIZRA Myosin light chain 3, skeletal muscle isoform
H.comes.g021643	8.415903	0.892204	CRGM3_CYPCA Gamma-crystallin M3
H.comes.g016747	5.180167	0.813487	THRB_PIG Prothrombin
H.comes.g010571	6.851111	0.882732	MYSS_CYPCA Myosin heavy chain, fast skeletal muscle
H.comes.g017603	4.043459	0.843358	A1AT_CYPCA Alpha-1-antitrypsin homolog

H.comes.g005089	4.23044	0.827285	TNNI2_HUMAN Troponin I, fast skeletal muscle
H.comes.g020083	6.97581	0.833973	SAMP_BOVIN Serum amyloid P-component
H.comes.g002569	6.745131	0.903541	DESM_CHICK Desmin
H.comes.g000632	4.355475	0.857355	TIMP2_CANFA Metalloproteinase inhibitor 2
H.comes.g003855	4.655239	0.811468	ACTC_XENTR Actin, alpha cardiac muscle 1
H.comes.g021138	8.295031	0.895578	TNNC2_ANGAN Troponin C, skeletal muscle
H.comes.g002336	6.087464	0.930828	C1QT4_MOUSE Complement C1q tumor necrosis factor-related protein 4
H.comes.g024215	4.187573	0.845676	BHMT1_DANRE Betaine--homocysteine S-methyltransferase 1
H.comes.g010346	5.51736	0.87221	ITIH3_RABIT Inter-alpha-trypsin inhibitor heavy chain H3
H.comes.g013572	4.964468	0.843544	CO3_ONCMY Complement C3 (Fragment)
H.comes.g012628	5.224806	0.854835	MLRS_RABIT Myosin regulatory light chain 2, skeletal muscle isoform type 2
H.comes.g003134	5.191591	0.890033	A1AT_CHLAE Alpha-1-antitrypsin (Fragment)
H.comes.g012260	4.446585	0.843527	APOA1_COTJA Apolipoprotein A-I
H.comes.g008021	4.137217	0.828796	CO2A1_XENTR Collagen alpha-1(II) chain
H.comes.g015637	4.115428	0.822803	FIBB_CHICK Fibrinogen beta chain (Fragment)
H.comes.g012355	5.857476	0.914453	FETUA_MOUSE Alpha-2-HS-glycoprotein
H.comes.g016791	4.573971	0.847785	HEMO_DANRE Hemopexin
H.comes.g003135	5.813314	0.854188	-
H.comes.g010004	5.614165	0.897283	-
H.comes.g020085	7.09891	0.951624	SAMP_PIG Serum amyloid P-component
H.comes.g024552	5.704551	0.903385	ACTS_OREMO Actin, alpha skeletal muscle
NP_001018321.1-D7	6.591019	0.8299	MYSS_CYPCA Myosin heavy chain, fast skeletal muscle

Supplementary Table 3.6 Down-regulated genes in brood pouch during the pregnant stage. These *Hippocampus erectus* transcripts were analyzed with TopHat using *H. comes* gene models as reference. Hence they are identified with *H. comes* gene IDs.

GeneID	log2Ratio(pregnancy/ non_pregnancy)	Qvalue	Function
H.comes.g004099	-5.7008	0.829017	-
H.comes.g011789	-11.1113	0.987819	COX2_GADMO Cytochrome c oxidase subunit 2
H.comes.g016959	-6.92983	0.908501	-
gi_34013700_gb_AAQ56013.1_-D8	-3.98756	0.823406	NCAN_PANTR Neurocan core protein

Supplementary Table 3.7 GO enrichment of genes that were differentially expressed during pregnancy period in seahorse.

GO ID	GO Term	GO	Gene Number	Pvalue
GO:0005865	striated muscle thin filament	CC	5	1.21E-08
GO:0030017	sarcomere	CC	7	4.14E-08
GO:0044449	contractile fiber part	CC	7	8.76E-08
GO:0030016	myofibril	CC	7	1.29E-07
GO:0043292	contractile fiber	CC	7	1.78E-07
GO:0005861	troponin complex	CC	4	7.15E-07
GO:0015629	actin cytoskeleton	CC	7	8.98E-06
GO:0006941	striated muscle contraction	BP	5	1.14E-05
GO:0030239	myofibril assembly	BP	5	9.63E-05
GO:0031032	actomyosin structure organization	BP	5	0.00017
GO:0047150	betaine-homocysteine	MF	2	0.00027
GO:0030049	muscle filament sliding	BP	3	0.00073
GO:0033275	actin-myosin filament sliding	BP	3	0.00073
GO:0008898	homocysteine S-methyltransferase	MF	2	0.00083
GO:0006936	muscle contraction	BP	5	0.00113
GO:0005856	cytoskeleton	CC	9	0.00129
GO:0044430	cytoskeletal part	CC	8	0.00136
GO:0003009	skeletal muscle contraction	BP	3	0.00156
GO:0070252	actin-mediated cell contraction	BP	3	0.00183
GO:0003012	muscle system process	BP	5	0.00234
GO:0006579	amino-acid betaine catabolic process	BP	2	0.00246
GO:0008172	S-methyltransferase activity	MF	2	0.00277
GO:0050881	musculoskeletal movement	BP	3	0.00367
GO:0050879	multicellular organismal movement	BP	3	0.00414
GO:0055002	striated muscle cell development	BP	5	0.00478
GO:0010927	cellular component assembly involved	BP	5	0.0074
GO:0055001	muscle cell development	BP	5	0.00977
GO:0030048	actin filament-based movement	BP	3	0.01122
GO:0030414	peptidase inhibitor activity	MF	3	0.02198
GO:0005576	extracellular region	CC	6	0.02628
GO:0036302	atrioventricular canal development	BP	2	0.02936
GO:0051146	striated muscle cell differentiation	BP	5	0.0316
GO:0060047	heart contraction	BP	3	0.03396
GO:0003015	heart process	BP	3	0.04465
GO:0061134	peptidase regulator activity	MF	3	0.04591

doi:10.1038/nature20595

Supplementary Table 3.8 KEGG pathway enrichment of differentially expressed genes during pregnancy.

Pathway ID	Pathway	Gene Number	Qvalue
ko04610	Complement and coagulation cascades	9	7.95E-09
ko05410	Hypertrophic cardiomyopathy (HCM)	8	1.74E-06
ko04260	Cardiac muscle contraction	7	1.74E-06
ko05414	Dilated cardiomyopathy	8	2.06E-06

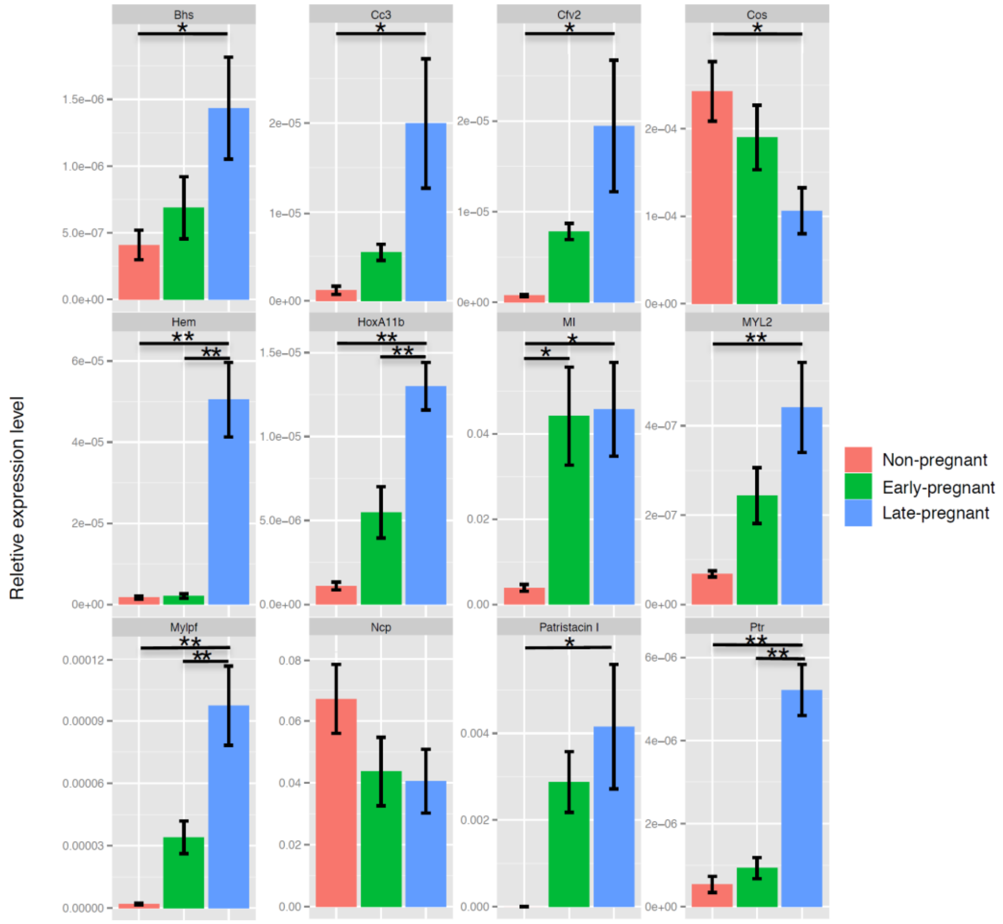

Supplementary Figure 3.2 The differential expression genes in the brood pouch of seahorse during the pregnant stage. Data was presented as mean±S.E.M. (n = 5).

4. Gene family evolution

4.1. Gene family analysis

The gene family analysis was conducted by Treefam using the following steps.

1) Protein sequences of eight ray-fin fishes (zebrafish, *Danio rerio*; stickleback, *Gasterosteus aculeatus*; seahorse, *Hippocampus comes*, Nile tilapia, *Oreochromis niloticus*; medaka, *Oryzias latipes*; fugu, T*akifugu rubripes*, platyfish, *Xiphophorus maculatus* and spotted gar, *Lepisosteus oculatus*) were downloaded from the Ensembl database (Release 75)[32] and the blue-spotted mudskipper *Boleophthalmus pectinirostris* was acquired from the authors[21]. BLASTP[33] was employed to identify potential homologous genes using E-value < 1e-10.
2) The raw Blast results were refined using solar (an in-house software, version 0.9.6) by which the high-scoring segment pairs (HSPs) were conjoined.
3) Similarity between protein sequences were evaluated using bit-score, followed by clustering protein sequences into gene families using hcluster_sg, a hierarchical clustering algorithm in the Treefam pipeline (version 0.50) with the parameters "-w 5 -s 0.33 -m 100000".

The gene family cluster result of ten species is shown in Supplementary Figure 4.1.

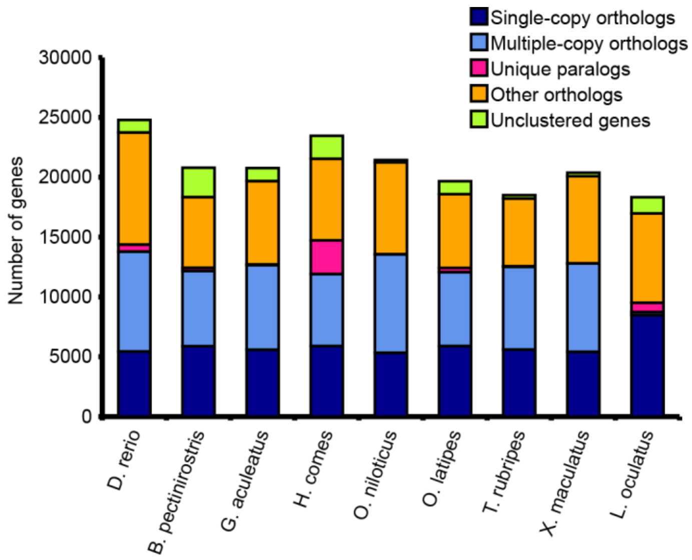

Supplementary Figure 4.1 Gene family evolution in eleven species.
Single-copy orthologs: species contains one copy of orthologous gene.
Multiple-copy orthologs: species contains multiple copies of orthologous gene.
Unique paralogs: lineage specific genes.
Other orthologs: orthologous genes that could only be found in some species but not in all nine species.

4.2 Phylogenetic tree construction and divergence time estimate

We obtained 4122 one-to-one orthologous genes from the gene family analysis using the pipeline described before. The protein sequences of one-to-one orthologous genes were aligned using MUSCLE with the default parameters[34]. We then filtered the saturated sites and poorly aligned regions using trimAl[35] with the parameters “-gt 0.8 –st 0.001 –cons 60”. After trimming the saturated sites and poorly aligned regions in the concatenated alignment, 2,128,000 amino acids were used in the phylogenomics reconstruction. The trimmed protein alignments were used as a guide to align corresponding coding sequences (CDS). The aligned protein and the four-fold degenerated sites in the CDS sequences were each concatenated into a super gene using an in-house Perl script.

The phylogenomics was reconstructed using RAxML version 8.1.19[36] based on the concatenated protein sequences. Specifically, we used PROTGAMMAAUTO parameter to select the optimal amino acid substitution model, specified the spotted gar as the outgroup, and evaluated the robustness of the result using 100 bootstraps. To compare the neutral mutation rate of different species, we also generated a phylogeny based on the four-fold degenerate sites. The phylogenomics topology was used as the input to optimize the branch lengths of alignment of four-fold degenerate sites using the "-f e" parameter in Raxml under the general time reversible (GTR) model, suggested by modelgenerator version 0.85[37]. We calculated the pairwise distances to the outgroup (spotted gar) based on the optimized branch length of neutral tree using the ‘cophenetic.phylo’ module in the R-package‘ape’[38].

4.3 Expansion and contraction of gene families

We used CAFE (version 2.1), a program analyzing gene family expansion and contraction under maximum likelihood framework, in the gene family evolution analysis. The gene family results from Treefam pipeline and the estimated divergence time between species were used as inputs. We used to the parameters "-p 0.01, -r 10000, -s" to search the birth and death parameter (λ) of genes, calculated the probability of each gene family with observed sizes using Monte Carlo10000 random samplings, and reported birth and death parameters in gene families with probability less than 0.01. For the gene family expansion and contraction analysis in seahorse, we first filtered out the gene families without homology in the SWISS-PROT database[23] to reduce the potential false positive expansions or contractions caused by gene prediction. Besides, the families that contain sequences that have multiple functional annotations were also removed. Finally, we found 25 and 54 lineage-specific expansion and contraction of gene families respectively in seahorse (Supplementary Table 4.1 and 4.2)

4.4 Rate of molecular evolution

a) Tajima's relative rate test

The concatenated protein sequences were used in the Tajima's relative rate test (RRT)[39]. RRT is based on three sequences; one outgroup and two ingroups. A significantly higher number of lineage-specific substitutions (based on the Chi-square test) indicate a faster evolution rate of an ingroup in comparison to the other ingroup (Supplementary Table 4.3) and vice versa[39]. In the RRT, we specified spotted gar as the outgroup, and tested the relative evolution rate between seahorse and other fish species.

b) Two cluster analysis

Two cluster analysis, which can be considered as an extension of Tajima's RRT, tests molecular evolution of multiple sequences in a phylogenetic context[40]. A faster or slower rate of evolution of particular taxa was examined using Z-statistics. tpcv module in the LINTRE program[41] was used in the two cluster analysis. Since the direct comparison between seahorse and other species using the complete dataset is not possible, we first converted the concatenated protein alignment to the format that was required by LINTRE and extracted the alignment of the spotted gar, seahorse, and the taxa of interest. In total, seven alignments (Supplementary Table 4.4) and the corresponding tree files were prepared for further analyses.

The branch length of seahorse is longer than that of other fishes in the phylogeny based on the alignment of neutral sites in coding region, which suggests a faster non-synonymous substitution rate in the seahorse lineage in comparison to other fish lineages (Supplementary Fig. 4.3). We used RRT and two cluster analysis to test whether this observation is statistically significant or not. Both methods suggest that seahorse evolves significantly faster than any other species in the analysis (Supplementary Table 4.3 and 4.4).

doi:10.1038/nature20595

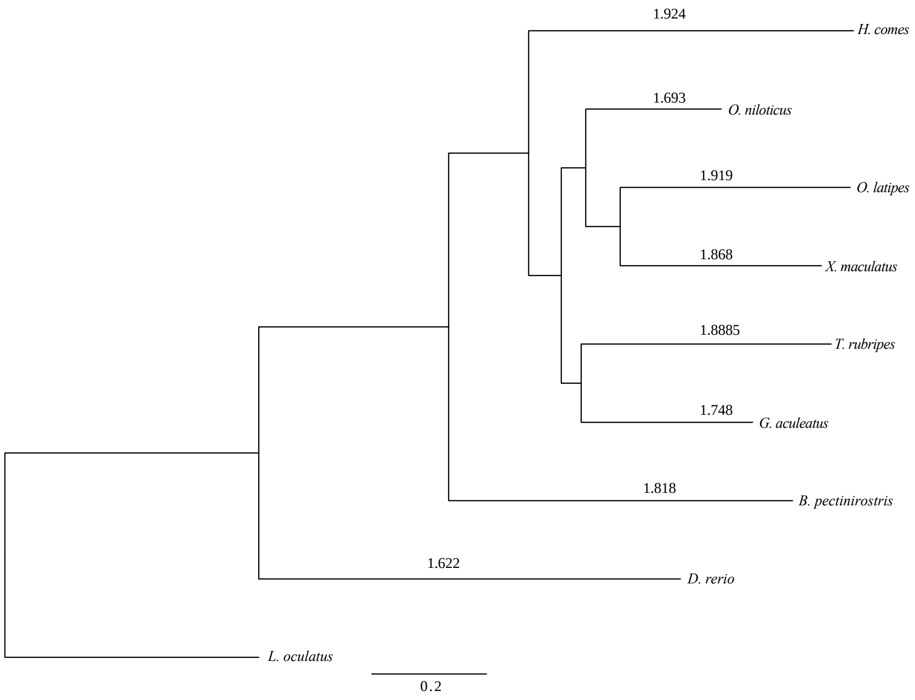

Supplementary Figure 4.3 The phylogeny of seahorse (*H. comes*) and other teleost fishes. The phylogeny was reconstructed using the alignment of four-fold degenerate sites. The value on the branches the branch length of each species, which was calculated using the gar as the outgroup.

Supplementary Table 4.1 Expanded gene families in seahorse.

Fam_id	DANRE	BOLPE	GASAC	HIPCO	ORENI	ORYLA	TAKRU	XIPMA	LEPOC	Gene_Name	Description
1196	7	1	0	4	0	1	0	0	0	Zc3h4	Zinc finger CCCH domain-containing protein 4
1309	0	1	0	3	0	0	1	0	1	chrna3	Neuronal acetylcholine receptor subunit alpha-3
2249	0	29	0	116	2	0	0	5	0	ZNF	Zinc finger protein
2508	0	0	0	6	0	0	4	0	1	Slc6a13	Sodium- and chloride-dependent GABA transporter 2
2619	1	0	8	8	3	0	0	3	1	PGBD4	PiggyBac transposable element-derived protein 4
2696	2	1	0	4	1	0	1	3	1	THAP	THAP domain-containing protein
2771	12	0	0	9	1	8	13	0	2	Vpreb1	Immunoglobulin iota chain
4207	0	1	0	4	0	0	0	0	1	CFDP2	Craniofacial development protein 2
4209	0	0	0	3	0	10	0	0	1	CFDP2	Craniofacial development protein 2
4332	0	0	1	6	4	2	1	0	1	ZBED1/ZBED4	Zinc finger BED domain-containing protein 1/4
4333	0	0	0	7	0	10	0	0	0	ZBED1/ZBED4	Zinc finger BED domain-containing protein 1/4
5698	0	4	0	6	0	0	1	1	0	TCF7	Transcription factor 7
8442	0	1	0	3	0	3	1	0	1	PPP1R3A	Protein phosphatase 1 regulatory subunit
8958	0	0	0	6	0	0	0	0	1	CHIA	Acidic mammalian chitinase
10367	8	0	1	5	1	2	3	2	1	HARBI1	Putative nuclease HARBI1
10691	3	0	0	12	0	0	1	0	0	Tf2-6	Transposon Tf2-6 polyprotein
10859	1	1	3	5	7	2	1	2	0	Natterin-4	Natterin-4
11719	8	1	16	26	37	12	3	1	0	GTF2IRD2	General transcription factor II-I repeat domain-containing protein
11722	0	0	0	5	0	4	0	0	0	SCAND3	SCAN domain-containing protein
12070	1	0	0	4	0	0	0	0	0	ZMYM1	Zinc finger MYM-type protein 1
12763	3	0	3	6	9	1	0	3	0	POGK	Pogo transposable element with KRAB domain

13209	2	0	2	3	0	2	0	0	1	ubq-1	Polyubiquitin-A
15744	0	1	1	4	2	1	0	0	0	Zg16	Zymogen granule membrane protein 16
17361	0	0	0	3	0	0	0	0	0	F5	Coagulation factor V
17579	0	0	0	3	0	1	0	1	0	mt:ATPase6	ATP synthase subunit a

Supplementary Table 4.2 Contracted gene families in seahorse.

Fam_id	DANRE	BOLPE	GASAC	HIPCO	ORENI	ORYLA	TAKRU	XIPMA	LEPOC	Gene_Name	Description
235	6	9	13	4	15	8	10	8	1		Trypsin
237	15	2	7	3	24	19	9	7	6	Prss8	Prostasin
741	19	0	4	1	18	1	5	5	1	CLEC4M	C-type lectin domain family 4 member M
743	9	18	6	0	18	17	0	5	2	MRC	Macrophage mannose receptor
746	76	14	6	1	28	10	0	8	1	MRC	Macrophage mannose receptor
771	11	13	7	2	29	7	4	22	1	C1qtnf	Complement C1q tumor necrosis factor-related protein
960	5	3	1	0	4	2	4	4	3	FFAR3	Free fatty acid receptor 3
1053	50	6	26	2	20	16	7	0	10	Taar	Trace amine-associated receptor
1118	6	2	4	0	4	6	5	2	2	TAAR1	Trace amine-associated receptor 1
1164	19	3	4	0	7	7	9	6	1	OR	Olfactory receptor
1171	2	8	13	1	11	4	4	6	7	OR11A1	Olfactory receptor 11A1
1174	20	1	12	1	6	6	1	4	9	OR1F1	Olfactory receptor 1F1
1186	15	4	2	1	3	8	5	3	3	OR1F12	Olfactory receptor 1F12
1298	6	5	5	0	5	2	1	5	1	HTR3A	5-hydroxytryptamine receptor 3A
1312	7	4	5	1	6	5	2	5	1	Chrnb2	Neuronal acetylcholine receptor subunit beta-2

2116	0	4	3	0	31	0	0	3	17	BTNL2	Butyrophilin-like protein 2
2125	0	22	83	0	36	5	0	1	0		Stonustoxin subunit
2127	287	34	2	1	130	6	7	14	1	NLRC3	Protein NLRC3
2130	22	13	0	0	37	6	12	3	13	NLRC3	Protein NLRC3
2132	6	7	0	0	11	20	22	6	0	RNH1	Ribonuclease inhibitor
2139	34	15	4	0	54	36	4	6	4	Trim16	Tripartite motif-containing protein 16
2145	23	37	53	3	39	7	7	16	6	TRIM	E3 ubiquitin-protein ligase
2152	38	24	28	10	53	12	7	15	6	TRIM	E3 ubiquitin-protein ligase
2270	178	25	29	25	126	36	16	35	1	ZNF	
2617	1	0	0	0	9	3	6	6	8	PGBD3	PiggyBac transposable element-derived protein 3
2759	43	13	18	4	65	29	26	24	10		Ig kappa chain V-I region Walker
2765	1	2	9	0	39	11	32	0	14	Ighv3-6	Ig heavy chain V region 3-6
2772	17	6	6	0	18	16	5	1	62		Ig kappa chain V-*
2773	3	3	20	1	13	11	17	0	9		Ig kappa chain V-IV region B17
2776	3	5	12	1	8	0	4	1	2	V-TCR	Viral T-cell receptor beta chain-like T17T-22
3590	0	8	1	1	7	12	16	4	2	Pim1	Serine/threonine-protein kinase pim-1
4168	3	3	2	0	5	2	3	3	1	Capn1	Calpain-1 catalytic subunit
4563	2	4	5	1	8	4	4	2	1	cldnd	Claudin-like protein ZF-A89
5089	15	7	4	1	5	3	2	5	1	Ugt2a2	UDP-glucuronosyltransferase 2A2
5332	34	0	0	1	5	3	7	7	1	PCDHA8	Protocadherin alpha-8
5356	4	0	1	0	7	3	4	4	2	CDH1	Cadherin-1
5472	15	10	8	0	16	3	9	13	4	IFI44	Interferon-induced protein 44
5903	13	4	0	0	9	5	5	4	1	MFAP4	Microfibril-associated glycoprotein 4
6005	3	5	4	0	3	5	4	4	1	DUSP13	Dual specificity protein phosphatase 13 isoform B
6578	30	4	3	0	28	6	2	6	1	Samd3	Sterile alpha motif domain-containing protein 3

6689	34	10	20	3	11	10	23	4	1	zgc:112234	Histone H2B 1/2
6690	30	17	23	8	10	9	22	3	1		Histone H2A
6853	21	4	10	3	28	7	4	16	2	GIMAP	GTPase IMAP family member
6857	10	8	1	0	11	3	1	2	2	GIMAP	GTPase IMAP family member
6858	6	7	9	0	6	3	0	5	1	GIMAP	GTPase IMAP family member
7097	12	1	6	1	11	6	5	7	3	Cyp2d1	Cytochrome P450 2D1
8283	3	3	8	0	3	2	3	3	1	Psmb7	Proteasome subunit beta type-7
8284	7	7	23	3	6	6	8	7	1	PSMB	Proteasome subunit beta type
10329	7	4	0	0	7	0	6	0	1	IRGC	Interferon-inducible GTPase 5
10526	36	2	22	5	13	17	11	1	8		Gamma-crystallin M3
10795	8	2	6	1	5	4	1	5	2	hbb2	Hemoglobin subunit beta-2
10892	14	10	3	1	4	7	2	12	5	Art1	GPI-linked NAD(P)(+)--arginine ADP-ribosyltransferase 1
11005	16	16	21	4	0	10	14	4	2		Histone H3
13592	6	1	7	1	7	6	1	4	1	IFIT1	Interferon-induced protein with tetratricopeptide repeats 1

Supplementary Table 4.3. Relative rate test of seahorse and other teleost fishes. The chi-square test was based on 1 degree of freedom.

Outgroup	Ingroup 1	Ingroup2	Identical	Ingroup 1 specific	Ingroup2 species	Chi-score	P-value	Faster
gar	seahorse	stickleback	1331337	113468	88885	2986.48347	<0.00001	seahorse
gar	seahorse	tilapia	1350761	118489	69461	12789.27791	<0.00001	seahorse
gar	seahorse	zebrafish	1291940	178187	128282	8126.46312	<0.00001	seahorse
gar	seahorse	platyfish	1328126	117363	92096	3047.95349	<0.00001	seahorse
gar	seahorse	medaka	1313994	116132	106228	441.12797	<0.00001	seahorse
gar	seahorse	mudskipper	1309593	116182	110629	135.95376	<0.00001	seahorse
gar	seahorse	fugu	1319540	113803	100682	802.66984	<0.00001	seahorse

Supplementary Table 4.4. Two cluster analysis of seahorse and other teleost fishes. The branch length was calculated using gar as the outgroup. Z-statistic was used to test whether the distances between ingroups (bA, bB) to outgroup is significantly different to 0 or not. Delta is the absolute difference between bA and bB. Z-statistics (Z) is delta/standard error (s.e.). CP (confident probability) is equal to 1- P-value.

Outgroup	Ingroup A	Ingroup B	bA	bB	delta	s.e.	Z	CP	Faster
gar	seahorse	stickleback	0.1216	0.0973	0.0242	0.0004	54.5639	99.96%	seahorse
gar	seahorse	tilapia	0.1246	0.0771	0.0475	0.0004	112.3544	99.96%	seahorse
gar	seahorse	zebrafish	0.1952	0.1469	0.0483	0.0005	89.7733	99.96%	seahorse
gar	seahorse	platyfish	0.1273	0.1024	0.0249	0.0005	55.1208	99.96%	seahorse
gar	seahorse	medaka	0.1281	0.1182	0.0099	0.0005	20.9982	99.96%	seahorse
gar	seahorse	mudskipper	0.1297	0.1241	0.0056	0.0005	11.6591	99.96%	seahorse
gar	seahorse	fugu	0.1258	0.1127	0.0130	0.0005	28.3195	99.96%	seahorse

5. Detection of positively selected genes

Using the methods described in Supplementary Method 4.1, we first did gene family analysis of ten teleost genomes (zebrafish, *D. rerio;* cod, *G. morhua;* stickleback, *G. aculeatus;* seahorse, *H. comes;* Nile tilapia, *O. niloticus;* medaka, *O. latipes;* fugu, *T. rubripes;* Tetraodon, *T. nigroviridis;* Platyfish, *X. maculatus;* and coelacanth, *L. chalumnae*). In total, 3,721 one-to-one orthologous genes were sused in the positive selection analysis. To increase the accuracy of alignment, we first aligned the protein sequences using MUSCLE[34] with default parameters. Then, the protein alignment was employed as a guild for aligning CDS. Positive selection analysis was conducted using the refined branch-site model[42] which is implemented in the codeml program of PAML package[43] (version 4). We compared ModelA1, in which sites may evolve neutrally and under purifying selection with ModelA that allows sites to be also under positive selection. P-values were computed using the X2 statistic adjusted by the false discovery rate (FDR) method to allow for multiple testing.

Using the pipeline that was described before, a total of 572 genes were under positive selection in seahorse (FDR adjusted P-value< 0.05) (Supplementary Table 5.1).

Supplementary Table 5.1 GO enrichment test of positively selected genes in seahorse

GO_ID	GO_Term	GO_Class	X1	X2	P-value
GO:0090304	nucleic acid metabolic process	BP	77	1385	0.00013
GO:0044260	cellular macromolecule metabolic process	BP	148	3385	0.00031
GO:0016070	RNA metabolic process	BP	55	956	0.00477
GO:1901360	organic cyclic compound metabolic process	BP	104	2251	0.00498
GO:0006725	cellular aromatic compound metabolic process	BP	99	2122	0.00622
GO:0046483	heterocycle metabolic process	BP	98	2105	0.00781
GO:0044428	nuclear part	CC	89	1944	0.00803
GO:0006139	nucleobase-containing compound metabolic process	BP	93	1982	0.01086
GO:0031981	nuclear lumen	CC	79	1687	0.01181
GO:0034641	cellular nitrogen compound metabolic process	BP	102	2243	0.01327
GO:0043170	macromolecule metabolic process	BP	152	3741	0.02085
GO:0005730	nucleolus	CC	45	822	0.02196
GO:0070013	intracellular organelle lumen	CC	86	1926	0.02817
GO:0009059	macromolecule biosynthetic process	BP	58	1098	0.0336
GO:0000930	gamma-tubulin complex	CC	4	10	0.03403
GO:0044237	cellular metabolic process	BP	190	4966	0.03455
GO:0043233	organelle lumen	CC	86	1938	0.03504
GO:0031974	membrane-enclosed lumen	CC	87	1973	0.03955
GO:0071704	organic substance metabolic process	BP	196	5170	0.04019
GO:0034645	cellular macromolecule biosynthetic process	BP	56	1056	0.04259

BP: Biological process. CC: Cellular Component.

X1: number of genes under positive selection within the GO term, X2: total number of genes within the GO term.

Supplementary Table 5.2 KEGG enrichment test for positively selected genes in seahorse.

Pathway ID	Pathway	Gene Number	Qvalue
ko03008	Ribosome biogenesis in eukaryotes	12	0.1059851
ko00740	Riboflavin metabolism	3	0.6429771
ko00450	Selenocompound metabolism	3	0.6429771
ko00970	Aminoacyl-tRNA biosynthesis	6	0.6429771
ko03420	Nucleotide excision repair	6	0.6429771
ko03030	DNA replication	4	0.9981941
ko00511	Other glycan degradation	3	0.9981941
ko03440	Homologous recombination	4	0.9981941
ko03430	Mismatch repair	3	0.9981941
ko05131	Shigellosis	10	0.999996
ko03013	RNA transport	23	0.999996
ko03015	mRNA surveillance pathway	16	0.999996
ko00600	Sphingolipid metabolism	5	0.999996
ko00260	Glycine, serine and threonine metabolism	4	0.999996
ko00380	Tryptophan metabolism	4	0.999996
ko03460	Fanconi anemia pathway	5	0.999996
ko04512	ECM-receptor interaction	14	0.999996
ko00860	Porphyrin and chlorophyll metabolism	3	0.999996
ko00650	Butanoate metabolism	3	0.999996
ko05132	Salmonella infection	14	0.999996
ko00040	Pentose and glucuronate interconversions	4	0.999996
ko00561	Glycerolipid metabolism	5	0.999996
ko00670	One carbon pool by folate	2	0.999996
ko04064	NF-kappa B signaling pathway	9	0.999996
ko04810	Regulation of actin cytoskeleton	27	0.999996
ko00750	Vitamin B6 metabolism	1	0.999996
ko04146	Peroxisome	6	0.999996
ko04622	RIG-I-like receptor signaling pathway	4	0.999996
ko04975	Fat digestion and absorption	4	0.999996
ko05220	Chronic myeloid leukemia	7	0.999996
ko00072	Synthesis and degradation of ketone bodies	1	0.999996
ko05100	Bacterial invasion of epithelial cells	9	0.999996

ko00340	Histidine metabolism	2	0.999996
ko04142	Lysosome	8	0.999996
ko03040	Spliceosome	13	0.999996
ko00603	Glycosphingolipid biosynthesis - globo series	1	0.999996
ko00460	Cyanoamino acid metabolism	1	0.999996
ko05110	Vibrio cholerae infection	16	0.999996
ko05146	Amoebiasis	23	0.999996
ko05130	Pathogenic Escherichia coli infection	10	0.999996
ko04350	TGF-beta signaling pathway	7	0.999996
ko00310	Lysine degradation	6	0.999996
ko03018	RNA degradation	5	0.999996
ko00280	Valine, leucine and isoleucine degradation	3	0.999996
ko00565	Ether lipid metabolism	3	0.999996
ko04620	Toll-like receptor signaling pathway	5	0.999996
ko05169	Epstein-Barr virus infection	16	0.999996
ko04610	Complement and coagulation cascades	6	0.999996
ko03022	Basal transcription factors	3	0.999996
ko04120	Ubiquitin mediated proteolysis	9	0.999996
ko05134	Legionellosis	6	0.999996
ko04510	Focal adhesion	22	0.999996
ko04270	Vascular smooth muscle contraction	16	0.999996
ko05219	Bladder cancer	3	0.999996
ko04141	Protein processing in endoplasmic reticulum	11	0.999996
ko05416	Viral myocarditis	10	0.999996
ko04110	Cell cycle	8	0.999996
ko04722	Neurotrophin signaling pathway	9	0.999996
ko04666	Fc gamma R-mediated phagocytosis	10	0.999996
ko00520	Amino sugar and nucleotide sugar metabolism	4	0.999996
ko03410	Base excision repair	2	0.999996
ko04210	Apoptosis	5	0.999996
ko00900	Terpenoid backbone biosynthesis	1	0.999996
ko00360	Phenylalanine metabolism	1	0.999996
ko05144	Malaria	3	0.999996
ko00010	Glycolysis / Gluconeogenesis	3	0.999996
ko04115	p53 signaling pathway	5	0.999996
ko03060	Protein export	1	0.999996
ko04623	Cytosolic DNA-sensing pathway	2	0.999996

ko00620	Pyruvate metabolism	2	0.999996
ko00071	Fatty acid metabolism	2	0.999996
ko04974	Protein digestion and absorption	8	0.999996
ko00330	Arginine and proline metabolism	3	0.999996
ko02010	ABC transporters	4	0.999996
ko05168	Herpes simplex infection	9	0.999996
ko05410	Hypertrophic cardiomyopathy (HCM)	11	0.999996
ko00510	N-Glycan biosynthesis	2	0.999996
ko00100	Steroid biosynthesis	1	0.999996
ko04140	Regulation of autophagy	1	0.999996
ko05414	Dilated cardiomyopathy	11	0.999996
ko04621	NOD-like receptor signaling pathway	3	0.999996
ko00512	Mucin type O-Glycan biosynthesis	1	0.999996
ko04260	Cardiac muscle contraction	8	0.999996
ko04013	MAPK signaling pathway - fly	1	0.999996
ko00630	Glyoxylate and dicarboxylate metabolism	1	0.999996
ko04710	Circadian rhythm - mammal	1	0.999996
ko05162	Measles	7	0.999996
ko04340	Hedgehog signaling pathway	3	0.999996
ko04145	Phagosome	9	0.999996
ko00052	Galactose metabolism	2	0.999996
ko04971	Gastric acid secretion	6	0.999996
ko04612	Antigen processing and presentation	4	0.999996
ko00410	beta-Alanine metabolism	1	0.999996
ko04966	Collecting duct acid secretion	1	0.999996
ko00982	Drug metabolism - cytochrome P450	1	0.999996
ko04360	Axon guidance	11	0.999996
ko00250	Alanine, aspartate and glutamate metabolism	1	0.999996
ko05145	Toxoplasmosis	7	0.999996
ko00030	Pentose phosphate pathway	1	0.999996
ko00500	Starch and sucrose metabolism	3	0.999996
ko04964	Proximal tubule bicarbonate reclamation	1	0.999996
ko05166	HTLV-I infection	13	0.999996
ko05223	Non-small cell lung cancer	3	0.999996
ko05212	Pancreatic cancer	3	0.999996
ko00640	Propanoate metabolism	1	0.999996
ko00053	Ascorbate and aldarate metabolism	1	0.999996

ko05217	Basal cell carcinoma	4	0.999996
ko04630	Jak-STAT signaling pathway	4	0.999996
ko00980	Metabolism of xenobiotics by cytochrome P450	1	0.999996
ko05160	Hepatitis C	5	0.999996
ko05142	Chagas disease (American trypanosomiasis)	5	0.999996
ko04010	MAPK signaling pathway	15	0.999996
ko00270	Cysteine and methionine metabolism	1	0.999996
ko04950	Maturity onset diabetes of the young	1	0.999996
ko04144	Endocytosis	13	0.999996
ko05222	Small cell lung cancer	4	0.999996
ko04721	Synaptic vesicle cycle	3	0.999996
ko04062	Chemokine signaling pathway	9	0.999996
ko04664	Fc epsilon RI signaling pathway	3	0.999996
ko05202	Transcriptional misregulation in cancer	9	0.999996
ko00760	Nicotinate and nicotinamide metabolism	1	0.999996
ko04380	Osteoclast differentiation	5	0.999996
ko00480	Glutathione metabolism	1	0.999996
ko04920	Adipocytokine signaling pathway	3	0.999996
ko04530	Tight junction	13	0.999996
ko04662	B cell receptor signaling pathway	4	0.999996
ko03320	PPAR signaling pathway	3	0.999996
ko00562	Inositol phosphate metabolism	3	0.999996
ko05140	Leishmaniasis	2	0.999996
ko05152	Tuberculosis	8	0.999996
ko05120	Epithelial cell signaling in Helicobacter pylori infection	2	0.999996
ko04330	Notch signaling pathway	2	0.999996
ko04520	Adherens junction	6	0.999996
ko00830	Retinol metabolism	1	0.999996
ko05221	Acute myeloid leukemia	2	0.999996
ko00240	Pyrimidine metabolism	5	0.999996
ko05340	Primary immunodeficiency	1	0.999996
ko00051	Fructose and mannose metabolism	1	0.999996
ko04640	Hematopoietic cell lineage	2	0.999996
ko05213	Endometrial cancer	2	0.999996
ko04961	Endocrine and other factor-regulated calcium reabsorption	2	0.999996
ko05200	Pathways in cancer	18	0.999996

ko04320	Dorso-ventral axis formation	1	0.999996
ko05210	Colorectal cancer	2	0.999996
ko05412	Arrhythmogenic right ventricular cardiomyopathy (ARVC)	3	0.999996
ko05164	Influenza A	7	0.999996
ko04514	Cell adhesion molecules (CAMs)	5	0.999996
ko00564	Glycerophospholipid metabolism	2	0.999996
ko04012	ErbB signaling pathway	3	0.999996
ko05014	Amyotrophic lateral sclerosis (ALS)	3	0.999996
ko05020	Prion diseases	3	0.999996
ko00190	Oxidative phosphorylation	2	0.999996
ko05211	Renal cell carcinoma	2	0.999996
ko04670	Leukocyte transendothelial migration	6	0.999996
ko05215	Prostate cancer	3	0.999996
ko04962	Vasopressin-regulated water reabsorption	2	0.999996
ko04978	Mineral absorption	1	0.999996
ko04960	Aldosterone-regulated sodium reabsorption	1	0.999996
ko05323	Rheumatoid arthritis	1	0.999996
ko05214	Glioma	2	0.999996
ko04976	Bile secretion	3	0.999996
ko04660	T cell receptor signaling pathway	3	0.999996
ko00230	Purine metabolism	7	0.999996
ko05218	Melanoma	1	0.999996
ko04912	GnRH signaling pathway	3	0.999996
ko04070	Phosphatidylinositol signaling system	3	0.999996
ko04914	Progesterone-mediated oocyte maturation	2	0.999996
ko04310	Wnt signaling pathway	6	0.999996
ko04910	Insulin signaling pathway	5	0.999996
ko01100	Metabolic pathways	44	0.999996
ko04150	mTOR signaling pathway	1	0.999996
ko05133	Pertussis	1	0.999996
ko05010	Alzheimer's disease	4	0.999996
ko04540	Gap junction	2	0.999996
ko04720	Long-term potentiation	2	0.999996
ko04972	Pancreatic secretion	2	0.999996
ko04724	Glutamatergic synapse	3	0.999996
ko05012	Parkinson's disease	1	0.999996
ko04060	Cytokine-cytokine receptor	2	0.999996

	interaction		
ko03020	RNA polymerase	1	0.999996
ko04370	VEGF signaling pathway	1	0.999996
ko05034	Alcoholism	2	0.999996
ko04650	Natural killer cell mediated cytotoxicity	1	0.999996
ko04114	Oocyte meiosis	1	0.999996
ko04916	Melanogenesis	2	0.999996
ko04020	Calcium signaling pathway	4	0.999996
ko05016	Huntington's disease	4	0.999996
ko04726	Serotonergic synapse	1	0.999996
ko04970	Salivary secretion	2	0.999996
ko04080	Neuroactive ligand-receptor interaction	2	0.999996

6. Loss of conserved noncoding elements (CNEs)

Methods

Genome-wide conserved noncoding element (CNE) prediction

Whole-genome alignments

Using zebrafish as the reference genome, whole-genome alignments of six teleost fishes were generated. The soft-masked genome sequence for zebrafish (Zv9, Apr-2010) was downloaded from Ensembl release-75 FTP site. The following soft-masked genome sequences were downloaded from the UCSC Genome Browser: stickleback (gasAcu1, Feb-2006), fugu (fr3, Oct-2011), medaka (oryLat2, Oct-2005), Nile tilapia (oreNil2, Feb-2012). The *H. comes* genome sequence (hipCom0) was repeat-masked using WindowMasker (from NCBI BLAST+ package v2.2.28) with additional parameter "-dust true". About 32% (158.1 / 501.6 Mb) of the *H. comes* genome was masked using this method.

Only chromosome sequences of zebrafish were aligned while unplaced scaffolds were excluded. The reference (zebrafish) genome was split into 21 Mb sequences with 10-kb overlap, while the percomorph fish genomes (seahorse, stickleback, fugu, medaka and Nile tilapia) were split into 10 Mb sequences with no overlap. Pairwise alignments were carried out using Lastz v1.03.54[44] with the following parameters: --strand=both --seed=12of19 --notransition --chain --gapped --gap=400,30 --hspthresh=3000 --gappedthresh=3000 --inner=2000 --masking=50 --ydrop=9400 --scores=HoxD55.q --format=axt. Coordinates of split sequences were restored to genome coordinates using an in-house Perl script. The alignments were reduced to single-coverage with respect to the reference genome using UCSC Genome Browser tools 'axtChain' and 'chainNet'. Multiple alignments were generated using Multiz.v11.2/roast.v3 [45] with the tree topology "(Zv9 (hipCom0 ((fr3 gasAcu1) (oryLat2 oreNil2))))" [46,47].

Identification of conserved elements

Four-fold degenerate (4D) sites of zebrafish genes (Ensembl release-75) were extracted from the multiple alignments. These 4D sites were used to build a neutral model using PhyloFit in the rphast v1.5 package[48] (general reversible "REV" substitution model). PhastCons was then run in rho-estimation mode on each of the zebrafish chromosomal alignments to obtain a conserved model for each chromosome. These conserved models were averaged into one model using PhyloBoot. Subsequently, conserved elements were predicted in the multiple alignments using PhastCons with the following inputs and parameters: the neutral and conserved models, target coverage of input alignments = 0.3 and average length of conserved sequence = 45 bp. To assess the sensitivity of this approach in identifying functional elements, the PhastCons elements were compared against zebrafish protein-coding genes. 80% of protein-coding exons (197,508/245,556 exons) were overlapped by a conserved element (minimum coverage 10%), indicating

that the identification method was fairly sensitive.

Classification of conserved elements

The conserved elements were classified as follows. Firstly, elements that were shorter than 30 bp were excluded. Secondly, the conserved elements were segregated into repetitive sequences (≥30% of bases were repeat-masked), exonic (overlapping known protein-coding genes or noncoding RNA in Ensembl-75 zebrafish gene-build), and non-exonic. Thirdly, the non-exonic elements were filtered against zebrafish mRNAs (~33,000), spliced ESTs (1.3 million) downloaded from the UCSC Genome Browser for 'danRer7' assembly, and vertebrate proteins obtained from Uniprot (~238,000 proteins in 'complete' and 'reference' vertebrate proteomes; BLASTX at 1e-5). A total of 638,280 conserved elements (≥ 30 bp) that span 9.0% (122.5/1,357.1 Mb) of the zebrafish genome were identified. The conserved elements that were neither exonic based on known genes, nor potentially protein-coding, nor repetitive sequence were classified as conserved noncoding elements (CNEs). Finally, each of the zebrafish CNEs was assigned to the protein-coding gene whose transcription start site was nearest to the CNE within 1 Mb in the genome (Ensembl release-75 zebrafish genes).

Conservation or loss of CNEs in teleost fish genomes

A CNE was considered present in a percomorph genome if it showed a coverage of at least 30% with a zebrafish CNE in Multiz alignment. To identify CNEs that could have been missed in the Multiz alignments due to rearrangements in the genomes, or due to partitioning of the CNEs among teleost fish duplicate genes, we searched the zebrafish CNEs against the genome of the percomorph using BLASTN (E<1e-10; ≥80% identity; ≥30% coverage). Those CNEs that had no significant match in a percomorph genome were considered as missing in that genome. In order to account for CNEs that might have been missed due to sequencing gaps, we identified gap-free syntenic intervals in zebrafish and the percomorph genomes, and generated a set of CNEs that were missing from these intervals. These CNEs represent a high-confident set of CNEs missing in the percomorph fishes and thus were used for further analysis.

Functional enrichment of genes associated with CNEs

Functional enrichment of genes associated with CNEs was carried out using the GREAT software[49] with each CNE assigned to the genes with the nearest transcription start site and within 1 Mb in the zebrafish genome, and significantly enriched functional categories identified based on a hypergeometric test of genomic regions (FDR q-value < 0.05). We identified the statistically significant GO biological process terms, molecular function terms and zebrafish phenotype descriptions of the genes that are associated with CNEs.

Identification CNEs in Hox clusters

Repeat-masked Hox cluster sequences of zebrafish, fugu, stickleback and medaka were extracted from UCSC (http://genome-euro.ucsc.edu/). Repeat sequences in the seahorse

Hox clusters were masked using the CENSOR repeat-masking web-server (http://www.girinst.org/censor/index.php). Orthologous, repeat-masked Hox cluster sequences (HoxAa, HoxAb, HoxBa, HoxBb, HoxCa, HoxDa and HoxDb) from the five teleosts were aligned using the global alignment program MLAGAN[50] with the zebrafish sequence as the base. CNEs were predicted using VISTA[51] at a cut-off of ≥70% identity over >100 bp windows. For counting CNEs that are uniquely lost in a fish, we considered only non-gap regions of the Hox cluster sequences.

Transgenic assay of CNEs

Seven representative zebrafish CNEs that are lost in seahorse (the largest among the lost CNEs) were assayed for enhancer activity in transgenic zebrafish using GFP as the reporter gene. For every CNE that we selected for functional assay, we identified the gene with the nearest transcription start site as the associated gene. To confirm the absence of these CNEs in seahorse, we realigned the loci of the associated genes in zebrafish, seahorse, stickleback, fugu, medaka and Nile tilapia, including the immediate flanking genes. The realignment was carried out using the global alignment program MLAGAN[50] and the alignment was visualized using VISTA[51] with sequence conservation criteria of 70% identity over 100 bp.

The CNEs were amplified by PCR using zebrafish genomic DNA as template. The products were cloned into a miniTol2 transposon donor plasmid linked to the mouse *cFos* (McFos) basal promoter and the coding sequence of GFP. Transposase mRNA was generated by transcribing cDNA *in vitro* using the mMESSAGE mMACHINE T7 kit (Ambion; Life Technologies, United States). The CNE-containing McFos-miniTol2 construct and transposase mRNA were co-injected into the yolk of zebrafish embryos at the one to two-cell stage. Each CNE construct was injected into 250-350 embryos and the injections were repeated on two days. The embryos were reared at 28°C, and GFP was observed at 24, 48 and 72 hours post fertilization (hpf). The survival rate of the embryos post-injection was 70-80%. Consistent GFP expression in at least 20% of F0 embryos was considered as specific expression driven by a CNE. Such embryos were reared to maturity and mated with wild type zebrafish to produce F1 lines. The expression of GFP in F1 embryos was observed under a compound microscope fitted for epifluorescence (Axio imager M2; Carl Zeiss, Germany) and photographed using an attached digital microscope camera (Axiocam; Carl Zeiss, Germany). Pigmentation was inhibited by maintaining zebrafish embryos in 0.003% N-phenylthiourea (Sigma-Aldrich, Sweden) from 8 hpf onwards. Consistent GFP expression observed in at least three lines of F1 fishes was considered as the specific expression driven by a CNE.

Results

Identification of genome-wide CNEs

CNEs were predicted in the genomes of seahorse and five representative teleost fishes (zebrafish, stickleback, fugu, medaka and Nile tilapia). Zebrafish represents the

Ostariophysi clade whereas the rest are members of the Percomorpha clade. Ostariophysi and Percomorpha constitute the two most distantly related clades of the clupeocephalan lineage[46] which includes ~96% of extant teleosts [52]. The clupeocephalans shared a common ancestor approximately 275 million years ago [47]. For CNE prediction, whole genome sequences were aligned using Multiz with zebrafish as the reference genome (see Methods). A non-conserved model was estimated from the four-fold degenerate sites of the multi-genome alignment and was used to predict genomic regions that were under higher selective constraint than neutrally evolving DNA. After filtering protein-coding and RNA gene sequences (see Methods), a total of 239,976 conserved noncoding elements (CNEs) were identified. These CNEs are noncoding regions that are under evolutionary constraint in zebrafish and at least one of the five percomorph fishes over the last 275 million years of evolution. They total 40.3 Mb, have an average size of 168 bp and span 3.0% of the zebrafish genome.

We determined the coverage of the CNEs in each of the percomorph genomes. The species in descending order of number of CNEs are: Nile tilapia (135,573 CNEs), medaka (103,198 CNEs), stickleback (96,647 CNEs), seahorse (80,857 CNEs) and fugu (69,623 CNEs) (Supplementary Table 6.2). Among these CNEs, a set of 17,744 CNEs (average 197 bp) are present in all the six teleost fishes, which we term as "pan-teleost" CNEs (Supplementary Table 6.2). The pan-teleost CNEs likely play a fundamental role in the development, morphology and physiology of the teleost fishes. To determine the functions of genes associated with these pan-teleost CNEs, we first assigned each CNE to the gene whose transcription start site was nearest to the CNE in the zebrafish genome. A total of 4,059 genes were found to be associated with these CNEs. Next we carried out functional enrichment analysis of the 17,744 pan-teleost CNEs (average 197 bp) against all CNEs as the background dataset using the GREAT software[49]. Pan-teleost CNEs were found to be preferentially associated with genes that are involved in the development of the midbrain-hindbrain boundary, cerebellum, eye and rostrocaudal patterning of the neutral tube (Supplementary Table 6.5), genes that encode steroid hormone receptors, O-methyltransferases, or protein transporters (Supplementary Table 6).

Genome-wide CNE loss

To determine the extent of CNEs lost in seahorse, we searched for CNEs that are uniquely lost in each of the percomorph fishes. We accounted for CNEs that may not have been detected in the Multiz alignments due to partitioning of duplicated genomic regions (see Methods) and restricted our analyses to a high-confidence set of CNEs situated in gap-free syntenic intervals (Supplementary Table 6.3). Interestingly, seahorse was found to have lost the highest number of CNEs that are conserved in the other percomorphs. In total, 1,612 CNEs were lost in seahorse which is substantially higher than that lost in fugu (1,050 CNEs), stickleback (843 CNEs), medaka (335 CNEs) and Nile tilapia (281 CNEs) (Supplementary Table 6.4).
To determine the type of genes associated with CNEs specifically lost in seahorse, we

first identified the homologous zebrafish CNEs and then assigned these CNEs to their nearest gene in the zebrafish genome. The zebrafish CNEs whose homologs are lost in seahorse are associated with a total of 728 genes. To identify the functional categories of these genes and the pathways they are involved in, we carried out functional enrichment analysis against genes associated with all the CNEs in the zebrafish genome. The genes associated with the CNEs lost in seahorse are involved in the regulation of transcription, positive regulation of fibroblast growth factor receptor signaling pathway and embryonic pectoral fin morphogenesis (Supplementary Table 6.8). They also possess DNA-binding transcription factor activity, steroid hormone receptor activity and O-acetyltransferase activity (Supplementary Table 6.9).

The top 20 genes with the highest number and proportion of CNEs lost uniquely in seahorse genome are listed in Supplementary Table 6.10 and Supplementary Table 6.11, respectively. All these genes are conserved in seahorse despite the loss of a large number of CNEs associated with them. They include *Sall1a* (Sal-like protein 1a), *Shox* (short stature homeobox), and *Irx5a* (Iroquois homeobox protein 5a) genes. *Sall1* together with other members of the family of *spalt* genes, are required for the normal development of limbs, nervous system, kidney and heart in vertebrates [53]. *Sall1* is regulated by FGF and WNT signaling in developing limb buds of chicken [54]. In zebrafish, *Sall1* is regulated by *tbx5* in pectoral fin development and is required for regulation of FGF signaling along with *sall4*[55]. *Shox* encodes a transcription factor that is involved in skeletal system development. Mutations in the human *SHOX* gene result in Leri-Weill dyschondrosteosis (LWD), a dominantly inherited skeletal dysplasia that is characterized by moderate short stature caused mainly by short mesomelic limb segments[56]. A ~200-kb human genomic interval was found to be deleted in individuals who had two intact copies of *SHOX* and yet displayed LWD-type malformations associated with *SHOX* haploinsufficiency[57]. The seahorse genomic region orthologous to this interval has lost several teleost CNEs. In developing zebrafish embryos, *shox* is expressed in the blood, putative heart, hatching gland, brain pharyngeal arch, olfactory epithelium, and fin bud apical ectodermal ridge[58]. *Irx5* is part of the Iroquois family of genes and is required for the formation of the proximal and anterior limb skeleton[59], differentiation of retinal bipolar interneurons[60] and establishing the cardiac ventricular repolarization gradient in mouse[61]. In human, mutations in the IRX5 homeodomain result in a recessive congenital disorder that affects development of the face, brain, blood, heart, bone and gonads[62]. It is possible that loss of CNEs associated with these genes in seahorse could have led to phenotypic changes.

CNEs lost from Hox clusters

We predicted CNEs in the Hox clusters of seahorse and other representative teleost fishes using the global alignment program MLAGAN. Orthologous Hox clusters were aligned using MLAGAN with zebrafish as the reference sequence and CNEs were predicted using VISTA. VISTA plots of two representative Hox clusters, HoxCa and HoxDa, are shown in Supplementary Figure 2 and Supplementary Figure 3, respectively.

Interestingly, seahorse shares the least number of CNEs with zebrafish (186 CNEs) when compared to fugu, stickleback or medaka (214 to 235 CNEs) (Supplementary Table 6.7). We next searched for CNEs that were uniquely lost in seahorse, fugu, stickleback and medaka. Indeed, seahorse was found to have lost the highest number of CNEs (23 CNEs) compared to the other three teleosts (fugu: 4 CNEs, stickleback: 2 CNEs and medaka: 9 CNEs).

Functional assay of CNEs

Details of the seven zebrafish CNEs selected for functional assay in transgenic zebrafish and their expression patterns are presented in Supplementary Table 6.12.

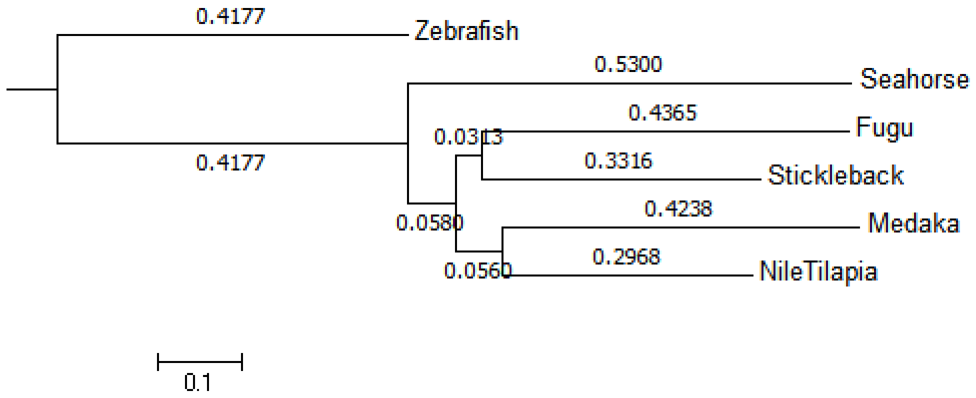

Supplementary Figure 6.1 Neutral tree for teleost fish genomes used in whole-genome alignments.

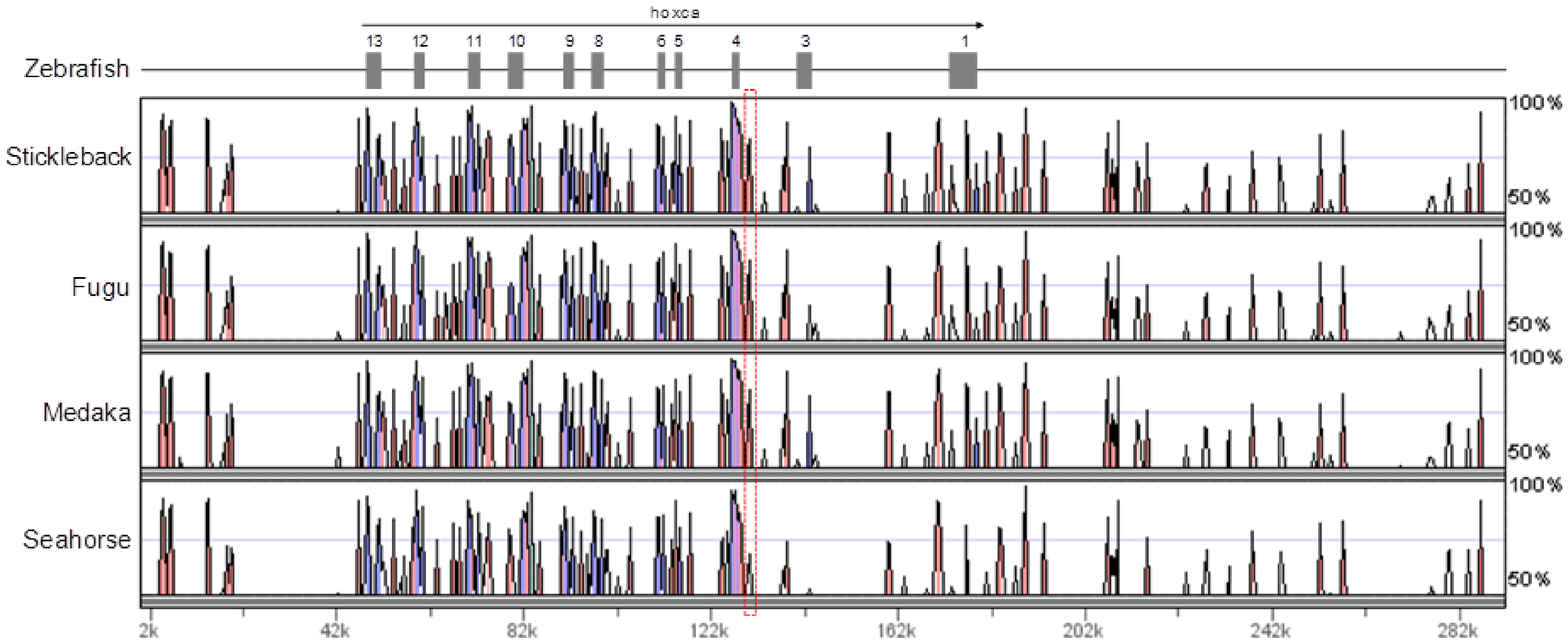

Supplementary Figure 6.2 Conserved noncoding elements (CNEs) in the HoxCa cluster of seahorse and other teleosts. Zebrafish is the reference sequence. Blue peaks represent conserved exons whereas pink peaks represent CNEs. The x-axis represents the base sequence whereas the y-axis denotes percentage identity. The red dotted box highlights CNE1 which is lost only in seahorse and was used for transgenic assay in zebrafish.

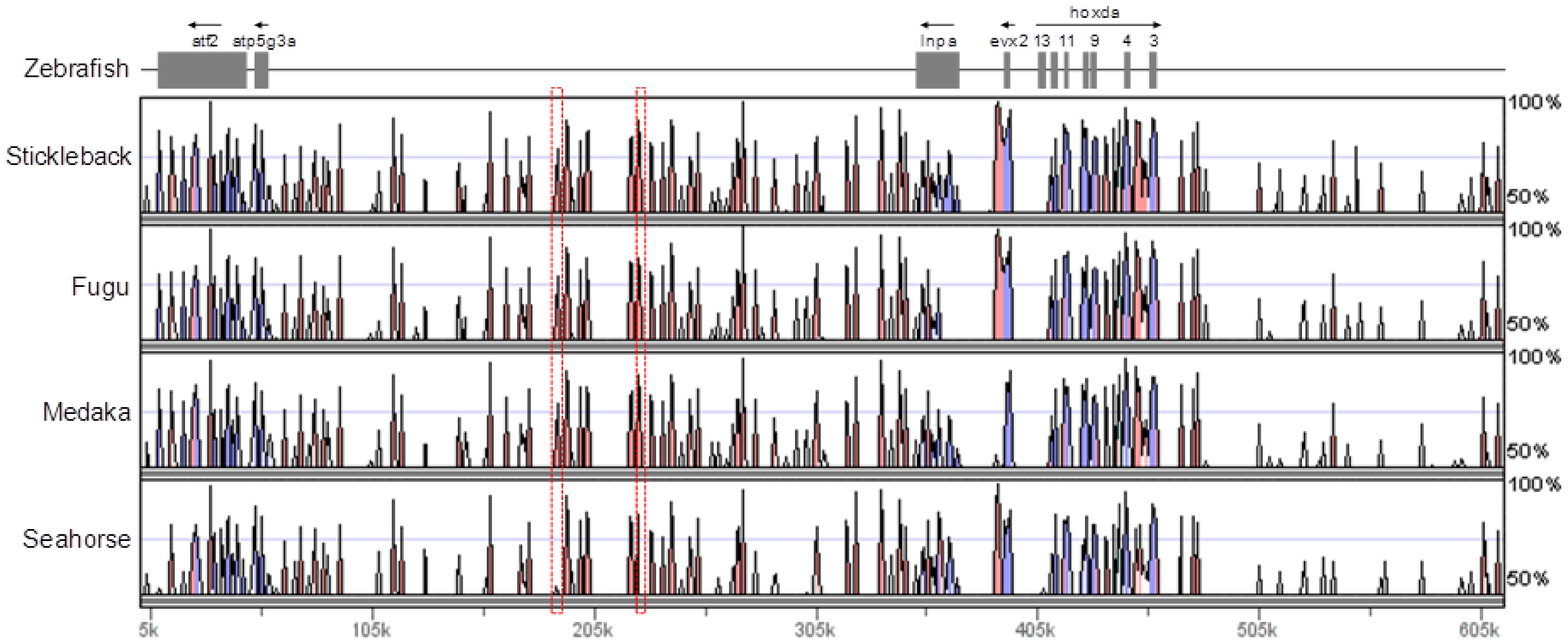

Supplementary Figure 6.3 Conserved noncoding elements (CNEs) in the HoxDa cluster of seahorse and other teleosts. Zebrafish is the reference sequence. Blue peaks represent conserved exons whereas pink peaks represent CNEs. The x-axis represents the base sequence whereas the y-axis denotes percentage identity. The red dotted boxes highlight CNE2 and CNE3 which are lost only in seahorse and were used for transgenic assay in zebrafish.

Supplementary Table 6.1 Statistics of conserved elements in the seahorse genome.

Type	Count	Total length (bp)	Shortest (bp)	Longest (bp)	Average (bp)	Median (bp)
Repetitive sequences	87,604	20,803,479	30	4,705	237	161
Exonic elements						
• Known genes (protein-coding or noncoding RNA)	278,271	54,001,002	30	9,938	194	144
• mRNA	2,533	491,931	30	2,054	194	130
• Spliced ESTs	26,399	5,749,652	30	3,348	218	145
• BLASTX hit in vertebrate Uniprot (E<1e-5)	3,497	1,131,307	56	3,638	324	223
Noncoding elements (CNEs)	239,976	40,323,876	30	2,860	168	113
All elements	638,280	122,501,247	30	9,938	192	135

Supplementary Table 6.2 Number and statistics of CNEs present in each of the percomorph fishes and in all five percomorph fishes plus zebrafish ("pan-teleost CNEs")

Class of CNEs	Count	Total length (Kb)	Average length (bp)	Median length (bp)
All CNEs	239,976	40,324	168	113
Present in Seahorse	80,857	13,814	171	114
Present in Fugu	69,623	11,998	172	116
Present in Stickleback	96,647	15,623	162	111
Present in Medaka	103,198	17,978	174	114
Present in Nile tilapia	135,573	23,250	171	117
Pan-teleost CNEs	17,744	3,496	197	134

Supplementary Table 6.3 Gap-free syntenic intervals in zebrafish and other teleost fish genomes. Gap-free syntenic intervals in 2 genomes were defined as contiguous (gap-free) genomic regions not exceeding 500 kb that are flanked by 2 orthologous alignments of at least 70% identity over 100 bp, and occurring in the same order and orientation in both genomes.

Genome	Count	Avg. size (bp)	Median size (bp)
Seahorse	54,240	2,373	1,137
Fugu	55,050	1,989	898
Stickleback	57,634	2,514	1,106
Medaka	45,208	1,636	946
Nile tilapia	65,296	2,592	1,270

Supplementary Table 6.4 Number of CNEs absent in one of the percomorph fish genomes. A CNE was deemed to be absent or missing in a percomorph genome if it was not detected in Multiz or BLASTN alignments (see Methods), and derived from gap-free syntenic intervals in the percomorph and zebrafish genomes.

Genome	CNEs not detected in Multiz or BLASTN alignments	CNEs derived from gap-free syntenic intervals
Seahorse	5,277	1,612
Fugu	3,797	1,050
Stickleback	4,456	843
Medaka	2,827	335
Nile tilapia	1,225	281

Supplementary Table 6.5 Top 20 statistically significant (FDR q-value < 0.05) GO biological process terms (pan-teleost CNEs)

Term Name	FDR Q-Val	Fold Enrichment	Foreground Regions	Background Regions	Foreground Gene Hits
midbrain-hindbrain boundary development	2.67E-82	4.83	206	577	5
midbrain development	6.57E-69	3.52	254	975	14
hindbrain formation	3.80E-65	6.52	120	249	2
cerebellum formation	3.80E-65	6.52	120	249	2
embryonic camera-type eye development	2.55E-61	3.44	234	921	12
embryonic camera-type eye morphogenesis	9.80E-59	4.00	183	618	7
neural tube patterning	2.92E-57	3.59	206	777	5
rostrocaudal neural tube patterning	2.92E-57	3.59	206	777	5
hindbrain development	5.99E-53	2.45	354	1955	19
peripheral nervous system development	8.25E-53	3.14	230	991	10
neural tube development	1.29E-52	2.54	327	1739	20
anterior/posterior pattern formation	3.75E-46	2.03	466	3098	36
vesicle-mediated transport	1.36E-42	2.04	429	2848	37
cerebellum morphogenesis	9.55E-41	3.98	128	435	3
cerebellum development	4.70E-40	3.93	128	441	3
hindbrain morphogenesis	1.10E-39	3.49	148	573	6
otic placode formation	2.02E-35	3.21	149	627	9
embryonic eye morphogenesis	3.87E-35	2.76	189	927	13
thyroid gland development	4.29E-35	3.66	123	455	4
homophilic cell adhesion	7.06E-35	2.05	345	2273	30

Supplementary Table 6.6. Top 20 statistically significant (FDR q-value < 0.05) GO molecular function terms (pan-teleost CNEs).

Term Name	FDR Q-Val	Fold Enrichment	Foreground Regions	Background Regions	Foreground Gene Hits
steroid hormone receptor activity	3.39E-99	2.71	549	2744	29
ligand-dependent nuclear receptor activity	3.39E-99	2.71	549	2744	29
O-methyltransferase activity	6.06E-31	3.79	104	371	5
protein transporter activity	3.94E-22	2.53	140	747	8
transcription activator activity	1.43E-21	2.42	149	832	15
alcohol dehydrogenase (NAD) activity	1.44E-14	7.19	25	47	1
hexokinase activity	4.64E-14	3.41	55	218	1
positive transcription elongation factor activity	2.17E-10	5.55	23	56	1
histone deacetylase binding	4.36E-10	4.36	29	90	1
thymidylate kinase activity	3.55E-09	3.56	34	129	1
nucleotide kinase activity	6.10E-09	2.56	56	296	3
asparagine-tRNA ligase activity	2.81E-07	4.37	21	65	1
carbohydrate kinase activity	4.44E-07	2.18	62	385	2
protein-L-isoaspartate (D-aspartate) O-methyltransferase activity	9.33E-07	3.38	27	108	1
phosphotransferase activity, phosphate group as acceptor	1.77563E-06	2.03	67	446	6
oxo-acid-lyase activity	1.86282E-06	3.94	21	72	2
transcription elongation regulator activity	2.23238E-06	3.09	29	127	3
flavin-containing monooxygenase activity	2.69563E-06	3.72	22	80	1
hydrogen ion transporting ATP synthase activity, rotational mechanism	3.2514E-06	2.55	39	207	5
aspartate-tRNA ligase activity	4.62863E-06	3.74	21	76	1

Supplementary Table 6.7 CNEs predicted in the Hox clusters of seahorse and other percomorph fishes (fugu, stickleback and medaka) using zebrafish as the reference sequence. CNEs that are uniquely lost in each of the percomorph teleost are also shown. Since zebrafish lacks the HoxDb cluster, stickleback was used as the base sequence in case of the HoxDb alignment (*).

Hox cluster	Number of CNEs present in				Number of CNEs lost specifically in			
	Seahorse	Fugu	Stickleback	Medaka	Seahorse	Fugu	Stickleback	Medaka
HoxAa	21	24	28	27	2	-	-	1
HoxAb	7	8	10	5	1	1	-	2
HoxBa	17	22	18	15	-	-	1	1
HoxBb	6	5	6	7	1	2	1	-
HoxCa	52	66	71	64	5	-	-	-
HoxDa	67	84	96	81	13	1	-	4
HoxDb	16	26	*	15	1	-	*	1
Total	186	235	229	214	23	4	2	9

Supplementary Table 6.8 Top 20 genes with the highest number of CNEs lost in seahorse.

No.	Gene name	Gene description	Total CNEs	Missing CNEs
1	*fto*	fat mass and obesity associated	210	33
2	*znf536*	zinc finger protein 536	401	23
3	*sall1a*	sal-like 1a	236	19
4	*C13H10orf11*	chromosome 10 open reading frame 11	384	19
5	*pax2a*	paired box gene 2a	229	17
6	*shox*	short stature homeobox	135	16
7	*sox14*	SRY-box containing gene 14	143	16
8	*nrf1*	nuclear respiratory factor 1	140	15
9	*lsm6*	LSM6 homolog, U6 small nuclear RNA associated	77	14
10	*irx5a*	iroquois homeobox protein 5a	318	14
11	*gpr18*	G protein-coupled receptor 18	93	13
12	*sox6*	SRY-box containing gene 6	268	13
13	*mms22l*	MMS22-like, DNA repair protein	70	12
14	*mef2aa*	myocyte enhancer factor 2aa	115	12
15	*zfhx3*	zinc finger homeobox 3	223	12
16	*nr2f1a*	nuclear receptor subfamily 2, group F, member 1a	321	12
17	*lmo1*	LIM domain only 1	76	11
18	*dachd*	dachshund d	124	11
19	*tox3*	TOX high mobility group box family member 3	178	11
20	*mab21l1*	mab-21-like 1	134	10

Supplementary Table 6.9 Top 20 genes with the highest percentage of CNEs lost in seahorse

No.	Gene name	Gene description	Total CNEs	Missing CNEs	Percentage of missing CNEs
1	*tmem101*	transmembrane protein 101	3	3	100.0%
2	*CABZ01041608.1*	Uncharacterized protein	1	1	100.0%
3	*hspb7*	heat shock protein family, alpha-crystallin-related, b7	3	2	66.7%
4	*slc18a3a*	solute carrier family 18 (vesicular acetylcholine), member 3a	3	2	66.7%
5	*shc1*	SHC (Src homology 2 domain containing) transforming protein 1	14	7	50.0%
6	*CABZ01039301.1*	Uncharacterized protein	6	3	50.0%
7	*tbpl1*	TBP-like 1	6	3	50.0%
8	*bpifcl*	BPI fold containing family C, like	2	1	50.0%
9	*CT583700.1*	Uncharacterized protein	2	1	50.0%
10	*CU928061.2*	Uncharacterized protein	2	1	50.0%
11	*GP9*	glycoprotein IX (platelet)	2	1	50.0%
12	*gpcpd1*	glycerophosphocholine phosphodiesterase GDE1 homolog	2	1	50.0%
13	*ndrg1b*	N-myc downstream regulated gene 1b	2	1	50.0%
14	*nrg2a*	neuregulin 2a	2	1	50.0%
15	*SHF*	Src homology 2 domain containing F	2	1	50.0%
16	*tbc1d31*	TBC1 domain family, member 31	2	1	50.0%
17	*wfs1a*	Wolfram syndrome 1a (wolframin)	2	1	50.0%
18	*chat*	choline acetyltransferase	7	3	42.9%
19	*ogdha*	oxoglutarate (alpha-ketoglutarate) dehydrogenase a (lipoamide)	7	3	42.9%
20	*hoxc12a*	homeobox C12a	10	4	40.0%

doi:10.1038/nature20595

Supplementary Table 6.10 Top 20 statistically significant (FDR q-value < 0.05) GO biological process terms (CNEs lost in seahorse).

Term Name	FDR Q-Val	Fold Enrichment	Foreground Regions	Background Regions	Foreground Gene Hits
regulation of transcription	2.33E-39	2.04	386	28113	124
regulation of cellular macromolecule biosynthetic process	2.91E-38	2.01	388	28737	126
regulation of macromolecule biosynthetic process	2.00E-38	2.01	388	28741	126
regulation of cellular biosynthetic process	2.91E-38	2.00	388	28826	126
regulation of biosynthetic process	2.80E-38	2.00	388	28850	126
regulation of transcription, DNA-dependent	2.87E-32	2.08	308	22073	99
regulation of RNA metabolic process	3.10E-32	2.07	309	22198	100
positive regulation of fibroblast growth factor receptor signaling pathway	1.12E-25	9.58	43	668	3
transcription	1.09E-21	2.13	205	14349	59
positive regulation of cell communication	4.61E-21	7.03	44	932	4
positive regulation of signaling pathway	4.61E-21	7.03	44	932	4
regulation of fibroblast growth factor receptor signaling pathway	1.28E-20	7.03	43	910	3
embryonic limb morphogenesis	6.40E-20	6.05	47	1157	6
embryonic forelimb morphogenesis	6.40E-20	6.05	47	1157	6
embryonic pectoral fin morphogenesis	6.40E-20	6.05	47	1157	6
embryonic appendage morphogenesis	1.22E-19	5.93	47	1179	6
limb morphogenesis	6.91E-19	5.67	47	1234	6
forelimb morphogenesis	6.91E-19	5.67	47	1234	6
pectoral fin morphogenesis	6.91E-19	5.67	47	1234	6
limb development	6.91E-19	5.67	47	1234	6

doi:10.1038/nature20595

Supplementary Table 6.11 Top 20 statistically significant (FDR q-value < 0.05) GO molecular function terms (CNEs lost in seahorse).

Term Name	FDR Q-Val	Fold Enrichment	Foreground Regions	Background Regions	Foreground Gene Hits
DNA binding	5.17E-37	2.03	371	27253	119
nucleic acid binding transcription factor activity	1.59E-35	2.25	290	19206	91
sequence-specific DNA binding transcription factor activity	1.59E-35	2.25	290	19206	91
sequence-specific DNA binding	8.38E-30	2.25	246	16293	76
transcription regulator activity	1.15E-16	2.06	176	12747	58
transcription activator activity	4.60E-08	4.65	26	832	5
steroid hormone receptor activity	2.35512E-06	2.55	47	2744	12
ligand-dependent nuclear receptor activity	2.35512E-06	2.55	47	2744	12
O-acetyltransferase activity	2.06527E-06	24.81	7	42	2
thymidylate kinase activity	3.83767E-05	10.39	9	129	1
nucleotide receptor activity, G-protein coupled	0.000113763	5.61	13	345	1
purinergic nucleotide receptor activity, G-protein coupled	0.000113763	5.61	13	345	1
purinergic nucleotide receptor activity	0.000219692	5.22	13	371	1
nucleotide receptor activity	0.000219692	5.22	13	371	1
choline O-acetyltransferase activity	0.000978391	63.80	3	7	1
purinergic receptor activity	0.000934493	4.46	13	434	1
histone methyltransferase activity (H4-R3 specific)	0.002937371	7.89	7	132	1
histone methyltransferase activity (H3-R2 specific)	0.002937371	7.89	7	132	1
histone methyltransferase activity (H2A-R3 specific)	0.002937371	7.89	7	132	1
histone-arginine N-methyltransferase activity	0.003090609	7.66	7	136	1

Supplementary Table 6.12 Expression pattern of zebrafish CNEs lost in seahorse.

S. No.	CNE	Length	Location	Expression in F1 embryos
1	#4452	186 bp	upstream of *shox*	no expression
2	#6440	106 bp	upstream of *sall1a*	no expression
3	#6560	194 bp	downstream of *sall1a*	forebrain, midbrain, hindbrain, floor plate and otic vesicle
4	#6500	263 bp	upstream of *lmo1*	forebrain, midbrain, hindbrain and spinal cord
5	#hoxc-1	272 bp	*hoxc4a-hoxc3a* intergenic	spinal cord
6	#hoxd-1	194 bp	*atp5g3a-lnpa* intergenic	melanocyte, heart and lens
7	#hoxd-2	236 bp	*atp5g3a-lnpa* intergenic	no expression

Four longest CNEs (#1 to #4) predicted by whole-genome comparisons and three three CNEs (#5 to #7) predicted by Hox loci comparisons were selected for enhancer assay in zebrafish. The positive CNEs generally seem to recapitulate part of the expression domains of their neighboring genes in zebrafish as described below: CNE #6560 is located downstream of *sall1a* which is known to express in the forebrain, midbrain, hindbrain and the otic vesicle besides other tissues (http://zfin.org/action/quicksearch/gene-expression/ZDB-GENE-020228-2). CNE #6500 is located upstream of *lmo1* which expresses in the forebrain, midbrain, hindbrain and spinal cord besides other regions (http://zfin.org/action/quicksearch/gene-expression/ZDB-GENE-021115-6). CNE #hoxc-1 is in the intergenic region of *hoxc4a* and *hoxc3a*. *hoxc4a* is known to express in the spinal cord besides several other tissues (http://zfin.org/action/quicksearch/gene-expression/ZDB-GENE-990415-112). CNE #hoxd-1 is flanked by *atp5g3a* and *lnpa*. *lnpa* is known to express in the mesenchyme pectoral fin. However, the expression pattern of zebrafish *atp5g3a* is not known. Thus, it is unclear whether the expression pattern of CNE #hoxd-1 resembles that of any of its neighboring genes.

7. Hox gene evolution

Hox genes in *H. comes*

Hox genes encode homeodomain-containing transcription factors that define segmental identities along the anterior-posterior axis of developing metazoan embryos[63]. In vertebrates, Hox genes are also critical for anterio-posterior as well as proximal-distal patterning of limbs[64]. As such, changes in Hox gene complement, sequence and regulation have been frequently suggested to underlie evolutionary transitions. The *H. comes* genome contains seven Hox clusters like most other teleost fishes, with a gene complement of 45 Hox genes similar to that of fugu (Supplementary Fig. 7.1). We noted that all *hoxc3* gene copies have been independently lost in seahorse, fugu and the European eel (which has both a *Hoxca* and a *Hoxcb* cluster but lacks both *hoxc3a and hoxc3b* genes) while all other teleosts such as the African butterflyfish (*Pantodon buchholzi*), zebrafish, medaka, stickleback and Nile tilapia have retained *hoxc3a* (Supplementary Fig. 7.1).

Selection pressure on Hox genes

Using the branch-site model implemented in the codeml program of PAML package[42], we identified positive Darwinian selection signals in three Hox genes: *Hoxa11b*, *Hoxa2b*, and *Hoxa4a* (P-value < 0.05, Supplementary Table 7.1). Interestingly, the adaptive changes in the Hoxa11 gene were shown to play an essential role for the evolution of pregnancy in mammals, which is able to up-regulate the expression of prolactin endometrial cells[65]. By comparing the sequences of Hoxa11b in seahorse and other species, we identified two amino-acid sites in seahorse were under positive selection in the first exon of Hoxa11b (P-value > 0.98, BEB test[66], Supplementary Table 7.1). Both sites were validated in *H. comes* using Sanger sequencing and the assembled RNA-seq reads from *Hippocampus erectus* (data not shown). To check whether the positively selected sites in seahorse Hoxa11b overlap with the sites that were under positive selection at the ancestral branch of placental mammals[65], we collected the HoxA11 sequences that cover a wide range of mammals (placental and non-placental), teleost fishes, and shark, reconstructed the ancestral sequences for all the nodes using PAML, and found the two positively selected sites in seahorse don't overlap with the ten positively selected sites at the ancestral branch of placental mammals. HoxA2a, a member of HoxA2 paralog group, plays an important role in the facial development of vertebrates[67,68]. HoxA2 is believed to pattern the second arch skeleton by suppressing transformation from neural crest cells to skeleton cell[67,69]; therefore, chondrogenesis develops only in areas that lack of the expression of HoxA2[70]. Furthermore, Donaldson and colleagues found that HoxA2 controls Wnt–β-catenin-signaling pathway during second branchial arch development in mouse using Chip-seq[71] technology. Knockout experiments show that HoxA4 leads to homeotic transformation of cervical vertebrae, especially from the third to the seventh cervical vertebrae in mouse[72]. Abnormal pectoral girdle and rib formation were also

observed in mouse with homozygous knock-out alleles[72].

Supplementary Table 7.1 Seahorse Hox genes under selection.

Gene name	No. fish species compared	ωf	LTR	P-value	Positively selected sites
HoxA1a	9	7.6164	0.159072	ns	NA
HoxA2a	8	9.7744	1.843094	ns	NA
HoxA2b	8	2.94085	4.054444	<0.05	NA
HoxA3a	8	10.0568	2.831354	ns	NA
HoxA4a	9	999	4.312072	<0.05	63 G*<br>199 S*
HoxA5a	9	1.0000	0	ns	NA
HoxA9a	9	1.0000	0	ns	NA
HoxA9b	9	4.4071	0.372238	ns	NA
HoxA10a	8	998.9023	3.530592	ns	NA
HoxA10b	9	4.4886	3.081746	ns	NA
HoxA11a	9	3.8670	0.446864	ns	NA
HoxA11b	8	512.5814	4.532186	<0.05	80 D*<br>149 Q*
HoxA13a	9	1.0000	0	ns	NA
HoxA13b	9	1.0000	0	ns	NA
HoxB1a	9	1.0000	0	ns	NA
HoxB1b	9	27.4103	1.047934	ns	NA
HoxB2a	9	1.8379	0.076254	ns	NA
HoxB3a	9	1.0000	0	ns	NA
HoxB3b	7	279.4952	3.458184	ns	NA
HoxB4a	9	1.0000	0	ns	NA
HoxB5a	9	1.2256	0.06865	ns	NA
HoxB5b	8	1.0000	0	ns	NA
HoxB6a	7	1.0000	0	ns	NA
HoxB8a	7	7.4851	1.113974	ns	NA
HoxB9a	7	1.0000	0	ns	NA
HoxB13a	9	1.0939	0.002668	ns	NA
HoxC4a	9	1.2516	0.03995	ns	NA
HoxC5a	9	1.0000	0	ns	NA
HoxC6a	9	51.2794	0.030106	ns	NA

HoxC8a	9	15.7861	2.112044	ns	NA
HoxC9a	9	5.9442	0	ns	NA
HoxC10a	9	1.0458	0.00267	ns	NA
HoxC11a	9	110.9551	3.658492	ns	NA
HoxC12a	9	1.0000	0	ns	NA
HoxC13a	9	1.0000	0	ns	NA
HoxD3a	8	2.5809	1.135544	ns	NA
HoxD4a	9	4.4886	3.081746	ns	NA
HoxD4b	7	1.0000	0	ns	NA
HoxD9a	9	104.6913	3.080332	ns	NA
HoxD9b	7	1.0000	0	ns	NA
HoxD10a	9	10.5715	0.48354	ns	NA
HoxD11a	9	1.5317	0.102358	ns	NA
HoxD11b	3	1.0000	0	ns	NA
HoxD12a	9	2.0472	0.072718	ns	NA

LTR: likelihood ratio test, ωf: selection pressure on foreground branch. P-value > 0.05 were listed as non-significant (ns) in the table. NA indicates no significantly positive selected sites were identifed by Codeml. * indicates P-value for positively selected sites is greater than 0.95.

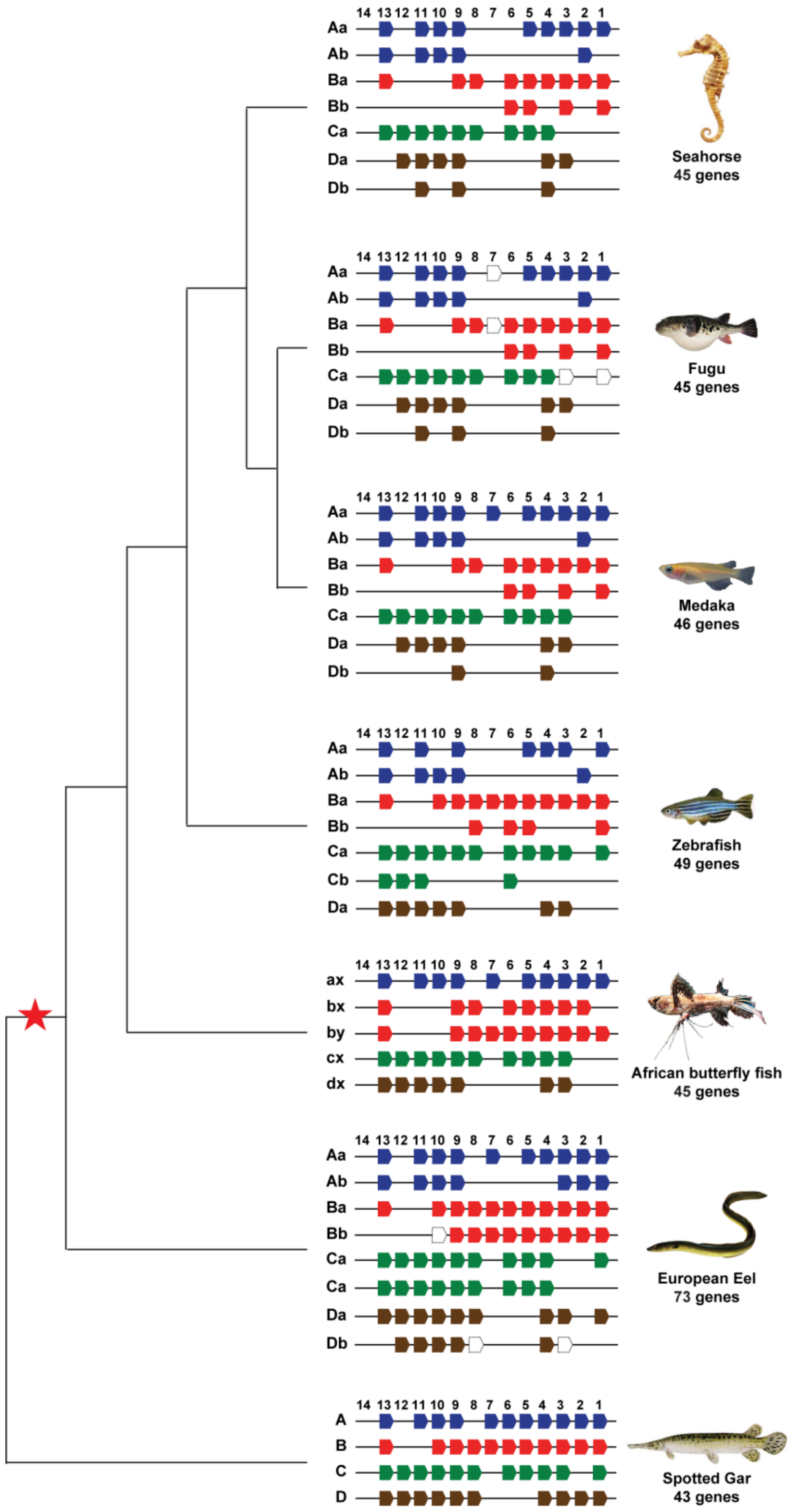

Supplementary Figure 7.1 | Hox gene clusters in tiger tail seahorse (*H. comes*) and other ray-finned fishes. Hox genes are shown as block arrows with the direction of the arrow representing the transcriptional orientation. Pseudogenes are shown as white dotted blocks. The red star represents the teleost-specific whole genome duplication event.

8. OR (olfactory receptors) genes in seahorse

The sense of smell is mediated by the detection of chemical odors by ORs (olfactory receptors). OR genes belong to the largest group of G protein-coupled receptors (GPCRs). Ligands for the rhodopsin-like GPCRs include photons, peptide hormones, odorants, etc. The functional OR genes identified can be separated into 2 major group, type1and type 2. Type 1 genes contain groups α, β, γ, δ, ε, ζ, and type 2 genes contain five groups that were named η, θ1, θ2, κ, and λ[73]. Group α and γ are responsible for airborne odorants, group δ, ε, ζ, η are responsible for water-soluble odorants, and group β may function in both airborne and water-soluble odorants[74].

We downloaded 1,417 protein sequences of classified OR gene family from NCBI and mapped to the seahorse genome using Tblastn with "E-value ≤1e-10" and "alignment rate ≥ 0.5". Solar (in-house software, version 0.9.6) was used to conjoin high-scoring segment pairs (HSPs) between each pair of protein mapping result. We did a filtered and only retained the results which were with alignment rate more than 70% and mapping identity more than 40%. Then, the protein sequences were placed on the genome using Genewise and extended 280bp upstream and downstream to define integrated gene models. Finally, we identified 26 OR genes in the seahorse genome. For phylogenetic analysis, protein sequences were aligned using MUSCLE and a JTT+gamma model was used in a maximum-likelihood analysis using PhyML to construct a phylogenetic tree.

In comparison to the OR repertoire (ranging from 60 to 169) of other teleost fishes, we only identified 26 OR genes in seahorse genome[73,75]. Most of the seahorse OR genes (18 genes) belong to the 'delta' group, which are involved in the perception of water-borne odorants[74]. The phylogenetic analysis of OR genes suggests that the lowest number of OR genes may resulted in either lack of lineage-specific expansion or massive gene loss in seahorse. These findings indicate that seahorse may rely less on sense of smell to find food or mating compared to other teleost fishes. In the closely related pipefish (*Syngnathus typhle*), smell alone was not a sufficient stimulus to discriminate between sexes[76].

9. Loss of tbx4 in seahorse and generation of a *tbx4* mutant zebrafish line

9.1 Evidence for loss of tbx4 in seahorse

The synteny analysis of *tbx2b-tbx4-brip1* region of seahorse, stickleback, fugu, and zebrafish using vista shows that *tbx4* was lost in seahorse (Figure 3). To exclude the scenario that the absence of *tbx4* in the seahorse genome sequence is due to an assembly error, we first validated the micro-synteny region of *tbx2b-tbx4-brip1* region in seahorse using a PCR- based genomic walk strategy. Briefly, 28 primer pairs (Supplementary Table 9.1) were designed for overlapping amplicons to 'walk' from the end of *tbx2b* to the start of *brip1*. Amplicon size and partial end sequencing of these products did not indicate any anomalies in the assembly of the seahorse *tbx4* 'ghost locus'.

In addition, we carried out the following analyses:

1. searched the *H. comes* genome (TblastN) using tbx4 protein from zebrafish and Nile tilapia and were unable to find a *tbx4* gene.
2. next searched *H. comes* genome using only the domain sequence of the tbx4 protein but were unable to find a *tbx4* gene.
3. searched *H. comes* and *Hippocampus erectus* transcriptome data for *tbx4* (TblastN) using tbx4 protein from zebrafish and Nile tilapia but were unable to find any matching transcript.
4. searched *H. comes* and *Hippocampus erectus* transcriptome data with the domain sequence as well and did not find any remnant of a *tbx4* gene.
5. predicted CNEs in the 'ghost' *tbx4* locus of seahorse using the fugu *tbx4* locus as the reference (base) (Supplementary Figure 9.3). We used the CNEs present in the other fish genome loci (that were absent in seahorse) to search the seahorse genome to rule out the possibility that they may be present elsewhere in the genome. We were unable to find any of these CNEs in the seahorse genome.

Finally, we conducted degenerate PCR experiments to ascertain if the *tbx4* gene is missing in seahorses. Using a combination of four forward and two reverse primers (Supplementary Table 9.1), we checked for the presence of *tbx4* in seven species of seahorse (genus *Hippocampus*, including *H. comes* and *H. erectus*), five species of pipefish (four from the genus *Syngnathus* and one species of *Corythoichthys*) (all from the family Syngnathidae that lack pelvic fins); ghost pipefish (*Solenostomus*) and the trumpetfish (Aulostomidae) which are closely related to the Syngnathidae but possess pelvic fins; and five other teleost species that possess pelvic fins (Supplementary Figures 9.1 and 9.2). While pelvic fin-containing teleost fishes were positive for the degenerate PCR, there was no PCR product in seahorses and pipefish (Supplementary Figures 9.2, upper panel). Sequencing of the PCR products from the pelvic fin-containing species confirmed that the amplified product was indeed from *tbx4* (Supplementary Figures 9.2, lower panel).

Together, these different lines of evidence strongly suggest that the seahorse genome does not possess the *tbx4* gene. This gene was most likely lost in the common ancestor of all syngnathids.

Supplementary Table 9.1: Degenerate PCR primer sequences for *tbx4* showing the forward (FW) and reverse (RV) primers used.

Primer name	Sequence
FW1	GAGGATGTTTCCTAGCTACAAGG
FW2	GAGGATGTTTCCTAGCTACAA
FW3	GAGRATGTTTCCWAGCTACAARG
FW4	GAGRATGTTTCCWAGCTACAA
RV1	TCRCWGAAYTTGTADCGRTGGTC
RV2	TCACAGAAYTTGTADCGGTGGTC

		RV1				RV2			
		FW1	FW2	FW3	FW4	FW1	FW2	FW3	FW4
	Astatotilapia burtoni	1	1	1	1	1	1	1	1
	Xenentodon cancila	1	1	1	1	1	1	1	1
	Aulostomus chinensis	1	1	1	0	1	1	1	1
	Antennarius striatus	1	1	1	1	1	1	1	1
	Synchiropus splendidus	0	1	0	1	0	1	0	1
	Amphiprion percula	1	1	1	1	1	1	1	1
	Solenostomus paradoxus	1	1	0	1	0	1	1	1
Syngnathidae	*Syngnathus rostellatus*	0	0	0	0	0	0	0	0
	Syngnathus biocellatus	0	0	0	0	0	0	0	0
	Syngnathus louisianae	0	0	0	0	0	0	0	0
	Syngnathus floridae	0	0	0	0	0	0	0	0
	Corythoichthys spec.	0	0	0	0	0	0	0	0
	Hippocampus zoestra	0	0	0	0	0	0	0	0
	Hippocampus comes	0	0	0	0	0	0	0	0
	Hippocampus subelongatus	0	0	0	0	0	0	0	0
	Hippocampus erectus	0	0	0	0	0	0	0	0
	Hippocampus kuda	0	0	0	0	0	0	0	0
	Hippocampus abdominalis	0	0	0	0	0	0	0	0
	Hippocampus barbouri	0	0	0	0	0	0	0	0

Supplementary Figure 9.1: Results of the degenerate PCR experiments with the complete set of species and the primer combinations used (1: PCR positive; 0: PCR negative)

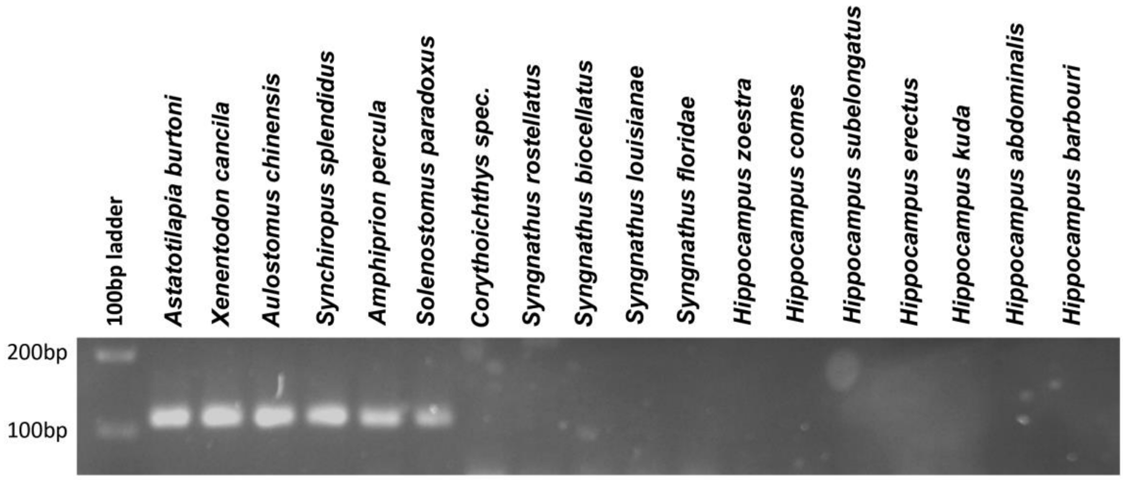


```
                          V  K  V  T  G  M  N  P  K  T  K  Y  I  L  L  I  D  I  V  P  A  D
Aulostomus chinensis      GTCAAAGTAACTGGGATGAACCCCAAAACTAAGTACATCCTATTAATTGATATTGTTCCAGCTGAT
Antennarius striatus      GTGAAGGTGACTGGCATGAACCCCAAAACCAAATACATCCTCCTCATTGACATCGTCCCAGCTGAT
Amphiprion percula        GTGAAGGTGACTGGCATGAACCCCAAAACCAAATACATCCTCCTCATTGACATCGTCCCAGCTGAT
Solenostomus paradoxus    GTCAAAGTGACAGGGATGAACCCCAAGACCAAATACATCCTCCTGATTGACATTGTCCCAGCTGAT
Synchiropus splendidus    GTAAAAGTGACTGGGATGAACCCCAAAACCAAATACATCCTCCTGATTGACATTGTCCCAGCTGAT
                          ** ** ** ** ** *********** ** ** ********  * ***** ** ** *********
```

Supplementary Figure 9.2: *tbx4* degenerate PCR results. Primers FW2 and RV1 which amplify a portion of *tbx4* exon 3 were used. These primers amplify the *tbx4* fragment in teleost fishes [*Astatotilapia burtoni* (cichlid fish), *Xenentodon cancila* (needlefish), *Aulostomus chinensis* (trumpetfish), *Synchiropus splendidus* (Mandarin fish), *Amphiprion percula* (clownfish), *Solenostomus paradoxus* (ghost pipefish)] that possess pelvic fins but not in pipefish (*Corythoichthys* and *Syngnathus* species) and seahorses (*Hippocampus* species) that lack pelvic fins.

9.2 Analysis of CNEs associated with the seahorse *tbx4* ghost locus

Alignment of the *tbx2b-brip1* region from selected vertebrates using LAGAN (Supplementary Figure 9.3) showed that the seahorse '*tbx4'* locus has lost most CNEs that are normally found in this locus in other teleosts (fugu, tilapia, medaka and stickleback), spotted gar and coelacanth (light green bars). Five CNEs usually located between *tbx2b* and *brip1* are, however, conserved in the seahorse locus (yellow bars) and may correspond to long-range regulatory elements involved in the regulation of *tbx2b* or another neighboring gene. The genomic region between *tbx4* and *brip1* is known to contain a hind limb-specific enhancer that is conserved as a CNE in tetrapods and teleosts (HLEB)[77,78] (blue bar). In seahorse, we cannot identify this region and it has likely been lost together with *tbx4*.

The presence of the five conserved CNEs (yellow bars) mark the original position of *tbx4* to a 14 kb region (shown as a dashed line), that is similar in size to the *tbx4* genomic region in other teleosts. Translated Blast searches using conserved domain sequences were unable to pick up any remnants of the *tbx4* coding regions. The

complete loss of conserved motifs is compatible with the loss of a functional *tbx4* gene in the seahorse.

Conserved noncoding elements from fugu that are present in other teleosts but not in the seahorse *tbx4* 'ghost locus' were BlastN searched against the seahorse genome to validate their genuine absence and exclude the possibility that they may have been erroneously assembled into another scaffold. We were unable to detect any such CNE in the seahorse genome.

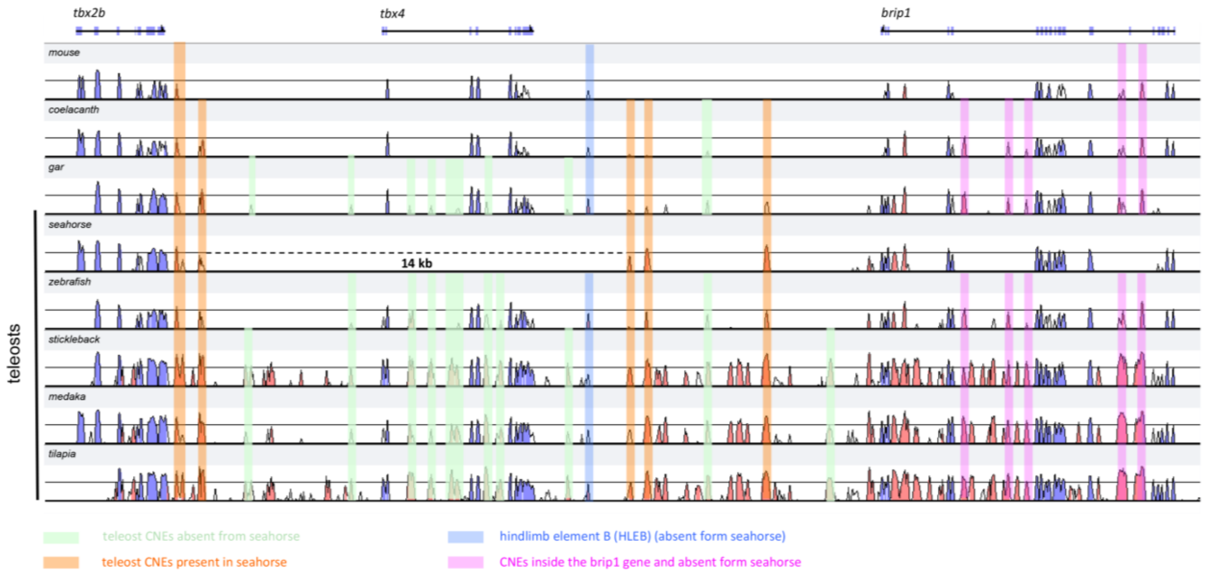

Supplementary Figure 9.3 VISTA plot of the *tbx2-tbx4-brip1* locus in seahorse, tilapia, medaka, stickleback, zebrafish, spotted gar, coelacanth and mouse. Fugu is the reference sequence. Conserved non-conding elements (CNEs) that are maintained in seahorse are indicated using orange bars, CNEs absent from seahorse are indicated with green bars, the hindlimb enhancer B (HLEB)[78] is indicated with a blue bar, CNEs present in the intronic regions of *brip1* but absent from seahorse are indicated with lilac bars. The seahorse synteny region shows not only loss of *tbx4* coding regions but also of teleost CNEs (green bars). Five CNEs still retained in seahorse (orange bars) mark a 14 kb region formerly containing the *tbx4* gene (indicated with a dashed line). In addition fish/vertebrate specific CNEs present in in the *brip1* intronic region have been lost from seahorse, indicative of a further regulatory erosion of the *tbx4* regulatory landscape.

9.3 Generation of mutant *tbx4* zebrafish and phenotypic analysis

To investigate the phenotypic consequences of *tbx4* loss in a teleost, we used a CRISPR/Cas9 strategy to generate a *tbx4* mutant zebrafish line. Two guide RNAs (gRNAs) were designed targeting zebrafish tbx4 in the 5' end of the sequence that is before or inside the DNA-binding TBOX domain (Supplementary Figure 9.4). gRNAs were cloned using synthesized oligos into the pT7gRNA vector as described in[79]

(oligos sequences given in Supplementary Table 9.2). gRNAs were synthesized from this vector using linearization with BamH1-HF (NEB R3136T), transcribed using the MEGAscript T7 Transcription Kit (Thermo Fischer Scientific AM1334) and purified using the mirVana miRNA isolation kit (Thermo Fischer Scientific AM1560). Cas9 mRNA was synthesized from the Cs2+Cas9 vector using the mMessage mMachine Sp6 Transcription Kit (Thermo Fischer Scientific AM1340) and purified using the RNA cleanup protocol from the RNAeasy mini kit (Qiagen 74104).

Zebrafish from a wild caught strain were injected at the one cell stage with ~50ng gRNA and ~90 ng Cas9 RNA. These F0 fish were raised to maturity and genotyped using fin clipping, DNA isolation and PCR spanning the target site (genotyping primers given in Supplementary Table 9.2). PCR products were analyzed for mutations as described[79] using T7 endonuclease (NEB M0302L). Mosaic mutant F0 fish were outcrossed to AB wildtype fish and embryos were batch genotyped for transmission of the mutation using PCR and T7 endonuclease. Mutant PCR products were cloned into the pGEMT vector (Promega A3600) and sequenced to identify carrier fish transmitting a frameshift mutation. These carrier fish were crossed again to AB WT and the resulting F1 fish were raised to maturity. The F1 were phenotyped using using fin clipping, DNA isolation, PCR, T7 endonuclease to identify heterozygous mutant fish followed by cloning and sequencing of the mutant PCR products to validate presence of the frameshift allele. The CRISPR/Cas9 mutation strategy is schematically shown in Extended Data Fig.5.

In the F0 mutant tbx4 fish we observed pelvic fin loss at low frequency gRNA#1 gave 3/42 fish with either double or single sided pelvic fin loss and 1/34 had single sided pelvic fin loss for gRNA#2 (Extended Data Fig.5). We observed mutant allele transmission for both gRNA#1 and gRNA#2 but failed to identify a deletion leading to a frameshift mutation for gRNA#2 so no stable line was generated for this CRISPR. For gRNA#1 we identified several frameshift mutants, one of which was further analyzed. This mutant has a deletion/replacement mutation in which 8 nucleotides are replaced by 3 nucleotides, leading to an effective 5 bp deletion and the introduction of a frameshift mutation (Extended Data Fig.5). This mutation introduces a downstream STOP codon leading to a severely truncated protein lacking the DNA binding domain (Supplementary Figure 9.4 and 9.5). The mutant line is maintained on an AB WT background. The phenotype shown for the homozygote mutant in the main text is a representative F3 (one out of five homozygote) animal.

The *tbx4* mutant generated by us differs from two recently reported mutants by Don et al.[80]. One natural occurring mutant has an inactivation of the nuclear localization signal, while the other contains a TALEN induced mutation and has a premature stop codon at codon position 164. The *tbx4* mutant generated by us is predicted to retain 17 aa of the original *tbx4* coding sequence followed by 4 aa from out of frame translation

and hence results in a 21 aa peptide. As no part of the tbox domain is translated we infer that the chances for a dominant negative effect of a partial protein can virtually be ruled out for our mutant and our mutant is a true null mutation without confounding influences from partially translated functional domains.

In addition to the absence of pelvic fins and the pelvic girdle (as also described by Don et al.[80]) we observe abnormalities in the musculature of the ventral body wall in adult *tbx4* homozygous mutant fish (Supplementary Figure 9.6 a, b). Externally this phenotype is apparent as absence of the musculature normally associated with the pelvic girdle (PvFm in Supplementary Figure 9.6 a), but also more anteriorly where a clearly defined *musculus infracarinalis anterior*[80,81] that typically runs along the ventral midline, is absent. In contrast to wildtype fish, mutant fish show a somewhat translucent groove along their belly where the viscera shine through (Supplementary Figure 9.6 a). Sectioning shows that indeed these mutant fish have an opening in their ventral body wall musculature and either absence or very strong morphological abnormality of the *m. infracarinalis anterior* (Supplementary Figure 9.6 b).

We investigated expression of *tbx4* in the developing cichlid fish *Astatotilapia burtoni* where the embryonal stages during which body wall formation takes place are tractable using *in situ* hybridization (Supplementary Figure 9.6 c). *tbx4* is expressed in a domain much wider than the pelvic fins and is also strongly expressed in the muscle cells that will migrate to form the ventral midline. This expression domain is consistent with the observed phenotypic defects in the zebrafish mutant.

How this additional *tbx4* phenotype relates to the absence of *tbx4* in *Hippocampus* or pelvic fin loss in general is not directly clear, but we can make two interesting observations. First, despite the loss of their pelvic fin in pufferfish and eels, these species still maintain a functional *tbx4* copy in their genome, suggesting the presence of a pleiotropic constraint on loss of this gene.

We are not aware of any studies describing the muscular organization of the ventral body wall in any of these species, but it appears plausible that the altered muscular organization induced upon *tbx4* loss contributes to this constraint and explains why *tbx4* is retained in these species. Second, like all syngnathids, seahorses are covered in an armor of bony plates that may well mitigate the adverse effects of defects in body wall closure. Also, syngnathids and more specifically seahorses are known to have a highly derived hypaxial and ventral muscular system[82] and it is enticing to speculate that absence of *tbx4* may have contributed to its evolution.

doi:10.1038/nature20595

Supplementary Table 9.2 Primer sequences used to amplify the seahorse tbx4 synteny region for validation of the assembly. Each amplicon overlaps with the subsequent amplicon, thereby demonstrating physical linkage of the DNA sequences.

FW primer	RV primer
TTGGGATGTCTTGGCTTGCAGAC	TTTAAGCTCCCACTGTTGAAACCTC
GTGCTTTTACATATAGGGTAGATC	GCAGTTTGTGATCAGAGTTGTTG
CAACAACTCTGATCACAAACTGC	CCTGAGACCGATTCACACAAC
GTTGTGTGAATCGGTCTCAGG	CTGCTGTCCTGAAAATGCAGT
ACTGCATTTTCAGGACAGCAG	GGTTTGCCCGATAGAAACAGTAG
CTACTGTTTCTATCGGGCAAACC	CAACCCTGGAACAGTATTACAGT
ACTGTAATACTGTTCCAGGGTTG	GTGAATGCTGTCTGTTGCTAG
CCTTACGCTAACTGGAGCGCTAATG	AGCCTCACAGTCCAGAGCTGCAG
CACGTCAACCAGTCAACCACCATG	TCGAGTGCAGGACCTGATCAAC
CAACACTTGACCATCAGTATTTAATC	TTGTCAGTCATTTTTGCCACTG
CAGTGGCAAAAATGACTGACAA	TTATGAGGCGCAATCTACAATAC
GTATTGTAGATTGCGCCTCATAA	TTCCTTCATGTTTGTCCCTGAG
CTCAGGGACAAACATGAAGGAA	GCTTTGCGTTTCATGTCTCGTA
TACGAGACATGAAACGCAAAGC	CATGCCTGAGGAAGTTACTGTG
CACAGTAACTTCCTCAGGCATG	CTCTGCACTGTGAGGCCAATG
TGGTTGGTGCATTGGCCTCACAG	TAATGCTTACAAATGAGCTCTTTCAAC
CATCCTCCATAGTGGAAAAGTTG	GAGCTCTTTCAACCGGTCTCT
AGAGACCGGTTGAAAGAGCTC	TCCGACTGCAACGGACAGTTAG
CTAACTGTCCGTTGCAGTCGGA	GATGCAAGGCTTTCTCAAAGTCAAC
GACGCAGTTCCAAATAACGTG	GTCTCCGCGTCAGGTCTCTAC
CAAATTACTGCCTACCTTAGTAG	AATGAGGTGCAATTCATGGAATATC
CTCTGATCAAAACAGTGTGTCG	GATGCCTGAGCAACTAGTACGTC
GACGTACTAGTTGCTCAGGCATC	CAAGTGCATTTTTACCTTGACCTTCC
CTGGTAACCAGTTCAGGGTGTAC:	CAGCTGTTGGAACTTGGCTTGAG
CTCAAGCCAAGTTCCAACAGCTG	CTGGTTAGGATGCCTCCTGGATG
CATCCAGGAGGCATCCTAACCAG	GAGCCGTACACAATTATCACTGTG
CAGAGCCTTGAGAAACTCCGT	CCTTTGCAGAGGTGTCTCTTGTTAC
GTAACAAGAGACACCTCTGCAAAGG	TCTGAGGTCGCCCAAGATTCTCC

doi:10.1038/nature20595

Supplementary Table 9.2 Oligo sequences used for gRNA cloning and genotyping of the CRISPR/Cas9 induced mutations.

oligo name	oligo sequence	oligo purpose
dre-tbx4 gRNA#1 FW	TAGGAATGACCGTTGCCCAGTC	cloning gRNA
dre-tbx4 gRNA#1 RV	AAACGACTGGGCAACGGTCATT	cloning gRNA
dre-tbx4 gRNA#1 gt FW	TCTCTTTTTGAAAGTTGTACTTCCA	genotyping PCR
dre-tbx4 gRNA#1 gt RV	CTGGAAGCGAGATGGCCTAG	genotyping PCR
dre-tbx4 gRNA#2 FW	TAGGGTGGACATAGAGTCTTCC	cloning gRNA
dre-tbx4 gRNA#2 RV	AAACGGAAGACTCTATGTCCAC	cloning gRNA
dre-tbx4 gRNA#2 gt FW	ATGTCAGAGCTGCTGGAAAGCT	genotyping PCR
dre-tbx4 gRNA#2 gt RV	TTGCTCACCTTTAATGCCAAAG	genotyping PCR

doi:10.1038/nature20595

gRNA#1 target sequence: GGAATGACCGTTGCCCAGTCGGG (nt 37-59)
gRNA#2 target sequence: CCTGGAAGACTCTATGTCCACCC (nt 391-413)

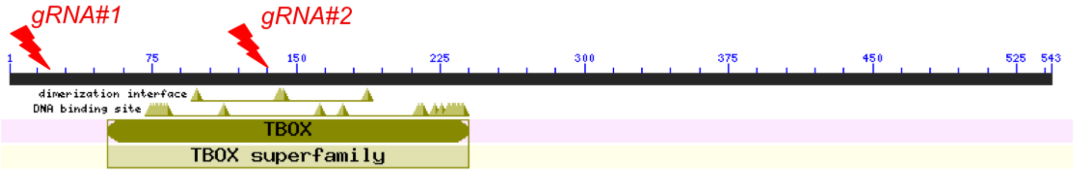

Supplementary Figure 9.4 *Tbx4* gRNA design. Two gRNAs were designed for target sites located in the 5' end of the *tbx4* coding sequence. gRNA#1 targets the first coding exon (exon 2) before the DNA-binding TBOX domain. gRNA#2 has its target site inside the TBOX domain in exon 5. Both gRNAs are expected to generate loss of function alleles because both target sites contain downstream out of frame STOP codons that will become functional upon -1 and -2 frameshift deletions (i.e. deletions of any number of nucleotides not divisible by 3). The position of the two gRNAs is shown on a schematic diagram of the protein sequence generated by the NCBI conserved domain database (CDD) in which the position of the TBOX domain is indicated.

```
Zebrafish_TBX4          MLQEKASVVADEGMTVAQSGGRPELASDSSHLGLPTTPSNPQNNEPDQSIENIKVVLHDR
Zebrafish_TBX4_mutant   MLQEKASVVADEGMTVAHSAS---------------------------------------
                        *****************:*..

Zebrafish_TBX4          ELWKKFHEAGTEMIITKAGRRMFPSYKVKVTGMNPKTKYILLTDIVPADDHRYKFCDNKW
Zebrafish_TBX4_mutant   ------------------------------------------------------------

Zebrafish_TBX4          MVAGKAEPAMPGRLYVHPDSPATGAHWMRQLVSFQKLKLTNNHLDPFGHIILNSMHKYQP
Zebrafish_TBX4_mutant   ------------------------------------------------------------

Zebrafish_TBX4          RLHIVKADENNAFGSKNTAYCTHVFHETAFISVTSYQNHKITQLKIENNPFAKGFRGSDE
Zebrafish_TBX4_mutant   ------------------------------------------------------------

Zebrafish_TBX4          GDLRVSRLQGKDYPVISKNMVRQRLISSHGHLSGKLSAGVLSSHPQVLSHYQYDSGVPLP
Zebrafish_TBX4_mutant   ------------------------------------------------------------

Zebrafish_TBX4          NSDSQEALSNSFTSSREPSLLYHCFKHRDNPRHLELGCKRPYLDTTSSAVSEEHYFRSPP
Zebrafish_TBX4_mutant   ------------------------------------------------------------

Zebrafish_TBX4          SYDSPLLSHPYCNEALGSREACMYGGLEGEGGGAVGTDDLPAPSLNCNMWASVQPYPRYG
Zebrafish_TBX4_mutant   ------------------------------------------------------------

Zebrafish_TBX4          MQTVEAMQYQPFTAHFNSTASAASMVSHHSPSMQRPHPTPPDLVTFTTQRVLPPTPSSAS
Zebrafish_TBX4_mutant   ------------------------------------------------------------

Zebrafish_TBX4          TSPSGSGHHDRAHSSLFHRKAGSPLRSQRDFTGYSTHSPTPTREPAYQYQTGLSSVGPHW
Zebrafish_TBX4_mutant   ------------------------------------------------------------

Zebrafish_TBX4          TDS
Zebrafish_TBX4_mutant   ---
```

Supplementary Figure 9.5 Alignment of wildtype and mutant zebrafish TBX4 proteins: Wildtype zebrafish TBX4 consists of 543 amino acids while the premature stop codon in the mutant allele reduces this to a predicted 21 amino acids. The mutant allele completely lacks the DNA-binding TBOX domain (highlighted in orange in the wildtype allele).

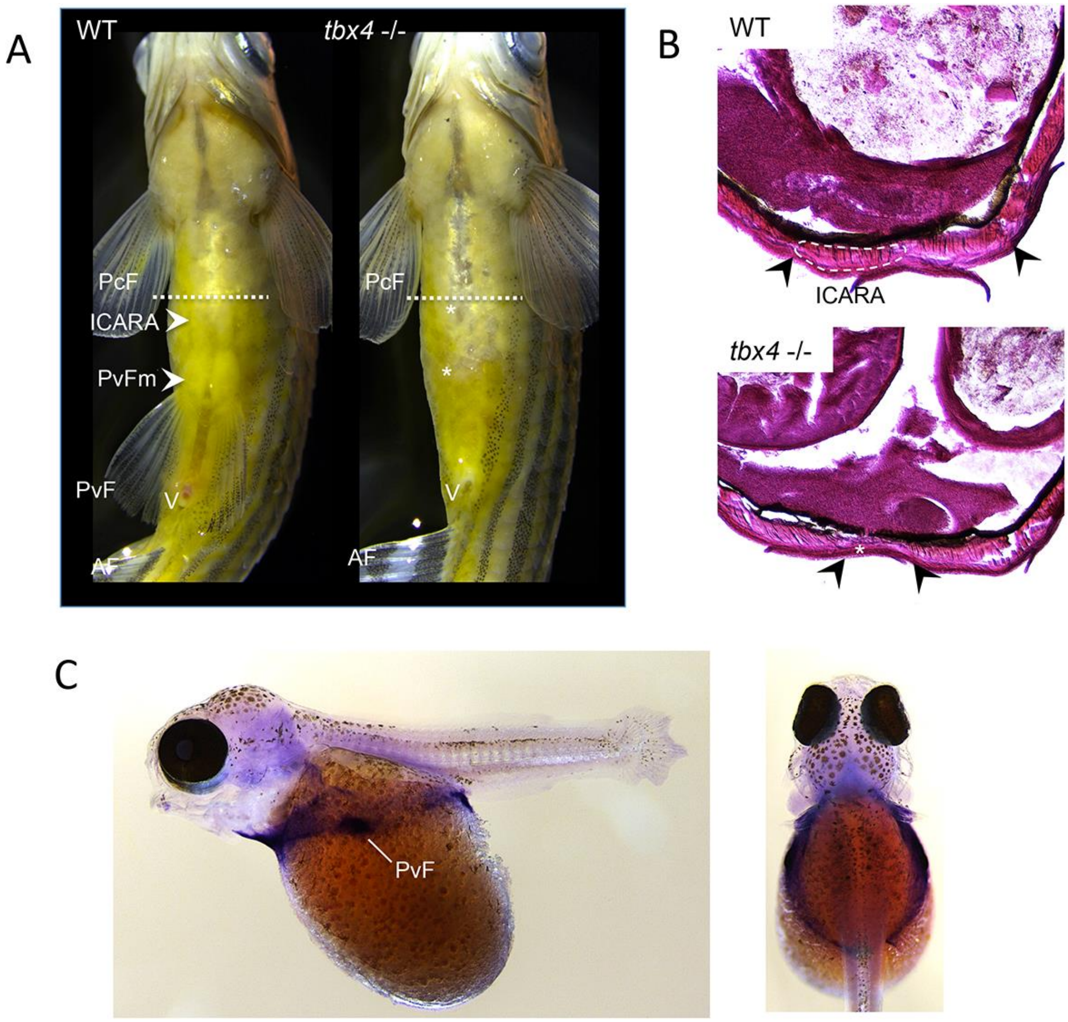

Supplementary Figure 9.6 Ventral body wall defects in tbx4 mutant zebrafish. **a)** ventral view of wildtype and tbx4 mutant fish. Wildtype fish show a distinct pattern of ventral musculature with pronounced bulges at the position of the pelvic girdle (pelvic fin musculature; PvFm) and more anteriorly the *musculus infracarinalis anterior* (ICARA). In *tbx4* mutant fish these muscles are not observed and these fish show a slightly translucent groove along their midline indicative of defects in the formation of their body wall (muscle absence indicated with asterisks). **b**) Cryosectioning (section shown approximately at the position of the dashed lines indicated in panel **a** of this figure) shows absence or strong malformation of the *musculus infracarinalis anterior* in the mutant leaving a gap in the body wall (*musculus infracarinalis anterior* indicated with a dashed outline in the wildtype). Black arrowheads indicate the ventral position of the hypaxial muscles. **c**) *In situ* hybridization for *tbx4* in embryos of a cichlid fish (*Astatotilapia burtoni*) - left panel lateral view, right panel dorsal view. *Tbx4* shows expression in a much wider domain than the pelvic fin (indicated PvF) and is also expressed in a ring around the embryo in cells that will contribute to the future ventral midline. This wider expression domain is consistent

doi:10.1038/nature20595

with the phenotype observed in the *tbx4* mutant fish. Abbreviations: Pcf; pectoral fin, PvF; pelvic fin, PvFm; pelvic fin musculature, AF; anal fin, ICARA; *musculus infracarinalis anterior*.

10. Description and analyses of specific gene families

10.1 Secretory calcium-binding phosphoprotein (SCPP) gene family

The secretory calcium-binding phosphoprotein (SCPP) genes encode extracellular matrix (ECM) proteins that are involved in the formation of mineralized tissues such as bone, dentin, enamel and enameloid. SCPP genes have been identified in the genomes of bony vertebrates (Osteichthyes) but are absent in the genomes of cartilaginous fishes (Chondrichthyes) like elephant shark (*Callorhinchus milii*). These genes arose through tandem duplication of the *SPARCL1* (*SPARC-like 1*)gene that was itself derived from the *SPARC* (*secreted protein, acidic and rich in cysteine*)gene through whole-genome duplication around the time that mineralized skeleton arose in vertebrates[83].

SCPP genes are divided into two groups: the acidic SCPP genes that are enriched with Asp, Glu and phospho-Ser (pSer) residues, and the proline/glutamine (P/Q)-rich SCPP genes. The acidic SCPPs regulate the mineralization of collagen scaffoldings in bone and dentin through association with calcium phosphate. In mammals, a class of acidic SCPP genes known as the SIBLING genes, comprising *DSPP*, *DMP1*, *IBSP*, *MEPE* and *SPP1* (Fig. 1), are important for bone and dentin formation. In particular, *SPP1* is predominantly expressed in bone matrix while *DSPP* is most highly expressed in dentin matrix[84]. In teleosts, the only SIBLING gene present is *SPP1* but there exist teleost-specific acidic SCPP genes such as *scpp1*, *scpp8* and *gps37* in zebrafish (Extended Data Fig. 3). Knockout of zebrafish *SPP1* results in significant reduction of dermal and endochondral bone formation in embryos[85].

The P/Q-rich SCPPs are primarily involved in enamel formation. During the secretory stage of enamel formation, P/Q-rich enamel matrix protein (EMP) genes (*AMEL*, *AMBN* and *ENAM*) are expressed in ameloblasts and the proteins are secreted into the ECM. Mammals have EMP genes *AMEL*, *AMBN* and *ENAM*, the hypermineralization genes *ODAM*, *AMTN* and *SCPPPQ1* and a whole host of milk casein and salivary protein P/Q-rich SCPP genes. In teleost fishes, none of the EMP genes has been found thus far, presumably because EMPs may not be required for teleost enameloid whose formation is more similar to that of dentin than enamel. In contrast to tetrapod enamel that forms in a noncollagenous ECM deposited by ameloblasts, teleost enameloid forms in a dentin-like collagenous ECM that is deposited by both inner dental epithelial cells and odontoblasts[86]. To achieve this, teleost fishes use *SCPP2* (the orthologue of tetrapod *ODAM*) and a set of teleost-specific P/Q-rich SCPP genes. For example, in zebrafish, *scpp5* is expressed strongly in both inner dental epithelial cells and odontoblasts when the enameloid and dentin matrix are being deposited, suggesting that it is involved in enameloid and dentin matrix formation. In addition, zebrafish *scpp2* and *scpp9* are expressed strongly in only the inner dental epithelial cells at the maturation stage of enameloid after the enameloid has been fully

mineralized, pointing to the role of *scpp2* and *scpp9* as hypermineralization genes [87].

The seahorse contains only two SCPP genes – the acidic *SCPP1* and *SPP1* (Extended Data Fig. 3). Full-length transcripts for both genes are found in the combined tissue transcriptome of *Hippocampus erectus*. The seahorse *SCPP1* locus contains a pseudogene for the P/Q-rich *SCPP5* gene which is represented by only three exons, despite the fact that the sequence in this genomic region is gap-free. Moreover, no transcript for this gene could be found in the combined tissue transcriptome of *Hippocampus erectus*. Thus, the seahorse possesses only two acidic SCPP genes and no P/Q rich SCPP gene (Extended Data Fig. 3). Other teleosts such as fugu and zebrafish possess eight and nine P/Q-rich SCPP genes, respectively, whereas medaka contains three P/Q-rich SCPP genes.

Seahorses are toothless, a phenomenon known as edentulism. The only other teleost fishes known to lack teeth are pipefish and the adult sturgeon. Edentulism is believed to have occurred independently in several jawed vertebrate lineages[88], the most notable ones being birds[89], turtles and certain mammalian lineages including baleen whales, pangolins and anteaters[90]. In the genomes of modern birds (Neornithes), some turtles (Western painted turtle, green sea turtle and Chinese soft-shelled turtle) and some toothless mammals (nine-banded armadillo, Hoffmann's two-toed sloth, aardvark and Chinese pangolin), the enamel-specific genes, *ENAM*, *AMEL*, *AMBN*, *MMP20* and *AMTN*, and the dentin-specific gene, *DSPP*, were found to possess inactivating mutations, and were correlated with tooth loss[89,91]. In teleost fishes, the P/Q-rich SCPP genes perform functions analogous to these genes. Since P/Q-rich SCPP genes are critical for enamel formation and hyper-mineralization, the complete loss of such SCPP genes in seahorse might explain their lack of teeth.

Methods

SCPP and related genes were identified in the seahorse genome and transcriptome by TBLASTN using representative proteins. Identified regions of homology were used for BLASTX searches to confirm the identity of the genomic region. Predictions were also made using *ab initio* methods such as FGENESH[92] when no homology was obtained by BLAST-based approaches. All predictions were checked and refined manually.

10.2 The evolution of astacin metalloproteinase gene family

Phylogenetic analysis of *astacin* family

The protein sequences of the seahorse astacin family that were predicted in this study were extracted and manually curated. The protein sequences of the *astacin* family in zebrafish, medaka, stickleback, tilapia, *Tetraodon*, platy fish and spotted gar were filtered and downloaded by Biomart from Ensembl database (Ensembl Genes 82). The Biomart filtered the protein sequences which have the *astacin* protein domain (IPR001506). To avoid the gene overlapping, the longest one was chosen and the short ones which have the same gene ID were removed. The protein sequences of C6ASTs in *astacin* family which have conserved six cysteines were aligned using the online version Muscle [93] (http://www.ebi.ac.uk/Tools/msa/muscle/). The phylogeny tree was constructed using PhyML software [94] with the model of "WAG +gamma". Chromosomal locations of astacin genes in the genome of these species were identified using the UCSC Genome Browser (http://genome.ucsc.edu/). The expression pattern of the C6AST genes in seahorse was analyzed by Heatmap.

To identify the evolution of patristacin in seahorse, we reconstructed a phylogeny tree of C6ASTs in the *astacin* family from eight teleost genomes (seahorse, zebrafish, medaka, Nile tilapia, sticklebacks, green spotted puffer, platyfish and spotted gar) (Fig. 6a). In the phylogeny tree (Supplementary Figure 10.1), there are five subfamilies clustered in this family, including Patristacin/Astacin, Nephrosin, Choriolysin (HCE and LCE), HCE1-like and HCE2-like. Phylogenetic analysis found clustering of patristacin genes to themselves, suggesting that the expansion is specific to the seahorse and the pipefish. In the meanwhile, *astacin* genes of platy fish also clustered together to construct one specific cluster. And these two clusters evolved independently.

Positive selection and 3D structure of patristacin genes in seahorse (*H. comes*)

The coding and protein sequences of the seahorse *astacin* family that were predicted in this study were extracted and manually curated. The coding and protein sequences of the *astacin* family in spotted gar, zebrafish, medaka, stickleback, tilapia, tetraodon, and platyfish were downloaded from the Ensembl database (Ensembl Genes 82). The patristacin sequence of pipefish was extracted from transcriptome of *Syngnathus scovelli*[31] using tblastx method. To generate high quality alignment for further analyses, we first aligned the protein sequences of astacin family using the online version Muscle[93] (http://www.ebi.ac.uk/Tools/msa/muscle/), filtered out the poorly alined regions and saturated sites using trimal[35] version 1.4. We then aligned the coding sequences of *astacin* family using the protein alignment as a guide. The selection pressures of *patristacin* genes in seahorse were examined using the branch-site model[42] by codeml in the PAML toolkit[43] (version 4.4). To avoid local optima in codeml, we specified three initial omega values in each run.

In total, 78 genes of *astacin* family were included in the analysis (Supplementary

Table 10.1). After filtering the saturated sites and poorly aligned regions, 579 bp in the coding regions were used in the selection pressure analysis. Using the branch-site model, a significant selection signal was identified on the *pastn*3 copy in seahorse. Besides, one codon (Position 45, Threonine-> Arginine, corresponding to the amino acid 126 on the un-trimmed *pastn3)* on the *pastn*3 copy was positively selected (Supplementary Table 10.2) based on the Bayes Empirical Bayes method [66].
3D structures of Patristacin proteins were modeled via the Swiss-Model homology modeling server (http://swissmodel.expasy.org) with the Zymogen structure of crayfish *astacin* metallopeptidase as a template. The result showed that Arg[126] of *pastn*3 was positive selected.

Expression profile of patristacin genes in seahorse (*Hippocampus erectus*)
The expression pattern of *patristacin* was examined in eight tissues of seahorse, including brain, gill, intestine, kidney, liver, muscle, brood pouch, ovary, and testis using quantitative Reverse Transcription PCR (qRT-PCR) with five biological replicates in each test. In addition, we also compared the expression of *patristacin* between different pregnant (early and late stage) with that of non-pregnant stages. The qRT-PCR was performed using the Light Cycler®480 Sequence Detection System (Roche, Switzerland) with a 384-well plate. Each reaction involved a 3 min denaturation step at 94 °C, then 40 cycles of 94 °C for 15 s, 52 °C for 15 s, 72 °C for 30 s, and 85 °C for 1 s to collect fluorescent signals, then a stepwise temperature increase from 52 °C to 99 °C to establish the melting curve. Standard curves were constructed using a series of 10-fold dilutions of quantified pMD®18-T Simple vector (TaKaRa, China) for each of the target genes. For all target genes, 18S rRNA was used as the reference gene. All the data were expressed as mean ± standard error of mean (S.E.M) and evaluated by one-way analysis of variance (ANOVA) followed by the Duncan's multiple-range tests. Results were considered to be statistically significant when $P\text{-value} < 0.05$. All statistical analyses were carried out using SPSS for Windows, Version 20 (SPSS, Chicago, IL, USA).

Using real-time PCR technique, the expressions of *pastn* genes were investigated seahorse pregnancy. We found the expressions of *pastn* genes are very dynamic during seahorse pregnancy.
Real-time PCR showed that *pastn*3 and *pastn*5 were widely expressed in female and male seahorse peripheral tissues, and *pastn*1, *pastn*2 and *pastn*6 were only expressed in female ovary and male brood pouch (Supplementary Figure 10.2). Overall, an increased expression of patristacin genes was observed in brood pouch of male seahorse and the ovary of female seahorse than in other tissues.

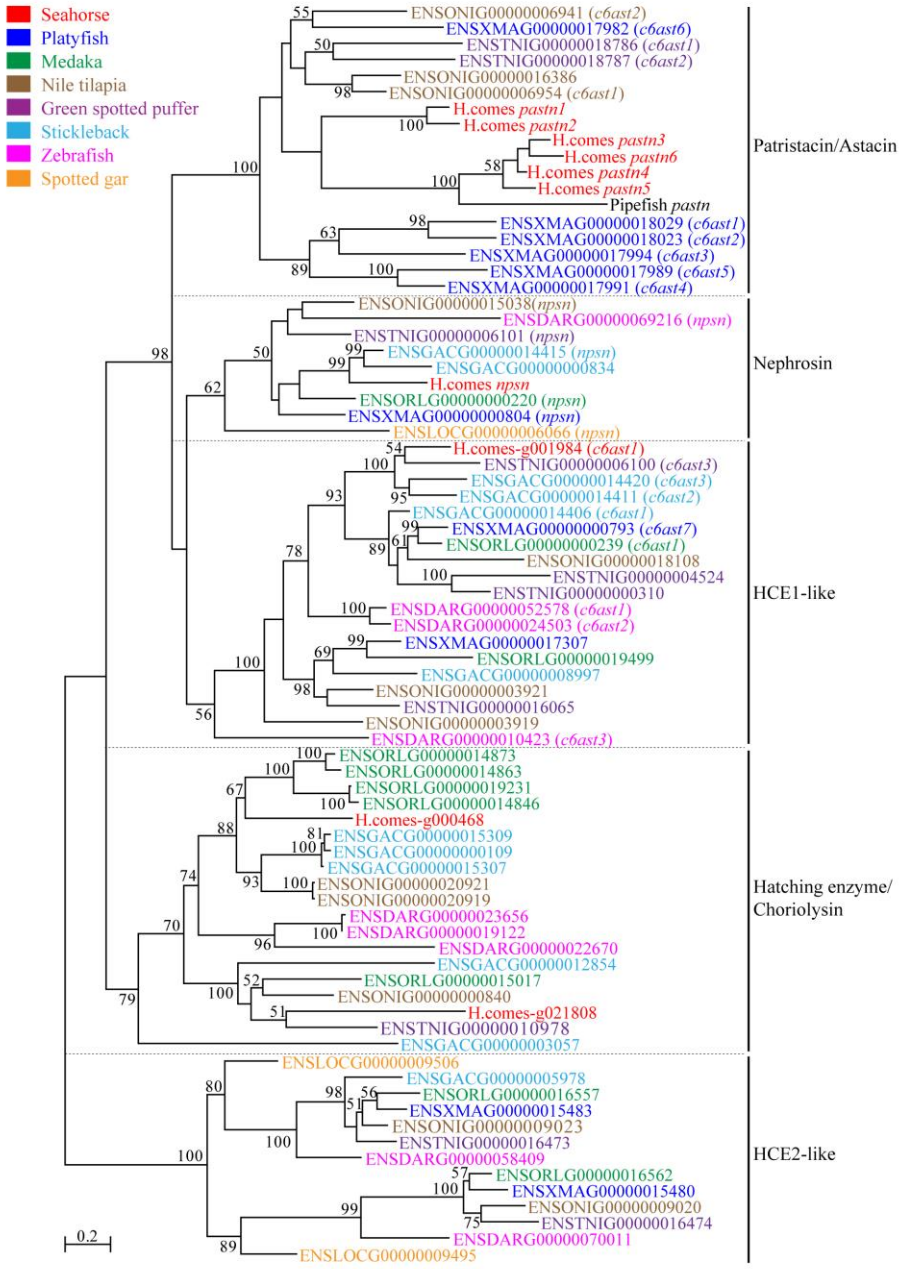

Supplementary Figure 10.1 Phylogenetic tree of astacin family genes in teleosts. The c6ast proteins in the teleost fishes were aligned using the online version Muscle. The phylogeny tree was constructed using PhyML software with the model of “WAG +gamma”.

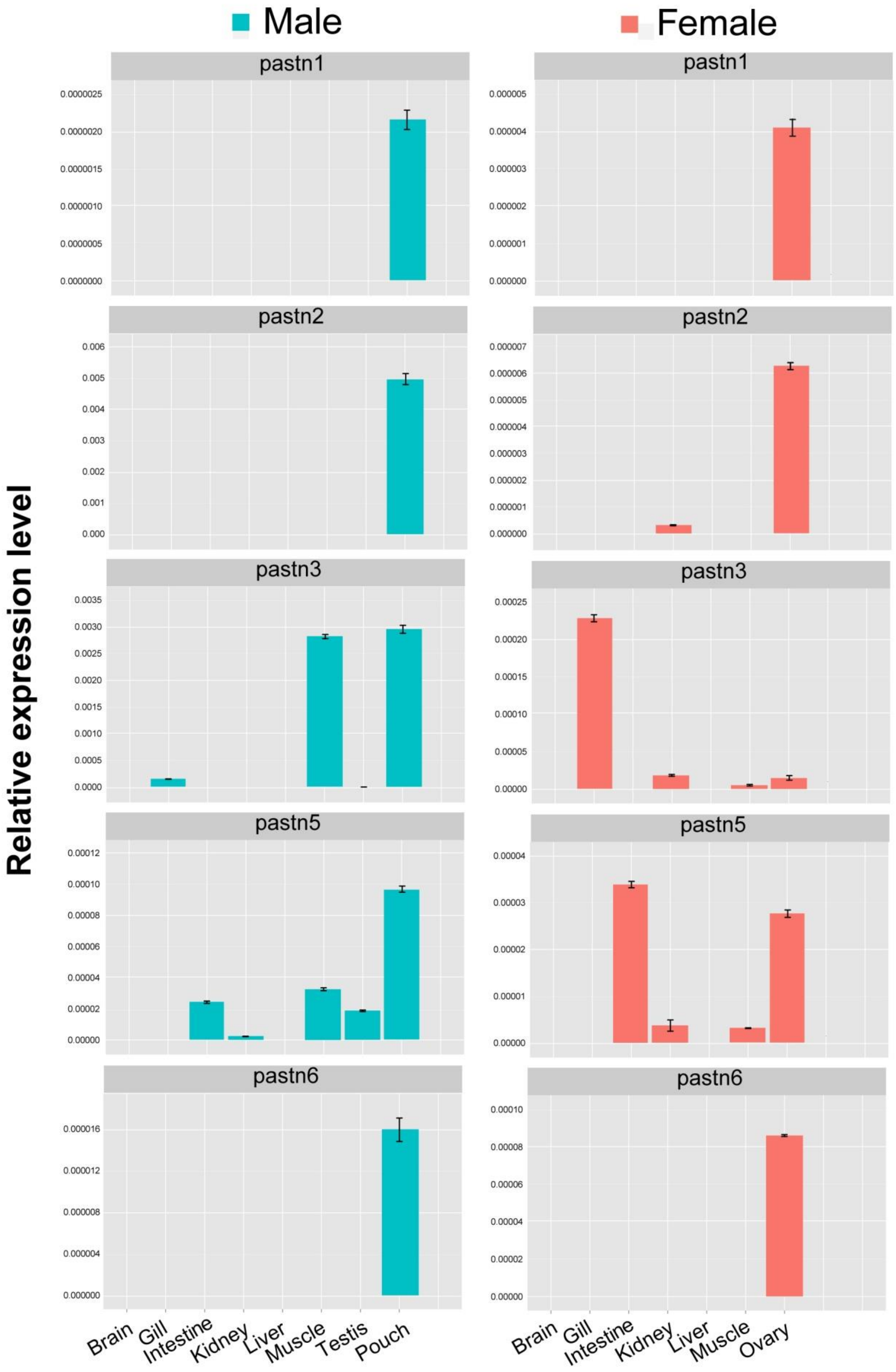

Supplementary Figure 10.2 The expression profile of *pastn* genes in seahorse (*Hippocampus erectus*). The gene expression levels were quantified by real-time PCR

doi:10.1038/nature20595

and the results were expressed as copy numbers normalized by the mean copy number from the reference gene 18S rRNA.

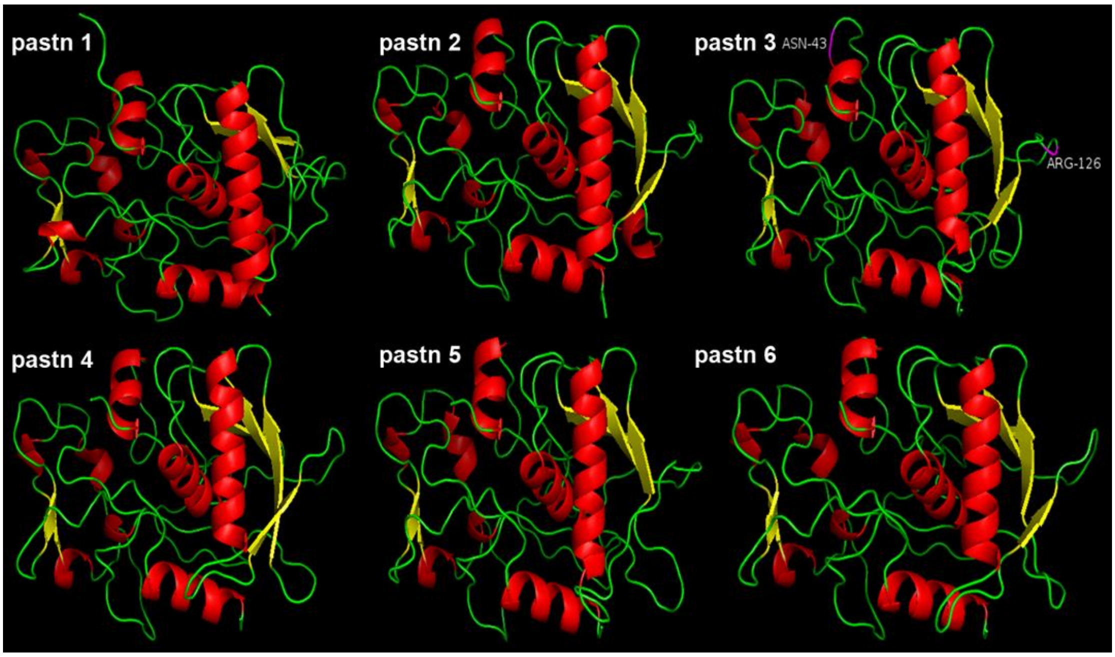

Supplementary Figure 10.3 3D models of Patristacin proteins. Amino acids under positive selection are highlighted purple.

Supplementary Table 10.1 Gene IDs of astacin family genes in teleost genomes.

Number	Gene ID	Species
1	ENSDARG00000010423	*Danio rerio*
2	ENSDARG00000019122	*Danio rerio*
3	ENSDARG00000022670	*Danio rerio*
4	ENSDARG00000023656	*Danio rerio*
5	ENSDARG00000024503	*Danio rerio*
6	ENSDARG00000052578	*Danio rerio*
7	ENSDARG00000058409	*Danio rerio*
8	ENSDARG00000069216	*Danio rerio*
9	ENSDARG00000070011	*Danio rerio*
10	ENSGACG00000000109	*Gasterosteus aculeatus*
11	ENSGACG00000000834	*Gasterosteus aculeatus*
12	ENSGACG00000003057	*Gasterosteus aculeatus*
13	ENSGACG00000005978	*Gasterosteus aculeatus*
14	ENSGACG00000008997	*Gasterosteus aculeatus*
15	ENSGACG00000012854	*Gasterosteus aculeatus*

16	ENSGACG00000014406	*Gasterosteus aculeatus*
17	ENSGACG00000014411	*Gasterosteus aculeatus*
18	ENSGACG00000014415	*Gasterosteus aculeatus*
19	ENSGACG00000014420	*Gasterosteus aculeatus*
20	ENSGACG00000015307	*Gasterosteus aculeatus*
21	ENSGACG00000015309	*Gasterosteus aculeatus*
22	ENSLOCG00000006066	*Lepisosteus oculatus*
23	ENSLOCG00000009495	*Lepisosteus oculatus*
24	ENSLOCG00000009506	*Lepisosteus oculatus*
25	ENSONIG00000000840	*Oreochromis niloticus*
26	ENSONIG00000003919	*Oreochromis niloticus*
27	ENSONIG00000003921	*Oreochromis niloticus*
28	ENSONIG00000006941	*Oreochromis niloticus*
29	ENSONIG00000006954	*Oreochromis niloticus*
30	ENSONIG00000009020	*Oreochromis niloticus*
31	ENSONIG00000009023	*Oreochromis niloticus*
32	ENSONIG00000015038	*Oreochromis niloticus*
33	ENSONIG00000016386	*Oreochromis niloticus*
34	ENSONIG00000018108	*Oreochromis niloticus*
35	ENSONIG00000020919	*Oreochromis niloticus*
36	ENSONIG00000020921	*Oreochromis niloticus*
37	ENSORLG00000000220	*Oryzias latipes*
38	ENSORLG00000000239	*Oryzias latipes*
39	ENSORLG00000014846	*Oryzias latipes*
40	ENSORLG00000014863	*Oryzias latipes*
41	ENSORLG00000014873	*Oryzias latipes*
42	ENSORLG00000015017	*Oryzias latipes*
43	ENSORLG00000016557	*Oryzias latipes*
44	ENSORLG00000016562	*Oryzias latipes*
45	ENSORLG00000019231	*Oryzias latipes*
46	ENSORLG00000019499	*Oryzias latipes*
47	ENSTNIG00000000310	*Tetraodon nigroviridis*
48	ENSTNIG00000004524	*Tetraodon nigroviridis*
49	ENSTNIG00000006100	*Tetraodon nigroviridis*
50	ENSTNIG00000006101	*Tetraodon nigroviridis*
51	ENSTNIG00000010978	*Tetraodon nigroviridis*
52	ENSTNIG00000016065	*Tetraodon nigroviridis*
53	ENSTNIG00000016473	*Tetraodon nigroviridis*
54	ENSTNIG00000016474	*Tetraodon nigroviridis*
55	ENSTNIG00000018786	*Tetraodon nigroviridis*
56	ENSTNIG00000018787	*Tetraodon nigroviridis*
57	ENSXMAG00000000793	*Xiphophorus maculatus*

58	ENSXMAG00000000804	*Xiphophorus maculatus*
59	ENSXMAG00000015480	*Xiphophorus maculatus*
60	ENSXMAG00000015483	*Xiphophorus maculatus*
61	ENSXMAG00000017307	*Xiphophorus maculatus*
62	ENSXMAG00000017982	*Xiphophorus maculatus*
63	ENSXMAG00000017989	*Xiphophorus maculatus*
64	ENSXMAG00000017991	*Xiphophorus maculatus*
65	ENSXMAG00000017994	*Xiphophorus maculatus*
66	ENSXMAG00000018023	*Xiphophorus maculatus*
67	ENSXMAG00000018029	*Xiphophorus maculatus*
68	ABK80843.1	*Syngnathid scovelli*
69	H.comes.g013955	*Hippocampus comes*
70	H.comes.g01395D2	*Hippocampus comes*
71	H.comes.g01395D3	*Hippocampus comes*
72	H.comes.g01395D4	*Hippocampus comes*
73	H.comes.g013953	*Hippocampus comes*
74	H.comes.g013953D2	*Hippocampus comes*
75	H.comes.npsn	*Hippocampus comes*
76	H.comes.g001984	*Hippocampus comes*
77	H.comes.g021808	*Hippocampus comes*
78	H.comes.g000468	*Hippocampus comes*

Supplementary Table 10.2 Selection pressure on *patristacin* genes in seahorse (*H. comes*) and pipefish.

Gene name	ωf	Null	Alternative	LRT	P-value
*pastn*1	6.93564	-13439.46497	-13439.32634	0.277248	NS
*pastn*2	1.12997	-13439.5027	-13439.50041	0.004588	NS
*pastn*3	33.50007	-13439.15397	-13434.53619	9.235566	<0.01
*pastn*4	29.45181	-13439.86765	-13438.69947	2.336372	NS
*pastn*5	3.76168	-13439.53419	-13439.39599	0.276392	NS
*pastn*6	1	-13440.02094	-13440.02094	0	NS
pastn_S.scovelli	1	-13440.020942	-13440.020942	0	NS

ωf: selection pressure on the foreground branch
Null: likelihood under null hypothesis
Alternative: likelihood under alternative hypothesis
LRT: likelihood ratio test
NS: "Not Significance" with P-value > 0.05

11. References

1 Li, R. Q. *et al.* The sequence and de novo assembly of the giant panda genome. *Nature* **463**, 311-317, doi:Doi 10.1038/Nature08696 (2010).

2 Luo, R. *et al.* SOAPdenovo2: an empirically improved memory-efficient short-read de novo assembler. *Gigascience* **1**, 18, doi:10.1186/2047-217X-1-18 (2012).

3 Parra, G., Bradnam, K. & Korf, I. CEGMA: a pipeline to accurately annotate core genes in eukaryotic genomes. *Bioinformatics* **23**, 1061-1067, doi:10.1093/bioinformatics/btm071 (2007).

4 Li, H. & Durbin, R. Fast and accurate short read alignment with Burrows-Wheeler transform. *Bioinformatics* **25**, 1754-1760, doi:10.1093/bioinformatics/btp324 (2009).

5 Grabherr, M. G. *et al.* Full-length transcriptome assembly from RNA-Seq data without a reference genome. *Nature Biotechnology* **29**, 644-U130, doi:Doi 10.1038/Nbt.1883 (2011).

6 Kent, W. J. BLAT - The BLAST-like alignment tool. *Genome Research* **12**, 656-664, doi:Doi 10.1101/Gr.229202 (2002).

7 Trapnell, C., Pachter, L. & Salzberg, S. L. TopHat: discovering splice junctions with RNA-Seq. *Bioinformatics* **25**, 1105-1111, doi:DOI 10.1093/bioinformatics/btp120 (2009).

8 Benson, G. Tandem repeats finder: a program to analyze DNA sequences. *Nucleic Acids Res* **27**, 573-580, doi:Doi 10.1093/Nar/27.2.573 (1999).

9 Jurka, J. *et al.* Repbase update, a database of eukaryotic repetitive elements. *Cytogenet Genome Res* **110**, 462-467, doi:Doi 10.1159/000084979 (2005).

10 Bao, Z. & Eddy, S. R. Automated de novo identification of repeat sequence families in sequenced genomes. *Genome Research* **12**, 1269-1276, doi:10.1101/gr.88502 (2002).

11 Price, A. L., Jones, N. C. & Pevzner, P. A. De novo identification of repeat families in large genomes. *Bioinformatics* **21**, I351-I358, doi:DOI 10.1093/bioinformatics/bti1018 (2005).

12 Kimura, M. A simple method for estimating evolutionary rates of base substitutions through comparative studies of nucleotide sequences. *Journal of Molecular Evolution* **16**, 111-120 (1980).

13 Volff, J. N. Genome evolution and biodiversity in teleost fish. *Heredity* **94**, 280-294, doi:Doi 10.1038/Sj.Hdy.6800635 (2005).

14 Howe, K. *et al.* The zebrafish reference genome sequence and its relationship to the human genome. *Nature* **496**, 498-503, doi:10.1038/nature12111 (2013).

15 Aparicio, S. *et al.* Whole-genome shotgun assembly and analysis of the genome of Fugu rubripes. *Science* **297**, 1301-1310, doi:10.1126/science.1072104 (2002).

16 Kasahara, M. *et al.* The medaka draft genome and insights into vertebrate genome evolution. *Nature* **447**, 714-719, doi:Doi 10.1038/Nature05846 (2007).

17 Star, B. *et al.* The genome sequence of Atlantic cod reveals a unique immune system. *Nature* **477**, 207-210, doi:Doi 10.1038/Nature10342 (2011).

18 Brawand, D. *et al.* The genomic substrate for adaptive radiation in African cichlid fish. *Nature* **513**, 375-381, doi:10.1038/nature13726 (2014).

19 Schartl, M. *et al.* The genome of the platyfish, Xiphophorus maculatus, provides insights into evolutionary adaptation and several complex traits. *Nature Genetics* **45**, 567-572,

doi:10.1038/ng.2604 (2013).

20 Jones, F. C. *et al.* The genomic basis of adaptive evolution in threespine sticklebacks. *Nature* **484**, 55-61, doi:10.1038/nature10944 (2012).

21 You, X. *et al.* Mudskipper genomes provide insights into the terrestrial adaptation of amphibious fishes. *Nature communications* **5**, 5594, doi:10.1038/ncomms6594 (2014).

22 Trapnell, C. *et al.* Transcript assembly and quantification by RNA-Seq reveals unannotated transcripts and isoform switching during cell differentiation. *Nature Biotechnology* **28**, 511-U174, doi:Doi 10.1038/Nbt.1621 (2010).

23 Bairoch, A. & Apweiler, R. The SWISS-PROT protein sequence database and its supplement TrEMBL in 2000. *Nucleic Acids Res* **28**, 45-48 (2000).

24 Kanehisa, M., Sato, Y., Kawashima, M., Furumichi, M. & Tanabe, M. KEGG as a reference resource for gene and protein annotation. *Nucleic Acids Res*, doi:10.1093/nar/gkv1070 (2015).

25 Mitchell, A. *et al.* The InterPro protein families database: the classification resource after 15 years. *Nucleic Acids Res* **43**, D213-221, doi:10.1093/nar/gku1243 (2015).

26 Ashburner, M. *et al.* Gene ontology: tool for the unification of biology. The Gene Ontology Consortium. *Nature Genetics* **25**, 25-29, doi:10.1038/75556 (2000).

27 Conesa, A. *et al.* Blast2GO: a universal tool for annotation, visualization and analysis in functional genomics research. *Bioinformatics* **21**, 3674-3676, doi:doi: 10.1093/bioinformatics/bti610 (2005).

28 Gotz, S. *et al.* High-throughput functional annotation and data mining with the Blast2GO suite. *Nucleic Acids Res* **36**, 3420-3435, doi:10.1093/nar/gkn176 (2008).

29 Yu, X., Lin, J., Zack, D. J. & Qian, J. Computational analysis of tissue-specific combinatorial gene regulation: predicting interaction between transcription factors in human tissues. *Nucleic Acids Res* **34**, 4925-4936, doi:10.1093/nar/gkl595 (2006).

30 Tarazona, S., Garcia-Alcalde, F., Dopazo, J., Ferrer, A. & Conesa, A. Differential expression in RNA-seq: A matter of depth. *Genome Research* **21**, 2213-2223, doi:DOI 10.1101/gr.124321.111 (2011).

31 Small, C. M., Harlin-Cognato, A. D. & Jones, A. G. Functional similarity and molecular divergence of a novel reproductive transcriptome in two male-pregnant Syngnathus pipefish species. *Ecology and evolution* **3**, 4092-4108, doi:10.1002/ece3.763 (2013).

32 Hubbard, T. *et al.* The Ensembl genome database project. *Nucleic Acids Res* **30**, 38-41 (2002).

33 Altschul, S. F., Gish, W., Miller, W., Myers, E. W. & Lipman, D. J. Basic local alignment search tool. *Journal of Molecular Biology* **215**, 403-410, doi:10.1016/S0022-2836(05)80360-2 (1990).

34 Edgar, R. C. MUSCLE: multiple sequence alignment with high accuracy and high throughput. *Nucleic Acids Res* **32**, 1792-1797, doi:Doi 10.1093/Nar/Gkh340 (2004).

35 Capella-Gutierrez, S., Silla-Martinez, J. M. & Gabaldon, T. trimAl: a tool for automated alignment trimming in large-scale phylogenetic analyses. *Bioinformatics* **25**, 1972-1973, doi:10.1093/bioinformatics/btp348 (2009).

36 Stamatakis, A. RAxML-VI-HPC: Maximum likelihood-based phylogenetic analyses with thousands of taxa and mixed models. *Bioinformatics* **22**, 2688-2690, doi:Doi 10.1093/Bioinformatics/Btl446 (2006).

37 Stamatakis, A., Hoover, P. & Rougemont, J. A Rapid Bootstrap Algorithm for the RAxML Web Servers. *Syst Biol* **57**, 758-771, doi:Doi 10.1080/10635150802429642 (2008).

38 Paradis, E., Claude, J. & Strimmer, K. APE: Analyses of Phylogenetics and Evolution in R language. *Bioinformatics* **20**, 289-290 (2004).

39 Tajima, F. Statistical method for testing the neutral mutation hypothesis by DNA polymorphism. *Genetics* **123**, 585-595 (1989).

40 Nei, M. & Kumar, S. *Molecular evolution and phylogenetics*. (Oxford University Press, 2000).

41 Takezaki, N., Rzhetsky, A. & Nei, M. Phylogenetic test of the molecular clock and linearized trees. *Molecular Biology and Evolution* **12**, 823-833 (1995).

42 Zhang, J., Nielsen, R. & Yang, Z. Evaluation of an improved branch-site likelihood method for detecting positive selection at the molecular level. *Molecular Biology and Evolution* **22**, 2472-2479, doi:10.1093/molbev/msi237 (2005).

43 Yang, Z. PAML 4: phylogenetic analysis by maximum likelihood. *Molecular Biology and Evolution* **24**, 1586-1591, doi:doi: 10.1093/molbev/msm088 (2007).

44 Harris, R. S. Improved pairwise alignment of genomic DNA. *The Pennsylvania State University, The Graduate School, College of Engineering* (2007).

45 Blanchette, M. *et al.* Aligning multiple genomic sequences with the threaded blockset aligner. *Genome Research* **14**, 708-715, doi:10.1101/gr.1933104 (2004).

46 Betancur, R. R. *et al.* The tree of life and a new classification of bony fishes. *PLoS Curr* **5**, doi:10.1371/currents.tol.53ba26640df0ccaee75bb165c8c26288 (2013).

47 Near, T. J. *et al.* Phylogeny and tempo of diversification in the superradiation of spiny-rayed fishes. *Proc Natl Acad Sci U S A* **110**, 12738-12743, doi:10.1073/pnas.1304661110 (2013).

48 Hubisz, M. J., Pollard, K. S. & Siepel, A. PHAST and RPHAST: phylogenetic analysis with space/time models. *Briefings in Bioinformatics* **12**, 41-51, doi:10.1093/bib/bbq072 (2011).

49 McLean, C. Y. *et al.* GREAT improves functional interpretation of cis-regulatory regions. *Nature biotechnology* **28**, 495-501, doi:10.1038/nbt.1630 (2010).

50 Brudno, M. *et al.* LAGAN and Multi-LAGAN: efficient tools for large-scale multiple alignment of genomic DNA. *Genome research* **13**, 721-731, doi:10.1101/gr.926603 (2003).

51 Frazer, K. A., Pachter, L., Poliakov, A., Rubin, E. M. & Dubchak, I. VISTA: computational tools for comparative genomics. *Nucleic Acids Res* **32**, W273-279, doi:10.1093/nar/gkh458 (2004).

52 Nelson, J. S. *Fishes of the World*. 4th edn, (John Wiley & Sons, 2006).

53 Sweetman, D. & Munsterberg, A. The vertebrate spalt genes in development and disease. *Dev Biol* **293**, 285-293, doi:10.1016/j.ydbio.2006.02.009 (2006).

54 Farrell, E. R. & Munsterberg, A. E. csal1 is controlled by a combination of FGF and Wnt signals in developing limb buds. *Dev Biol* **225**, 447-458, doi:10.1006/dbio.2000.9852 (2000).

55 Harvey, S. A. & Logan, M. P. sall4 acts downstream of tbx5 and is required for pectoral fin outgrowth. *Development* **133**, 1165-1173, doi:10.1242/dev.02259 (2006).

56 Shears, D. J. *et al.* Mutation and deletion of the pseudoautosomal gene SHOX cause Leri-Weill dyschondrosteosis. *Nature Genetics* **19**, 70-73, doi:10.1038/ng0198-70 (1998).

57 Sabherwal, N. *et al.* Long-range conserved non-coding SHOX sequences regulate expression in developing chicken limb and are associated with short stature phenotypes in human patients. *Hum Mol Genet* **16**, 210-222, doi:10.1093/hmg/ddl470 (2007).

58 Kenyon, E. J., McEwen, G. K., Callaway, H. & Elgar, G. Functional analysis of conserved non-coding regions around the short stature hox gene (shox) in whole zebrafish embryos. *PLoS One* **6**, e21498, doi:10.1371/journal.pone.0021498 (2011).

59 Li, D. *et al.* Formation of proximal and anterior limb skeleton requires early function of Irx3 and Irx5 and is negatively regulated by Shh signaling. *Dev Cell* **29**, 233-240, doi:10.1016/j.devcel.2014.03.001 (2014).

60 Cheng, C. W. *et al.* The Iroquois homeobox gene, Irx5, is required for retinal cone bipolar cell development. *Dev Biol* **287**, 48-60, doi:10.1016/j.ydbio.2005.08.029 (2005).

61 Costantini, D. L. *et al.* The homeodomain transcription factor Irx5 establishes the mouse cardiac ventricular repolarization gradient. *Cell* **123**, 347-358, doi:10.1016/j.cell.2005.08.004 (2005).

62 Bonnard, C. *et al.* Mutations in IRX5 impair craniofacial development and germ cell migration via SDF1. *Nat Genet* **44**, 709-713, doi:10.1038/ng.2259 (2012).

63 Alexander, T., Nolte, C. & Krumlauf, R. Hox genes and segmentation of the hindbrain and axial skeleton. *Annu Rev Cell Dev Biol* **25**, 431-456, doi:10.1146/annurev.cellbio.042308.113423 (2009).

64 Zakany, J. & Duboule, D. The role of Hox genes during vertebrate limb development. *Curr Opin Genet Dev* **17**, 359-366, doi:10.1016/j.gde.2007.05.011 (2007).

65 Lynch, V. J. *et al.* Adaptive changes in the transcription factor HoxA-11 are essential for the evolution of pregnancy in mammals. *P Natl Acad Sci USA* **105**, 14928-14933, doi:10.1073/pnas.0802355105 (2008).

66 Yang, Z. H., Wong, W. S. W. & Nielsen, R. Bayes empirical Bayes inference of amino acid sites under positive selection. *Molecular Biology and Evolution* **22**, 1107-1118, doi:DOI 10.1093/molbev/msi097 (2005).

67 Couly, G., Creuzet, S., Bennaceur, S., Vincent, C. & Le Douarin, N. M. Interactions between Hox-negative cephalic neural crest cells and the foregut endoderm in patterning the facial skeleton in the vertebrate head. *Development* **129**, 1061-1073 (2002).

68 Oury, F. *et al.* Hoxa2- and rhombomere-dependent development of the mouse facial somatosensory map. *Science* **313**, 1408-1413, doi:10.1126/science.1130042 (2006).

69 Bobola, N. *et al.* Mesenchymal patterning by Hoxa2 requires blocking Fgf-dependent activation of Ptx1. *Development* **130**, 3403-3414 (2003).

70 Kanzler, B., Kuschert, S. J., Liu, Y. H. & Mallo, M. Hoxa-2 restricts the chondrogenic domain and inhibits bone formation during development of the branchial area. *Development* **125**, 2587-2597 (1998).

71 Donaldson, I. J. *et al.* Genome-wide occupancy links Hoxa2 to Wnt-beta-catenin signaling in mouse embryonic development. *Nucleic Acids Res* **40**, 3990-4001, doi:10.1093/nar/gkr1240 (2012).

72 Horan, G. S., Wu, K., Wolgemuth, D. J. & Behringer, R. R. Homeotic transformation of cervical vertebrae in Hoxa-4 mutant mice. *P Natl Acad Sci USA* **91**, 12644-12648 (1994).

73 Niimura, Y. & Nei, M. Evolutionary dynamics of olfactory receptor genes in fishes and tetrapods. *P Natl Acad Sci USA* **102**, 6039-6044, doi:DOI 10.1073/pnas.0501922102 (2005).

74 Niimura, Y. On the Origin and Evolution of Vertebrate Olfactory Receptor Genes: Comparative

Genome Analysis Among 23 Chordate Species. *Genome Biol Evol* **1**, 34-44, doi:Doi 10.1093/Gbe/Evp003 (2009).
75 Niimura, Y. Evolutionary dynamics of olfactory receptor genes in chordates: interaction between environments and genomic contents. *Human genomics* **4**, 107 (2009).
76 Lindqvist, C., Sundin, J., Berglund, A. & Rosenqvist, G. Male broad-nosed pipefish Syngnathus typhle do not locate females by smell. *Journal of fish biology* **78**, 1861-1867, doi:10.1111/j.1095-8649.2011.02985.x (2011).
77 Infante, C. R. *et al.* Shared Enhancer Activity in the Limbs and Phallus and Functional Divergence of a Limb-Genital cis-Regulatory Element in Snakes. *Developmental cell* **35**, 107-119, doi:10.1016/j.devcel.2015.09.003 (2015).
78 Menke, D. B., Guenther, C. & Kingsley, D. M. Dual hindlimb control elements in the Tbx4 gene and region-specific control of bone size in vertebrate limbs. *Development* **135**, 2543-2553, doi:10.1242/dev.017384 (2008).
79 Jao, L. E., Wente, S. R. & Chen, W. Efficient multiplex biallelic zebrafish genome editing using a CRISPR nuclease system. *Proc Natl Acad Sci U S A* **110**, 13904-13909, doi:10.1073/pnas.1308335110 (2013).
80 Don, E. K. *et al.* Genetic basis of hindlimb loss in a naturally occurring vertebrate model. *Biol Open*, doi:bio.016295 [pii]
10.1242/bio.016295 (2016).
81 Winterbottom, R. Descriptive Synonymy of Striated Muscles of Teleostei. *P Acad Nat Sci Phila* **125**, 225-317 (1973).
82 Neutens, C. *et al.* Grasping convergent evolution in syngnathids: a unique tale of tails. *J Anat* **224**, 710-723, doi:10.1111/joa.12181 (2014).
83 Kawasaki, K., Buchanan, A. V. & Weiss, K. M. Gene duplication and the evolution of vertebrate skeletal mineralization. *Cells Tissues Organs* **186**, 7-24, doi:10.1159/000102678 (2007).
84 Kawasaki, K. The SCPP gene family and the complexity of hard tissues in vertebrates. *Cells Tissues Organs* **194**, 108-112, doi:10.1159/000324225 (2011).
85 Venkatesh, B. *et al.* Elephant shark genome provides unique insights into gnathostome evolution. *Nature* **505**, 174-179, doi:10.1038/nature12826 (2014).
86 Kawasaki, K. & Amemiya, C. T. SCPP genes in the coelacanth: tissue mineralization genes shared by sarcopterygians. *J Exp Zool B Mol Dev Evol* **322**, 390-402 (2014).
87 Kawasaki, K. The SCPP gene repertoire in bony vertebrates and graded differences in mineralized tissues. *Development genes and evolution* **219**, 147-157, doi:10.1007/s00427-009-0276-x (2009).
88 Louchart, A. & Viriot, L. From snout to beak: the loss of teeth in birds. *Trends Ecol Evol* **26**, 663-673, doi:10.1016/j.tree.2011.09.004 (2011).
89 Meredith, R. W., Zhang, G., Gilbert, M. T., Jarvis, E. D. & Springer, M. S. Evidence for a single loss of mineralized teeth in the common avian ancestor. *Science* **346**, 1254390, doi:10.1126/science.1254390 (2014).
90 Demere, T. A., McGowen, M. R., Berta, A. & Gatesy, J. Morphological and molecular evidence for a stepwise evolutionary transition from teeth to baleen in mysticete whales. *Syst Biol* **57**, 15-37, doi:10.1080/10635150701884632 (2008).

91 Zhang, G. *et al.* Comparative genomics reveals insights into avian genome evolution and adaptation. *Science* **346**, 1311-1320, doi:10.1126/science.1251385 (2014).

92 Solovyev, V., Kosarev, P., Seledsov, I. & Vorobyev, D. Automatic annotation of eukaryotic genes, pseudogenes and promoters. *Genome Biol* **7 Suppl 1**, S10 11-12, doi:10.1186/gb-2006-7-s1-s10 (2006).

93 Edgar, R. C. MUSCLE: multiple sequence alignment with high accuracy and high throughput. *Nucleic acids research* **32**, 1792-1797, doi:10.1093/nar/gkh340 (2004).

94 Guindon, S. *et al.* New algorithms and methods to estimate maximum-likelihood phylogenies: assessing the performance of PhyML 3.0. *Syst Biol* **59**, 307-321, doi:10.1093/sysbio/syq010 (2010).
