# Supplementary figures and images for: The seahorse genome and the evolution of its specialized morphology
Source: Nature. 2016 Dec 14;540(7633):395–9. doi: 10.1038/nature20595 (PMC8127814; doi:10.1038/nature20595)

## Slide 1
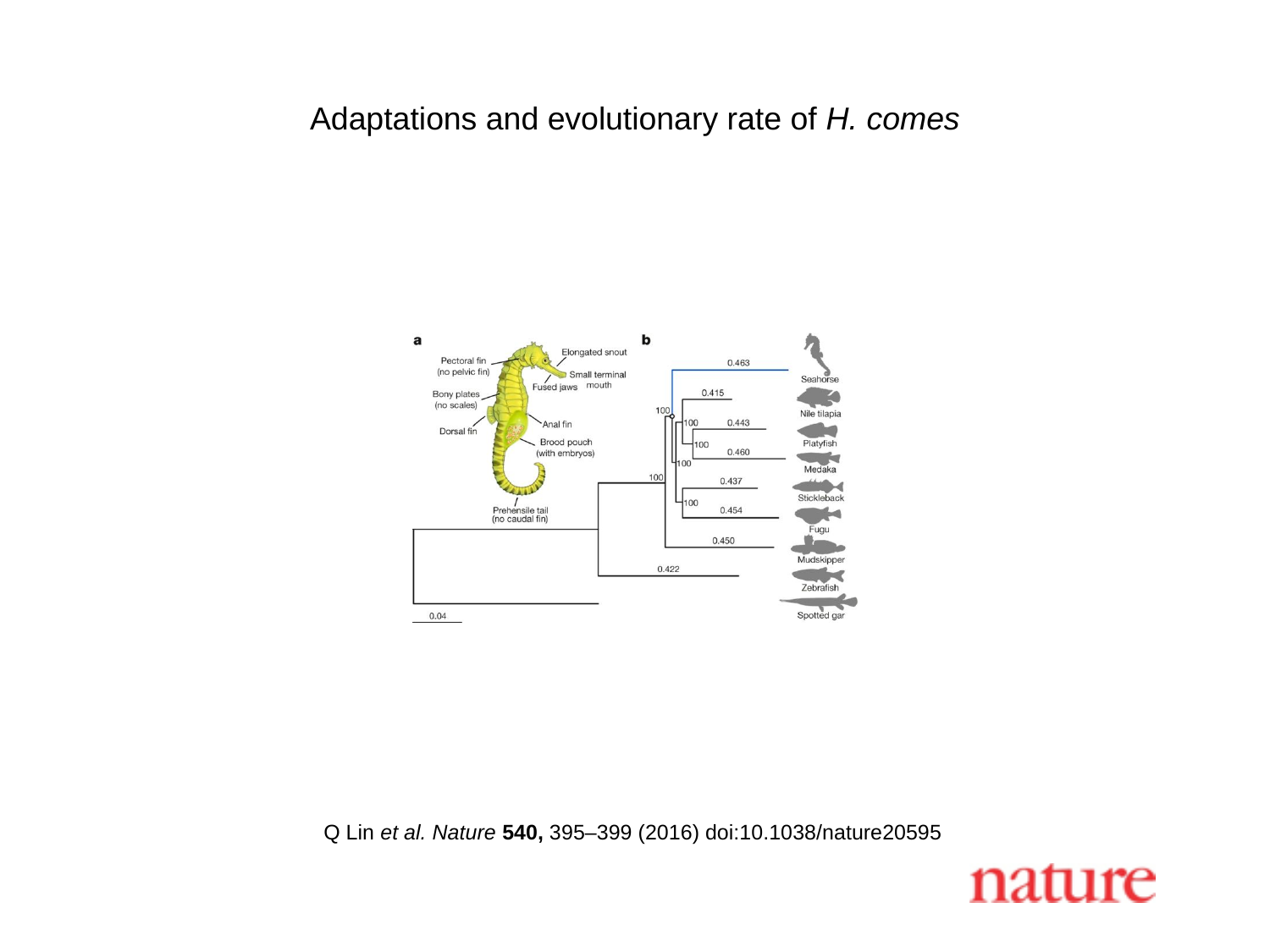

# Adaptations and evolutionary rate of H. comes
Q Lin et al. Nature 540, 395–399 (2016) doi:10.1038/nature20595

Supplement: Supplementary file 3 — PowerPoint slide for Fig. 1 [file 41586_2016_BFnature20595_MOESM64_ESM.ppt]

## Slide 1
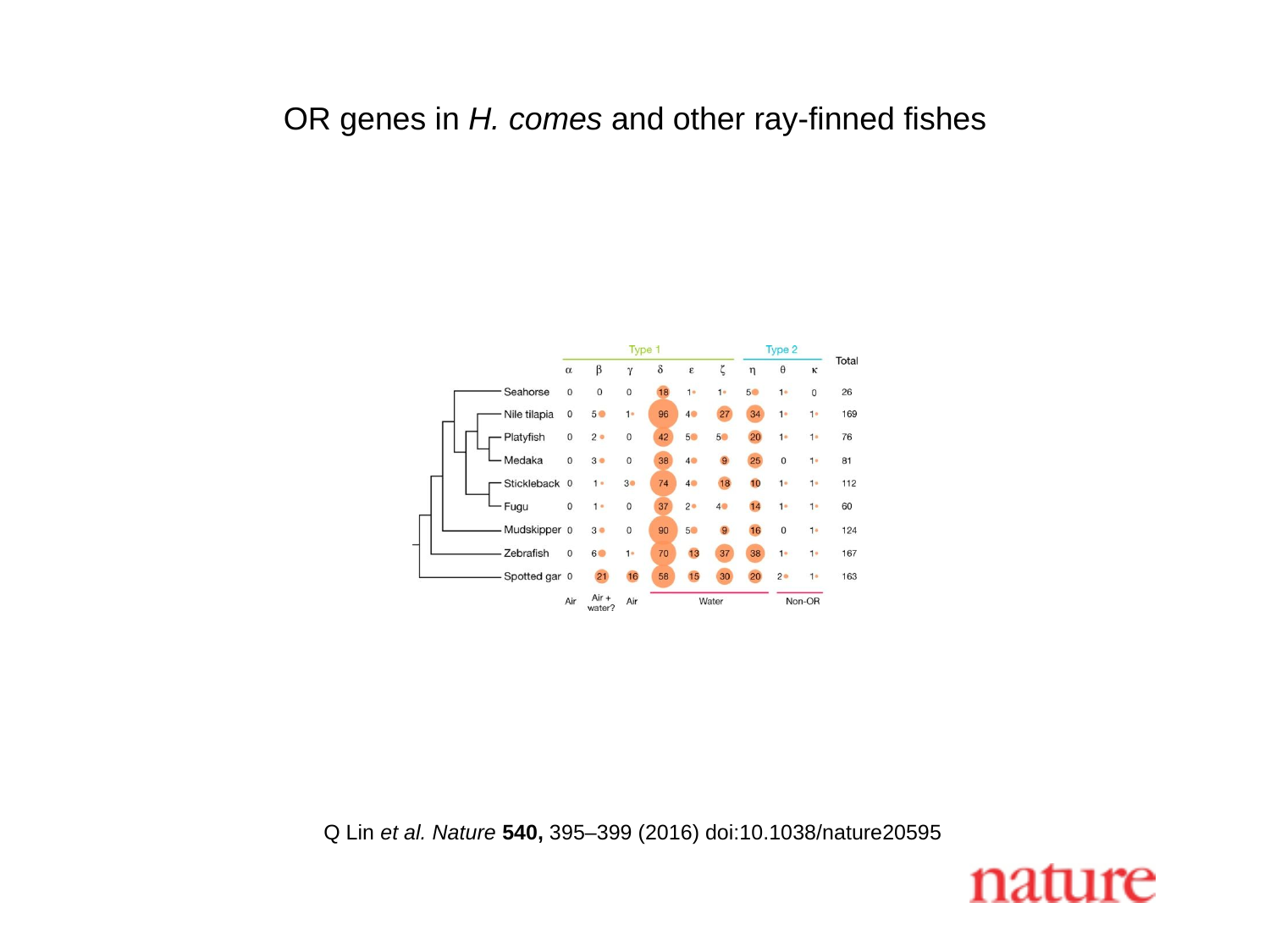

# OR genes in H. comes and other ray-finned fishes
Q Lin et al. Nature 540, 395–399 (2016) doi:10.1038/nature20595

Supplement: Supplementary file 4 — PowerPoint slide for Fig. 2 [file 41586_2016_BFnature20595_MOESM65_ESM.ppt]

## Slide 1
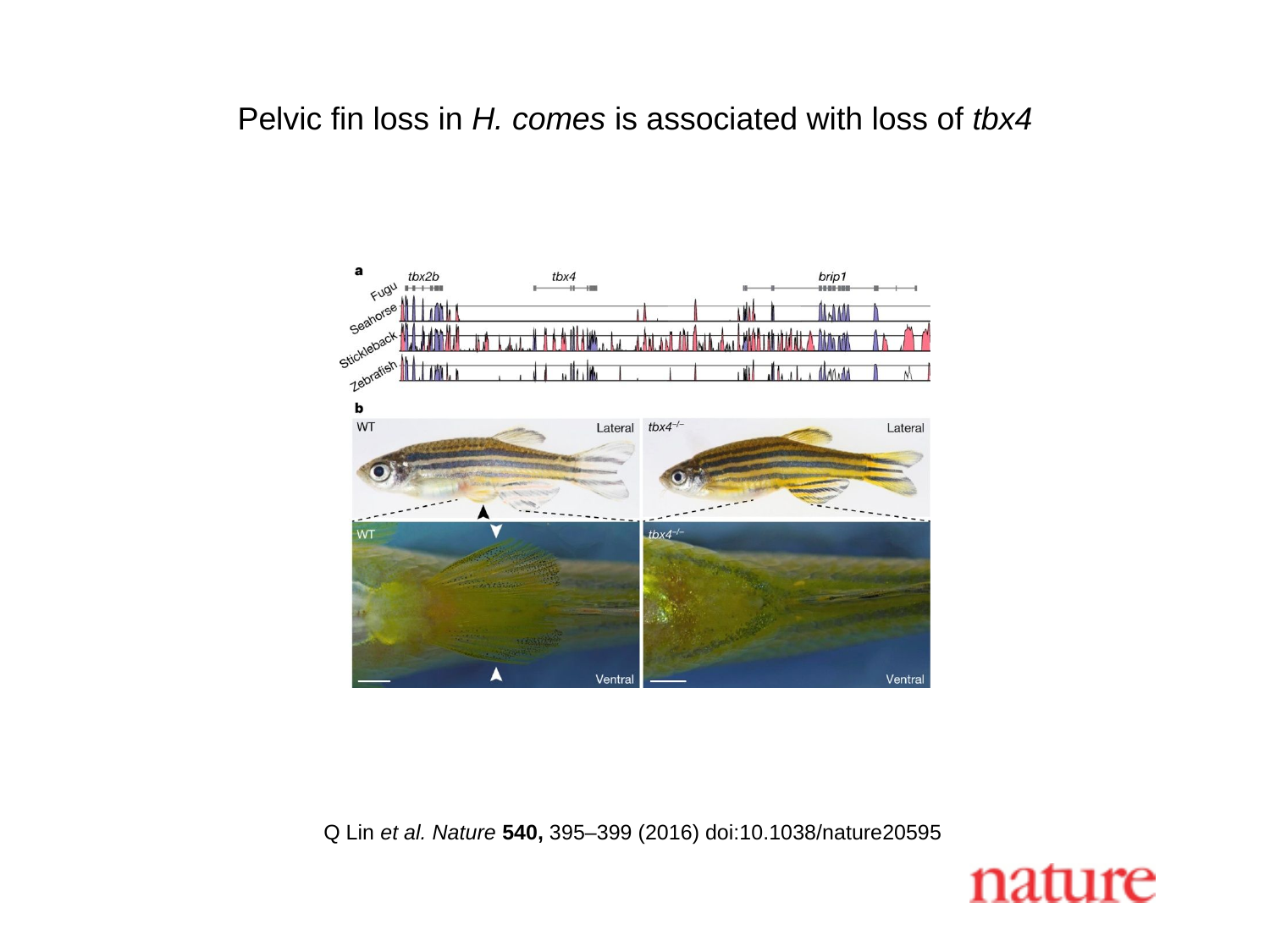

# Pelvic fin loss in H. comes is associated with loss of tbx4
Q Lin et al. Nature 540, 395–399 (2016) doi:10.1038/nature20595

Supplement: Supplementary file 5 — PowerPoint slide for Fig. 3 [file 41586_2016_BFnature20595_MOESM66_ESM.ppt]

## Slide 1
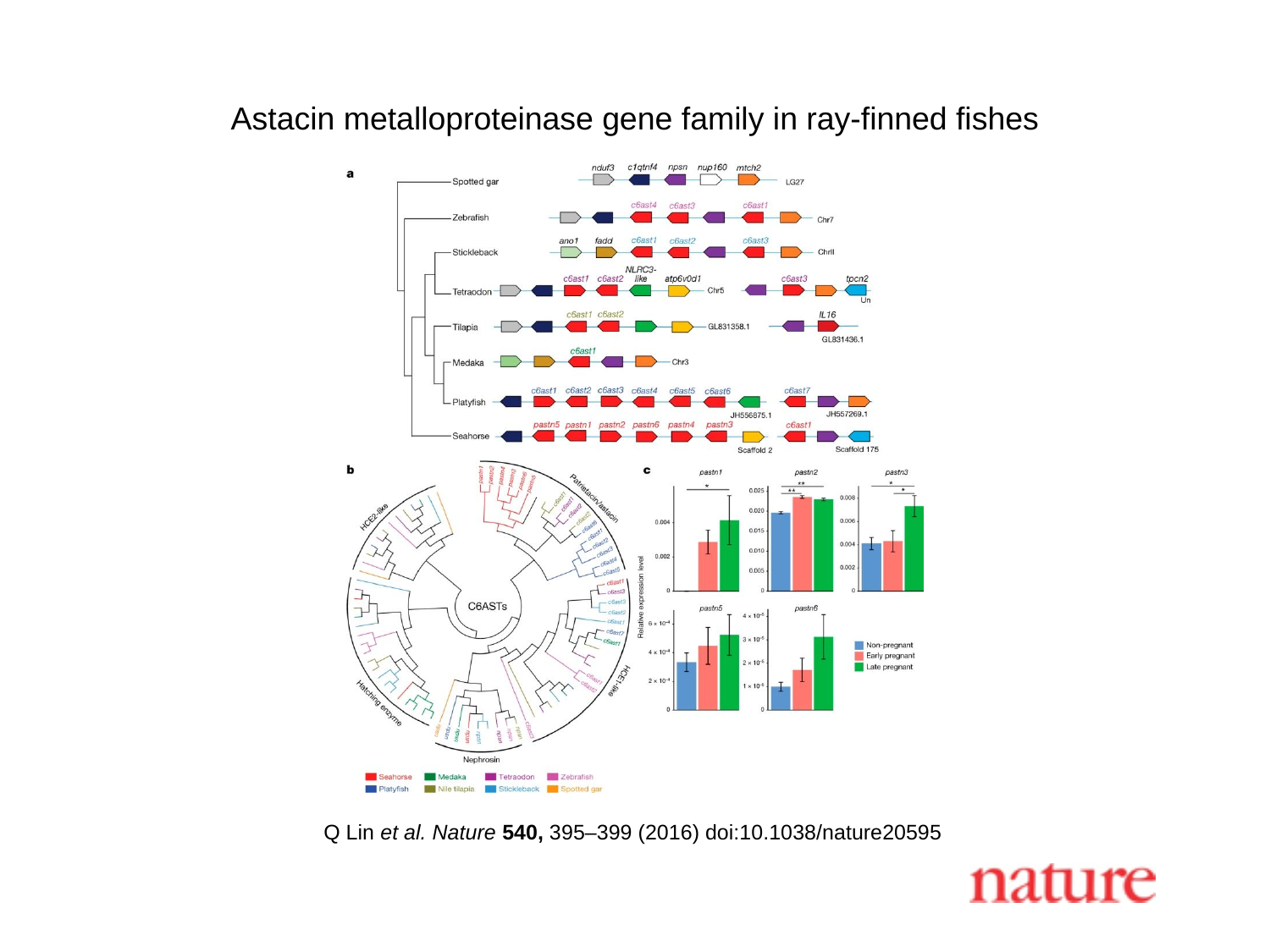

# Astacin metalloproteinase gene family in ray-finned fishes
Q Lin et al. Nature 540, 395–399 (2016) doi:10.1038/nature20595

Supplement: Supplementary file 6 — PowerPoint slide for Fig. 4 [file 41586_2016_BFnature20595_MOESM67_ESM.ppt]
